# Simultaneous Purification of Human Interferon Alpha-2b and Serum Albumin Using Bioprivileged Fluorinated Ionic Liquid-Based Aqueous Biphasic Systems

**DOI:** 10.3390/ijms25052751

**Published:** 2024-02-27

**Authors:** Sara F. Carvalho, Ana B. Pereiro, João M. M. Araújo

**Affiliations:** LAQV, REQUIMTE, Department of Chemistry, NOVA School of Science and Technology, NOVA University Lisbon, 2829-516 Caparica, Portugal; sfe.carvalho@campus.fct.unl.pt (S.F.C.); anab@fct.unl.pt (A.B.P.)

**Keywords:** human IFN-α2b, serum albumin, BSA, bioactive proteins, aqueous biphasic systems, bioprivileged fluorinated ionic liquids, purification

## Abstract

Interferon alpha-2b (IFN-α2b) is an essential cytokine widely used in the treatment of chronic hepatitis C and hairy cell leukemia, and serum albumin is the most abundant plasma protein with numerous physiological functions. Effective single-step aqueous biphasic system (ABS) extraction for the simultaneous purification of IFN-α2b and BSA (serum albumin protein) was developed in this work. Effects of the ionic liquid (IL)-based ABS functionalization, fluorinated ILs (FILs; [C${}_{2}$C${}_{1}$Im][C${}_{4}$F${}_{9}$SO${}_{3}$] and [N${}_{1112(\mathrm{OH})}$][C${}_{4}$F${}_{9}$SO${}_{3}$]) vs. mere fluoro-containing IL ([C${}_{4}$C${}_{1}$Im][CF${}_{3}$SO${}_{3}$]), in combination with sucrose or [N${}_{1112(\mathrm{OH})}$][H${}_{2}$PO${}_{4}$] (well-known globular protein stabilizers), or high-charge-density salt K${}_{3}$PO${}_{4}$ were investigated. The effects of phase pH, phase water content (%wt), phase composition (%wt), and phase volume ratio were investigated. The phase pH was found to have a significant effect on IFN-α2b and BSA partition. Experimental results show that simultaneous single-step purification was achieved with a high yield (extraction efficiency up to 100%) for both proteins and a purification factor of IFN-α2b high in the enriched IFN-α2b phase (up to 23.22) and low in the BSA-enriched phase (down to 0.00). SDS-PAGE analysis confirmed the purity of both recovered proteins. The stability and structure of IFN-α2b and BSA were preserved or even improved (FIL-rich phase) during the purification step, as evaluated by CD spectroscopy and DSC. Binding studies of IFN-α2b and BSA with the ABS phase-forming components were assessed by MST, showing the strong interaction between FILs aggregates and both proteins. In view of their biocompatibility, customizable properties, and selectivity, FIL-based ABSs are suggested as an improved purification step that could facilitate the development of biologics.

## 1. Introduction

Interferons (IFNs) belong to the family of cytokines that exert antiviral, antiproliferative, and immunoregulatory activities in the human body [1,2]. The molecular understanding and therapeutic application of IFNs was a major achievement in biomedicine since their discovery in 1957 by Isaacs and Lindenmann [3]. Human interferons are classified as either type I (alpha, beta, epsilon, omega) or type II (gamma) based on their sequence composition and receptor binding. Human interferon alpha-2 is one of the most important members of the type I interferon cytokine family and comprises three alleles: IFN alpha-2a, IFN alpha-2b, and IFN alpha-2c [4]. Interferon alpha-2b (IFN-α2b), the one considered in this work, is synthesized from the human IFN-α2b gene on human chromosome 9 [5]. IFN-α2b is produced by recombinant DNA technology using different host systems like *Escherichia coli*, *Bacillus subtilis*, *Lactococcus lactistext*, *Pichia pastoristext*, *Saccharomyces cerevisiae*, *Trichoderma reesei*, *Yarrowia lipolytica*, and mammalian cells [6]. Recombinant IFN-α2b is a single non-glycosylated polypeptide chain with a molecular weight of 19 kDa, comprising 165 amino acids, with four cysteine residues involved in the formation of two disulfide bonds [7,8]. Currently, there are several key IFN-α2b products on the market used in the treatment of several diseases, including melanoma, hairy cell leukemia, non-Hodgkin’s lymphoma, and hepatitis B and C [1]. The expression of recombinant proteins as inclusion bodies offers several advantages but also presents a challenging process; it requires several steps to extract proteins, including solubilization, purification, and refolding, which is well-documented [9,10,11,12,13]. Inclusion bodies contain several impurities, like host cell proteins, such as RNA polymerase, outer membrane proteins, and enzymes, as well as host cell DNA and cell wall particles. The work of Ramanan et al. [14] on the efficacy of a simple laboratory method for cell disruption and the release of total protein and IFN-α2b from *E. coli* demonstrates the presence of serum albumin protein in intracellular proteins. In the preparation of inclusion-body proteins from *E. coli*, the other proteins are either derived from unbroken cells (most likely) or *E. coli* cytoplasmatic proteins co-precipitated or trapped during aggregate formation [15].

Serum albumin is the most abundant plasma protein and has numerous physiological functions, namely maintaining the colloid osmotic pressure of the blood, lipid metabolism, metal ion transport, and binding many therapeutic small molecule drugs [16,17,18]. Human serum albumin (HSA) is used clinically for hemorrhagic shock, hypoproteinemia, severe burn injuries, fetal erythroblastosis, etc. [19,20]. Other applications for HSA include the nanocarrier of drugs [21], the carrier of oxygen [22], and extending the plasma half-life of target proteins [23]. The production of HSA is mainly based on collected human plasma, with all the associated limitations in the supply and potential blood-derived infectious pathogen contamination (such as hepatitis and HIV) [24,25]. Hence, to avoid dependence on pooled human blood products, in the last few decades, the production of HSA was examined by recombinant DNA technology using different host systems like *Escherichia coli*, *Pichia pastoristext*, *Saccharomyces cerevisiae*, *Kluyveromyces lactis*, transgenic animals, and plants [26,27,28,29,30,31]. Bovine serum albumin (BSA) is an acidic, non-glycosylated, 66 kDa protein [32] with a ca. 76% similarity in chemistry composition, space structure, and biological functions to human albumin [33,34]. BSA is a standard protein suitable for partition and interaction studies due to its well-characterized structure, physicochemical properties, and availability at high purity, stability, and solubility [35,36,37,38].

The downstream processing of biopharmaceuticals involves eliminating process- and product-related impurities, along with other contaminants [39], to improve the purity and increase the concentration of target molecules. Despite some of the limitations, such as the high cost derived from resin’s low stability [40] and long cycle time [41], packed-bed chromatography is still the workhorse of most purification processes for protein biopharmaceuticals [42] as it achieves excellent yield and purity [43]. It was implemented for the purification of IFN-α2b through ion exchange and size exclusion chromatography [44,45], immobilized metal-ion affinity chromatography, and reverse-phase HPLC [46]. To overcome some of the packed-bed chromatography limitations, alternative non-chromatographic downstream processes were investigated, namely precipitation, crystallization [47], membrane-based processes [48], magnetic fishing [49], and aqueous biphasic systems [50,51]. The optimization of an efficient and cost-effective purification process is a crucial part of IFN production [52].

Aqueous biphasic systems (ABSs), also known as aqueous two-phase systems (ATPSs), are liquid–liquid systems prepared by mixing at least two different water-soluble compounds in an aqueous medium, such as polymers, salts, alcohols, carbohydrates, or ionic liquids, which form two liquid phases coexisting in equilibrium above certain concentrations, and can be used in liquid–liquid extraction processes [53,54]. Many ABS have exhibited numerous advantages in biotechnological applications [51,55]. Some of the advantages displayed by ABS include their biocompatibility, mostly due to the presence of high water content in both phases, which can provide a biocompatible and non-denaturing environment for cells, proteins, and other biomolecules. Other advantages include scaling up feasibility, the ease of continuous process, and lower interfacial tension [38,41,56]. Moreover, the investment cost of ABS is low and ABS presents low environmental toxicity risks compared to traditional IFN purification methods [57,58]. Further, the ABS can be integrated into various conventional downstream processes (e.g., clarification, concentration, and partial purification) as a single-step operation [59]. ABSs were applied in the separation, recovery, and purification of cells, membrane viruses, proteins, enzymes, nucleic acids, DNA, and other added-value biomolecules [53,54,60]. A common drawback associated with polymer/salt, polymer/polymer, and alcohol/salt ABSs is that they exhibit a small range of polarities between the two phases [53]. To overcome this limitation and improve the protein purification performance of ABSs, several strategies were implemented, for example, the functionalization of PEG with glutaric acid [50,61], and the use of ILs as adjuvants in polymer/salt ABSs [54,62]. In IFN-α2b purification, the use of NaCl as an adjuvant in polymer/salt and alcohol/salt ABSs [63,64] and ILs as adjuvants in polymer/polymer ATPS [65] were reported. Castro et al. investigated the application of ionic liquids (ILs) as adjuvants (at 5 %wt) in polymer/polymer ABS (PEG/PPG) to purify the recombinant protein IFN-α2b from *E. coli* lysates. The purity of IFN-α2b was maximized using ILs composed of aromatic cations and anions with high hydrogen-bond basicity.

Ionic liquids are salts that, in contrast to common electrolytes, are liquid at low temperatures as a result of the delocalized electrical charge distribution and frequent asymmetry of their ions, which prevent crystallization. Further, the physical and chemical properties of ILs can be properly manipulated for specific applications by pre-selecting diverse combinations of cations and anions. This characteristic is transferable to aqueous solutions of ILs, where ABSs composed of ILs have shown vast applicability through adequate control of their phase polarities and affinities [53,66,67]. This aspect is indeed a major benefit of an ionic liquid-based ABS given the difficulty in overcoming the limited polarity range of an alcohol- or polymer-based ABS. Accordingly, in the last decades, a large amount of work has been devoted to ionic liquid-based ABS and the use of ILs as additives or as replacements for common salts in alcohol- and polymer-based ABS. By virtue of their tunability, ionic liquids can ‘‘ideally’’ cover the full hydrophilicity–hydrophobicity range, and specific (and effective) extractions can be directly envisaged. To date, ABSs composed of PEG/salt [63], alcohol/salt [64], and PEG/PPG with ILs as adjuvants (at 5 %wt) [65] were investigated for the purification of IFN-α2b. PEG/salt ABS was also implemented in the purification of IFN alpha-1 [68]. Although, to the best of our knowledge, IL-based ABSs were never attempted for the purification of IFN-α2b or any other human IFN types. Either, polymer- or IL-based ABSs were used for the extraction of BSA from biological fluids [38,69], and BSA partition behaviors were also studied in PEG/dextran [70], PEG/potassium citrate [38], and imidazolium-based IL [69] ABSs. When downstream processes are considered, the toxicity of the substances involved must be pondered alongside other important issues, such as efficiency and biocompatibility toward protein stability. These issues can be addressed by the functionalization of ILs, as in the case of task-specific fluorinated ionic liquids (FILs) developed in our group. These novel FILs, based on perfluoroalkyl sulfonate anions, show total miscibility in water, forming distinct aggregated structures (from spherical to lamellar micelles), depending on their total concentration in the aqueous solution [71]. Also, these FILs reduce the impact of the addition of water upon the ionic liquid’s H-bond acceptance ability, which is a key factor in obtaining functionalized materials to be used, for example, in extraction processes or the dissolution of biomolecules [67]. Additionally, they were shown to be biocompatible and non-toxic in four human cell lines (Caco-2, HepG2, EA.hy926, and HaCaT) and aquatic species with different levels of biological organization (Vibrio fischeri, Daphnia magna, and Lemna minor) [72,73]. Further, the effect of these biocompatible FILs using lysozyme, BSA, human phenylalanine hydroxylase, and IFN-α2b as model proteins [37,74,75,76,77] was evaluated, showcasing these FILs as promising potential biomaterials for drug delivery systems using therapeutic proteins. Finally, the functionalization of ABS was attained through the development of biocompatible ABS based on these perfluoroalkyl sulfonate FILs for the selective partition of lysozyme as the model protein [78]. The results demonstrate that FIL-based ABSs are more versatile and amenable to being tuned, allowing the replacement of high-charge-density salts with more benign phase-forming components, such as low molecular weight carbohydrates (glucose and sucrose) or choline dihydrogen phosphate ([N${}_{1112(\mathrm{OH})}$][H${}_{2}$PO${}_{4}$]), allowing for the selection of the enriched phase.

In this work, IL-based ABS variables, such as the functionalization of ILs, FILs vs. mere fluoro-containing ILs, more benign phase-forming components like sucrose or [N${}_{1112(\mathrm{OH})}$][H${}_{2}$PO${}_{4}$] (known globular protein stabilizers), pH, volume ratio, phase composition (%wt) and phase water content (%wt) were investigated to find out the optimal conditions for simultaneous purification of IFN-α2b and BSA in a single step. Herein, we demonstrate the simultaneous purification of IFN-α2b and BSA to opposite phases of a biphasic point (BP) with high yield (extraction efficiency, %EE) and purity. Purified IFN-α2b and BSA were then characterized by circular dichroism (CD) spectroscopy, differential scanning calorimetry (DSC), and SDS-polyacrylamide gel electrophoresis (SDS-PAGE). Binding studies of IFN-α2b and BSA with ABS phase-forming components were assessed by microscale thermophoresis (MST) to shed light on the interaction ruling partition.

## 2. Results and Discussion

### 2.1. Functionalized Aqueous Biphasic Systems

The development of functionalized, bioprivileged, task-specific FILs was previously achieved through the increasing of the perfluoroalkyl sulfonate anion chain ([C${}_{4}$F${}_{9}$SO${}_{3}$]${}^{-}$ or greater) [37,67,71,72,73,74,75,76,77]. These FILs have demonstrated distinct transitions associated with the formation of stable self-assembled structures, which might explain its complete miscibility in water [71]. The critical aggregation concentrations (CACs) of both imidazolium and choline-based FILs considered in this study, [C${}_{2}$C${}_{1}$Im][C${}_{4}$F${}_{9}$SO${}_{3}$] and [N${}_{1112(\mathrm{OH})}$][C${}_{4}$F${}_{9}$SO${}_{3}$], were previously determined and are presented in Table S1 in the Supplementary Materials. The hydrogen-bonding ability and polarizability of these FILs in water were also studied using Kamlet–Taft parameters [67], proving that FILs reduce the impact of the addition of water upon the IL’s hydrogen bond accepting ability, which is a key factor in obtaining functionalized materials to be used in extraction processes or the dissolution of biomolecules. The results demonstrate that the rich-aggregation behavior of these FILs stimulates the networking of water aggregates and that the water aggregates expand with the increase in the fluorinated chain since the nonpolar part drives the water molecules to the polar domain of the IL [67]. The FILs, based on the [C${}_{4}$F${}_{9}$SO${}_{3}$]${}^{-}$ anion implemented in this study, demonstrated to be biocompatible and non-toxic in four human cell lines (Caco-2, HepG2, EA.hy926, and HaCaT) and aquatic species with different biological organization levels (*Vibrio fischeri*, *Daphnia magna*, and *Lemna minor*) [72,73]. The effects of these biocompatible FILs using lysozyme, BSA, human phenylalanine hydroxylase, and IFN-α2b as model proteins [37,74,75,76,77] were evaluated, proving the [C${}_{4}$F${}_{9}$SO${}_{3}$]-based FILs as promising potential biomaterials for drug delivery systems using therapeutic proteins. The formation of aqueous biphasic systems using these biocompatible FILs, based on perfluoroalkyl sulfonate anions, was first proposed by one of the authors [54] as an extractive platform, proving its potential for the efficient and selective extraction of food colorants. The results show that these FILs can form ABS when combined with a variety of carbohydrates, including monosaccharides, disaccharides, and polyols, generally known as less effective in inducing phase separation. To attain more versatile and tunable ABS for the liquid–liquid extraction of proteins, we developed functionalized ABS ([CF${}_{3}$SO${}_{3}$]${}^{-}$ vs. [C${}_{4}$F${}_{9}$SO${}_{3}$]${}^{-}$ IL anions) combined with more benign phase-forming components, such as low molecular weight carbohydrates (glucose and sucrose) or choline dihydrogen phosphate ([N${}_{1112(\mathrm{OH})}$][H${}_{2}$PO${}_{4}$]) [78]. The systems based on the FILs ([C${}_{4}$F${}_{9}$SO${}_{3}$]-based ILs) highly increased the ABS formation and extraction efficiency of lysozyme, disclosing systems more amenable to be tuned by the proper choice of phase-forming components, and selecting the enriched phase. Also, protein interaction data showed that changing from mere fluoro-containing ILs ([CF${}_{3}$SO${}_{3}$]-based ILs) to FILs ([C${}_{4}$F${}_{9}$SO${}_{3}$]-based ILs) increases protein–IL interactions and protein stability, helping to maintain protein native conformation.

Here, a selection of eight biphasic points (BPs) from both mere fluoro-containing IL ([C${}_{2}$C${}_{1}$Im][CF${}_{3}$SO${}_{3}$] and [C${}_{4}$C${}_{1}$Im][CF${}_{3}$SO${}_{3}$]) and FIL ([C${}_{2}$C${}_{1}$Im][C${}_{4}$F${}_{9}$SO${}_{3}$] and [N${}_{1112(\mathrm{OH})}$][C${}_{4}$F${}_{9}$SO${}_{3}$])-based systems combined with high-charge-density salt (K${}_{3}$PO${}_{4}$) and globular protein stabilizers (sucrose and [N${}_{1112(\mathrm{OH})}$][H${}_{2}$PO${}_{4}$]), as depicted in Table 1, were employed to reach a single-step simultaneous purification of IFN-α2b and BSA. Ternary phase diagrams, describing the minimum concentration of phase-forming components needed for liquid–liquid demixing in each ABS studied (depicted in Figure S1 in the Supplementary Materials), as well as the phase properties, are detailed in a previous work [78].

### 2.2. ABS Phase-Forming Component Effect on the Structural Properties of IFN Alpha-2b and BSA

#### 2.2.1. Differential Scanning Calorimetry

Differential scanning calorimetry (DSC) measurements were performed with Nano DSC equipment, as detailed in Section 3, and used to determine the melting temperature (T${}_{m}$) and enthalpy (ΔH) of protein unfolding. DSC analyzes changes in the structure and stability of proteins as thermal transitions at the nanoscale. Using DSC to measure heat capacity as a function of the temperature of a well-studied protein such as BSA provides keen insights into folding and unfolding processes, as well as factors affecting stability. Due to the volume and concentration requirements of the DSC technique, and the constraints in available quantities of IFN-α2b the DSC experiments on the ABS phase-forming components’ effect on the structural properties of proteins were attained only for BSA. BSA was used as received, without further purification, size exclusion chromatography was not performed in order to isolate the BSA monomer and ensure its monodispersity, as conducted in a previous work of the authors [37], which is verified in the variance of DSC experiments (BSA dimer), for example, Figure S2 in the Supplementary Materials. A representative normalized DSC curve is depicted in Figure S2 in the Supplementary Materials for 1 mg/mL BSA in water, allowing to determine a protein T${}_{m}$ of 75.59 ± 3.68 °C, in accordance with other reported BSA data despite different conditions [37,79,80].

**Figure 1:**
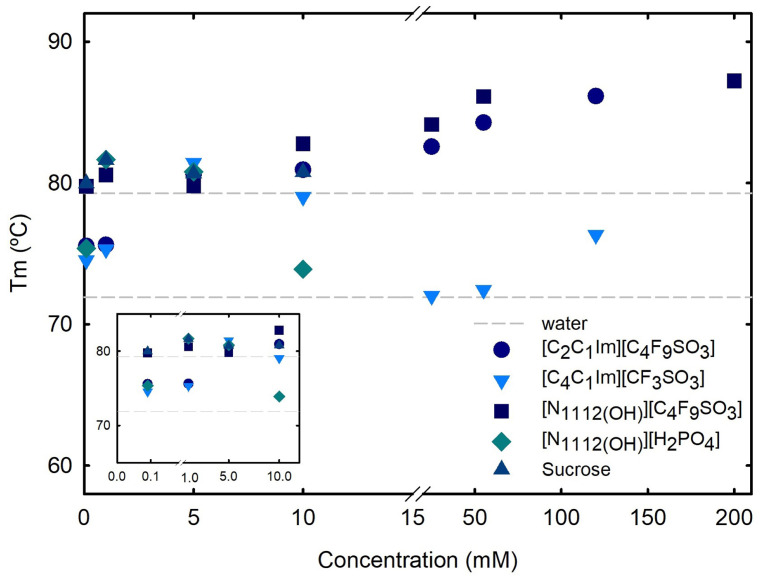
Effect of ABS phase-forming component concentration on DSC BSA T${}_{m}$. Determined T${}_{m}$ in water is plotted as traced line (control), 75.59 ± 3.68 °C. Snapshot in the lower left corner for a concentration range from 0.1 to 10 mM. All BSA T${}_{m}$ data are summarized in Table 2.

The effect of the ABS phase-forming components, FILs ([C${}_{2}$C${}_{1}$Im][C${}_{4}$F${}_{9}$SO${}_{3}$] and [N${}_{1112(\mathrm{OH})}$][C${}_{4}$F${}_{9}$SO${}_{3}$]), mere fluoro containing IL ([C${}_{4}$C${}_{1}$Im][CF${}_{3}$SO${}_{3}$]), [N${}_{1112(\mathrm{OH})}$][H${}_{2}$PO${}_{4}$] and sucrose, on the stability of BSA, is herein discussed. The T${}_{m}$ of BSA, which corresponds to the thermal unfolding transition midpoint, was measured in the absence and presence of the ABS phase-forming components at increasing concentrations (see Figure 1 and Table 2). T${}_{m}$ is the temperature at which both folded and unfolded states of the protein coexist at equilibrium. All analyzed ABS phase-forming components at concentrations of up to 10 mM (concentrations below the first CAC of both studied FILs and compounds have monomer behavior; see Table S1 in the Supplementary Materials) have no impact on BSA T${}_{m}$. The effects of the concentration of FILs ([C${}_{2}$C${}_{1}$Im][C${}_{4}$F${}_{9}$SO${}_{3}$] and [N${}_{1112(\mathrm{OH})}$][C${}_{4}$F${}_{9}$SO${}_{3}$]) on BSA T${}_{m}$, along with the CAC transitions (see Table S1 in the Supplementary Materials) occurring with FILs, were compared to the mere fluoro-containing IL [C${}_{4}$C${}_{1}$Im][CF${}_{3}$SO${}_{3}$] (demixing is not verified for [C${}_{2}$C${}_{1}$Im][CF${}_{3}$SO${}_{3}$] with sucrose [78]; see Figure S1 in the Supplementary Materials for binodal curves and Table 1 for the studied BPs) in the same concentration range, as depicted in Figure 1. BSA T${}_{m}$ values increased significantly in the presence of FILs in all tested conditions, indicating protein stabilization by the FILs. The addition of FILs at the highest concentration (120 mM; above the 4th CAC of [C${}_{2}$C${}_{1}$Im][C${}_{4}$F${}_{9}$SO${}_{3}$], the FIL with a higher CAC number) increased T${}_{m}$ by approximately 11 °C for [C${}_{2}$C${}_{1}$Im][C${}_{4}$F${}_{9}$SO${}_{3}$], and 12 °C for [N${}_{1112(\mathrm{OH})}$][C${}_{4}$F${}_{9}$SO${}_{3}$]. For mere fluoro-containing IL [C${}_{4}$C${}_{1}$Im][CF${}_{3}$SO${}_{3}$], [N${}_{1112(\mathrm{OH})}$][H${}_{2}$PO${}_{4}$] and sucrose, BSA T${}_{m}$ is maintained. These results corroborate and expand upon the previous results attained by the authors [37], where the results obtained from ITC and conductivity supported the conclusion that BSA not only interacts with [C${}_{2}$C${}_{1}$Im][C${}_{4}$F${}_{9}$SO${}_{3}$], but is encapsulated by the FIL, while its stability is improved.

To better understand the binding affinity of the BSA-FIL complexes, Table 2 compares the calorimetry enthalpy variation to the van’t Hoff enthalpy variation, as well as the system’s Gibbs energy on the dependency of thermal transitions on FIL concentration. The values close to 1 presented in Table 2 for the ratio between calorimetric and van’t Hoff enthalpies provide a clear view of how well-suited the fit method is to our data (for all the studied ABS phase-forming components) [81]. The sign and magnitude of ΔH can, respectively, provide insights into the nature and strength of the interactions between protein and ABS phase-forming components. A positive ΔH in all the gathered data suggests that the binding involves endothermic processes, such as the breaking of hydrogen bonds or the release of water molecules from the protein surface. A large positive ΔH indicates strong, favorable interactions, suggesting that the binding involves energy-releasing processes such as hydrogen bonding or favorable hydrophobic interactions. A positive ΔS indicates that the binding process includes an increase in disorder, such as the release of structured water or conformational changes that result in enhanced flexibility. A high positive ΔS value indicates that the binding process increases molecular flexibility and conformational entropy. This implies that the binding involves hydrophobic interactions or the release of structured water molecules, resulting in increased disorder. By combining the information from ΔH and ΔS, it is possible to deduce the interactions primarily involved in the BSA-FIL binding. A large positive ΔH combined with a positive ΔS indicates that hydrophobic interactions may be prevalent where nonpolar regions of BSA interact with the FIL hydrophobic moieties.

#### 2.2.2. Circular Dichroism Spectroscopy

**Figure 2:**
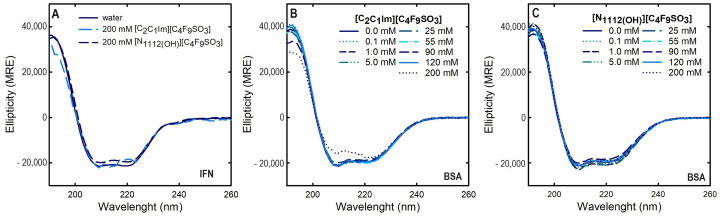
CD spectra of (**A**) IFN-α2b 0.125 mg/mL in water, 200 mM [C${}_{2}$C${}_{1}$Im][C${}_{4}$F${}_{9}$SO${}_{3}$] aqueous solution, and 200 mM [N${}_{1112(\mathrm{OH})}$][C${}_{4}$F${}_{9}$SO${}_{3}$] aqueous solution. CD spectra of BSA 1.0 mg/mL in water, (**B**) 0.1–200 mM [C${}_{2}$C${}_{1}$Im][C${}_{4}$F${}_{9}$SO${}_{3}$] aqueous solution, and (**C**) 0.1–200 mM [N${}_{1112(\mathrm{OH})}$][C${}_{4}$F${}_{9}$SO${}_{3}$] aqueous solution. All spectra were acquired at 25 ${}^{\circ }$C.

Circular dichroism (CD) is widely used to study the secondary structures of proteins, peptides and nucleic acids, because it requires a low concentration sample, is a nondestructive method, allows studying a wide range of variables (temperature, pH, etc.), and provides information on the effect of added ligands on protein folding and interactions. Far-UV (190–260 nm) CD spectroscopy was used to assess the IFN-α2b and BSA secondary structure since the spectra are characteristic of different structural elements. The spectrum obtained at 25 °C for both proteins in water, 0.125 mg/mL IFN-α2b and 1 mg/mL BSA, is presented in Figure 2. The effect of the FILs [C${}_{2}$C${}_{1}$Im][C${}_{4}$F${}_{9}$SO${}_{3}$] and [N${}_{1112(\mathrm{OH})}$][C${}_{4}$F${}_{9}$SO${}_{3}$] concentration on both IFN-α2b and BSA CD spectra is also depicted in Figure 2. Figure S3 in the Supplementary Materials illustrates the effect of the other studied ABS phase-forming components ([C${}_{4}$C${}_{1}$Im][CF${}_{3}$SO${}_{3}$], [N${}_{1112(\mathrm{OH})}$][H${}_{2}$PO${}_{4}$], and sucrose) on BSA CD spectra. The relative content of the proteins of all secondary structural features (regular and distorted α-helix and β-sheet, turns, and unordered or random coils) are predicted using the CONTIN-LL deconvolution algorithm [82] with the SMP180 reference set for both IFN-α2b and BSA in water, and the presence of ABS phase-forming components, as displayed in Table 3.

IFN-α2b and BSA are predominantly α-helical proteins, and the far-UV CD spectrum of both native proteins (Figure 2) shows minima at around 208 nm and 222 nm (209 nm and 222 nm IFN-α2b, and 209 nm and 221 nm BSA), characteristic of α-helical proteins [83]. The predicted IFN-α2b and BSA α-helical contents of 0.44 and 0.61, respectively, are also in good agreement with previous reports [37,84,85]. Upon the FIL ([C${}_{2}$C${}_{1}$Im][C${}_{4}$F${}_{9}$SO${}_{3}$] and [N${}_{1112(\mathrm{OH})}$][C${}_{4}$F${}_{9}$SO${}_{3}$]) addition, both IFN-α2b and BSA maintain their characteristic spectra (Figure 2). Further, the relative α-helical and β-sheet contents of IFN-α2b increase in the presence of both FILs, while turns and unordered or random coils decrease, indicating that IFN-α2b is stabilized in the presence of [C${}_{2}$C${}_{1}$Im][C${}_{4}$F${}_{9}$SO${}_{3}$] and [N${}_{1112(\mathrm{OH})}$][C${}_{4}$F${}_{9}$SO${}_{3}$] at a concentration of 200 mM. For BSA, the relative α-helical, β-sheet, and turns contents increase in the presence of both FILs, while unordered or random coil decreases, indicating that BSA is stabilized in the presence of [C${}_{2}$C${}_{1}$Im][C${}_{4}$F${}_{9}$SO${}_{3}$] and [N${}_{1112(\mathrm{OH})}$][C${}_{4}$F${}_{9}$SO${}_{3}$] at concentrations of up to 200 mM (up to 120 mM for α-helix with [C${}_{2}$C${}_{1}$Im][C${}_{4}$F${}_{9}$SO${}_{3}$]; at 200 mM, a slight decrease is verified). The characteristic CD spectrum of BSA is also maintained upon the addition of the mere fluoro-containing IL ([C${}_{4}$C${}_{1}$Im][CF${}_{3}$SO${}_{3}$]), [N${}_{1112(\mathrm{OH})}$][H${}_{2}$PO${}_{4}$], and sucrose (Figure S3 in the Supplementary Materials). A more subtle impact on the BSA secondary structure content, including a slight increase in α-helical, β-sheet, and turns contents, and a slight in the unordered or random coil content, is verified with these ABS phase-forming components.

**Figure 3:**
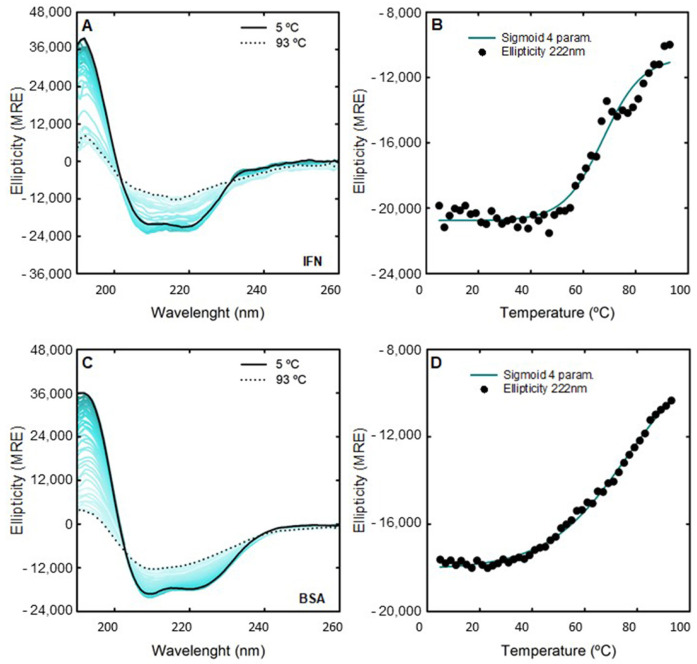
Spectra of (**A**) IFN-α2b and (**C**) BSA in water collected as a function of temperature from 5 °C (full black) to 93 °C (dotted black). Ellipticity values for each temperature curve at 222 nm (symbols) and fitting with a sigmoid with four parameters for (**B**) IFN-α2b and (**D**) BSA.

**Figure 4:**
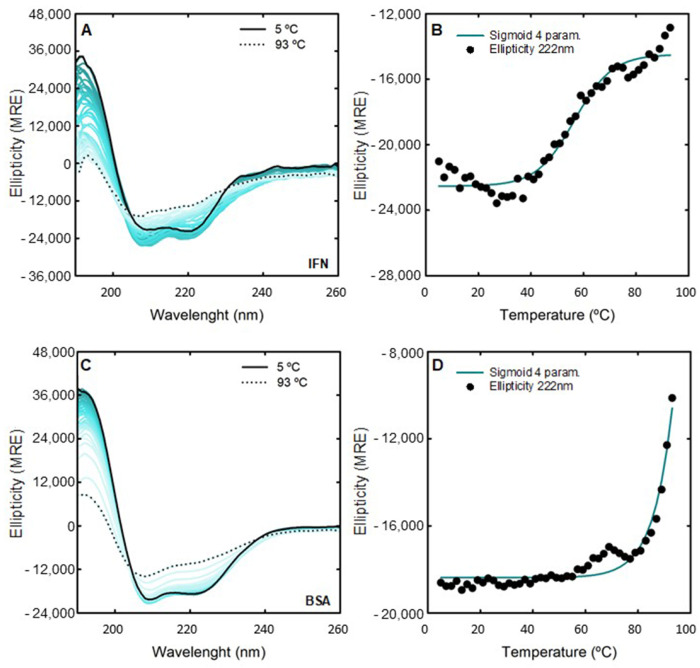
Spectra of (**A**) IFN-α2b and (**C**) BSA in 200 mM [N${}_{1112(\mathrm{OH})}$][C${}_{4}$F${}_{9}$SO${}_{3}$] collected as a function of temperature from 5 °C (full black) to 93 °C (dotted black). Ellipticity values for each temperature curve at 222 nm (symbols) and fitting with a sigmoid with four parameters for (**B**) IFN-α2b and (**D**) BSA.

The CD thermal stability of IFN-α2b (0.125 mg/mL) and BSA (1.0 mg/mL) for all discussed ABS phase-forming components at 200 mM were studied. The unfolding of the secondary structure was monitored using far-UV CD at 222 nm, 208 nm, and 192 nm (the most relevant wavelengths for α-helical proteins, like IFN-α2b and BSA) with an increase in temperature from 5 °C to 93 °C. Figure 3 shows the changes in the far-UV CD spectrum of the native IFN-α2b and BSA from 5 °C to 93 °C, and the monitored thermal unfolding curves at 222 nm. The same analyses attained for 208 nm and 192 nm are illustrated in Figures S5 and S6 in the Supplementary Materials. The corresponding T${}_{m}$ values are estimated by fitting to a sigmoid with four parameters (one-way ANOVA, significance level of 0.05) and summarized in Table S2 in the Supplementary Materials. The CD T${}_{m}$ of 0.125 mg/mL native IFN-α2b is in good agreement with what was previously reported [86]. Also, the CD T${}_{m}$ of the 1.0 mg/mL native BSA is in good agreement with the DSC results. The changes in the far-UV CD spectra of IFN-α2b and BSA with 200 mM [N${}_{1112(\mathrm{OH})}$][C${}_{4}$F${}_{9}$SO${}_{3}$] and [C${}_{2}$C${}_{1}$Im][C${}_{4}$F${}_{9}$SO${}_{3}$] with an increase in temperature, as well as the monitored thermal unfolding curves at 222 nm, are illustrated in Figure 4 and Figure S4 in the Supplementary Materials, respectively. The estimated T${}_{m}$ values are summarized in Table S2 in the Supplementary Materials. The same analysis is attained for 208 nm and 192 nm, as depicted in Figures S5 and S6 in the Supplementary Materials. All the CD T${}_{m}$ results are summarized in Table S2 in the Supplementary Materials. The thermal stability assessed by far-UV CD is solely determined through the thermal decay of the alpha-helical structure. Globally, the CD studies show the success of [N${}_{1112(\mathrm{OH})}$][C${}_{4}$F${}_{9}$SO${}_{3}$] and [C${}_{2}$C${}_{1}$Im][C${}_{4}$F${}_{9}$SO${}_{3}$] in preserving the α-helical content of IFN-α2b and BSA against thermal stress. Upon FIL addition, the IFN-α2b T${}_{m}$ slightly decreases, and the BSA T${}_{m}$ increases, which is in good agreement with the DSC results. Additionally, the changes in the far-UV CD spectra of BSA with 200 mM [C${}_{2}$C${}_{1}$Im][C${}_{4}$F${}_{9}$SO${}_{3}$], [N${}_{1112(\mathrm{OH})}$][H${}_{2}$PO${}_{4}$] and sucrose with an increase in temperature, as well as the monitored thermal unfolding curves at 222 nm, 208 nm, and 192 nm, are illustrated in Figure S7 in the Supplementary Materials. The estimated BSA T${}_{m}$ values are summarized in Table S2 in the Supplementary Materials. The results show that the BSA T${}_{m}$ is maintained upon the addition of mere fluoro-containing IL [C${}_{4}$C${}_{1}$Im][CF${}_{3}$SO${}_{3}$], [N${}_{1112(\mathrm{OH})}$][H${}_{2}$PO${}_{4}$] and sucrose, in good agreement with the DSC results.

### 2.3. IFN Alpha-2b and BSA Partition in Functionalized ABS

#### 2.3.1. Extraction Efficiency and Purification Factor

In the biopharmaceutical industry, it is common to have multiple valuable components present in a single feedstock, such as IFN-α2b and serum albumin. In the preparation of inclusion-body IFN-α2b from *E. coli*, other proteins derived from unbroken cells (most likely) or *E. coli* cytoplasmatic proteins co-precipitated or trapped during aggregate formation [15] are also present, such as serum albumin [14]. Several hosts have been applied to produce all classes of interferon molecules, such as *Escherichia coli*, *Bacillus subtilis*, *Lactococcus lactistext*, *Pichia pastoristext*, *Saccharomyces cerevisiae*, *Trichoderma reesei*, *Yarrowia lipolytica*, insect cells, plants, transgenic mice, and mammalian cells [6,87]. For example, the in vivo efficacy of IFN beta increases by its natural glycosylation [88], and since mammalian cell lines are the best hosts for obtaining recombinant proteins with native glycosylation patterns, they represent the best compromise between yield and quality. The concentration of interferon in the culture supernatants of mammalian cells can range from a few pg/mL to several μg/mL, and the concentration of albumin can also vary widely, ranging from a few μg/mL to several mg/mL. Herein, all partition studies were performed in this concentration range (0.045 mg/mL IFN-α2b and 1.0 mg/mL BSA) to stress the robustness of the proposed functionalized ABS to reach a single-step simultaneous purification of IFN-α2b and BSA (IFN-α2b the high-value-added compound).

The influence of eight biphasic systems comprising an ionic liquid-based bottom-phase, FIL ([C${}_{2}$C${}_{1}$Im][C${}_{4}$F${}_{9}$SO${}_{3}$] and [N${}_{1112(\mathrm{OH})}$][C${}_{4}$F${}_{9}$SO${}_{3}$]) vs. mere fluoro-containing IL ([C${}_{2}$C${}_{1}$Im][CF${}_{3}$SO${}_{3}$] and [C${}_{4}$C${}_{1}$Im][CF${}_{3}$SO${}_{3}$]), and non-ionic liquid-based top-phase (K${}_{3}$PO${}_{4}$, [N${}_{1112(\mathrm{OH})}$][H${}_{2}$PO${}_{4}$], and sucrose) on IFN-α2b and BSA simultaneous purification were studied. The first step is the individual partition of both proteins in the eight systems depicted in Table 1. The phase diagrams of the selected systems are depicted in Figure S1 in the Supplementary Materials, and are detailed in a previous work [78]. Table 1 summarizes the volume ratio, pH, and composition of both the ionic liquid phase and non-ionic liquid phase of the selected systems. IFN-α2b and BSA partition behavior in each BP is characterized in terms of extraction efficiency (%EE, Equation (1)) and the IFN-α2b purification factor ($P_{F\;1}$ and $P_{F\;2}$). The ionic liquid-based bottom-phase purification factor ($P_{F\;1}$) is the ratio of the specific IFN-α2b concentration in the FIL ([C${}_{2}$C${}_{1}$Im][C${}_{4}$F${}_{9}$SO${}_{3}$] and [N${}_{1112(\mathrm{OH})}$][C${}_{4}$F${}_{9}$SO${}_{3}$]) or mere fluoro-containing IL ([C${}_{4}$C${}_{1}$Im][CF${}_{3}$SO${}_{3}$])-rich phase of ABS to the specific IFN-α2b concentration in the biphasic point (the initial solubilized IFN-α2b and BSA from individual partition). The $P_{F\;1}$ was calculated according to Equation (2), allowing to evaluate the purity of IFN-α2b in the bottom phase and assess the efficiency of FILs over mere fluoro-containing ILs. Additionally, the non-ionic liquid-based top-phase purification factor ($P_{F\;2}$) was calculated according to Equation (3) to assess the purity of BSA in the top phase (BSA is 22-fold more concentrated than IFN-α2b in the initial solubilized sample). $P_{F\;2}$ is the ratio of the specific IFN-α2b concentration in the non-IL-rich phase of ABS to the specific IFN-α2b concentration in the biphasic point (the initial solubilized IFN-α2b and BSA from the individual partition). The results of %EE of IFN-α2b and BSA, as well as $P_{F\;1}$ and $P_{F\;2}$ of IFN-α2b are shown in Table 4. The precise quantification of IFN-α2b and BSA was determined through the MICRO BCA protein assay (Section 3), which is suitable for diluted protein samples, and allows working with higher dilution factors of the ABS phase samples, avoiding any interference of the ABS phase-forming components on the quantification. Additionally, the quantification of BSA was determined through Bradford and BCA protein assays (Table S3 in the Supplementary Materials), in good agreement with the MICRO BCA results. Further, SDS-PAGE was implemented for accurate characterization of the protein composition within each phase of the studied ABS for simultaneous partitions of IFN-α2b and BSA (Section 2.3.3).

**Figure 5:**
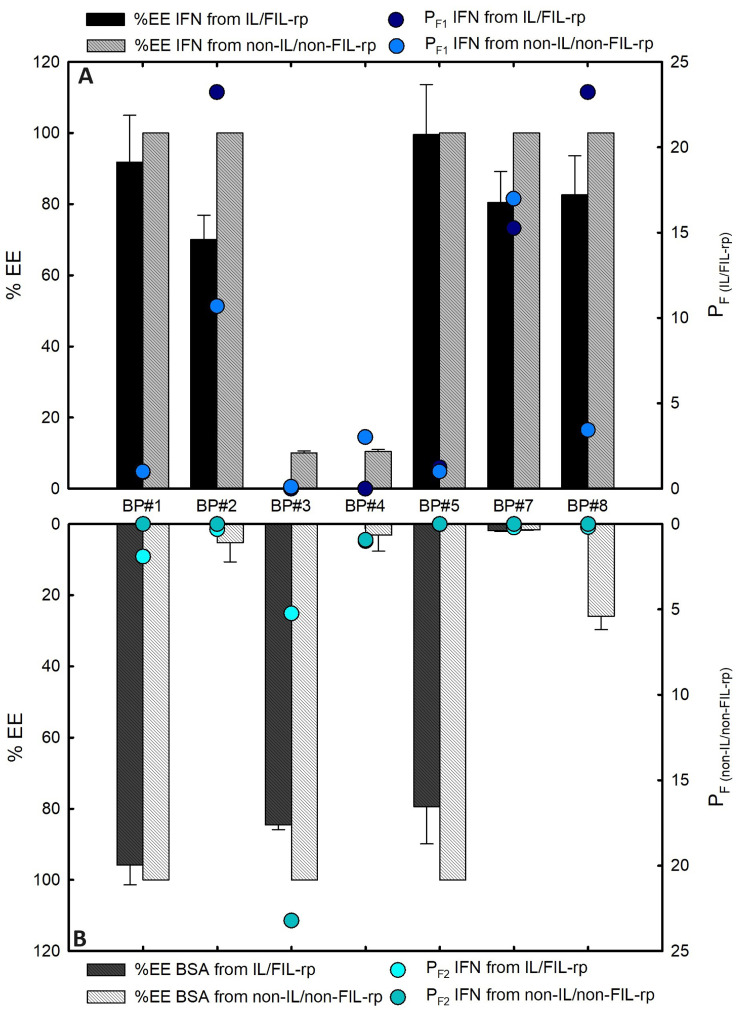
(**A**) IFN-α2b and (**B**) BSA extraction efficiencies (%EE; Equation (1)) and purification factor (P${}_{F\;1}$ and P${}_{F\;2}$; Equations (2) and (3) for the biphasic points studied, BP#1–BP#8. All %EE, P${}_{F\;1}$ and P${}_{F\;2}$ values are summarized in Table 4.

The IFN-α2b and BSA extraction efficiency (%EE) and IFN-α2b purification factor ($P_{F\;1}$ and $P_{F\;2}$) are depicted in Figure 5. From these individual partition results, one can suggest that the systems 30 %wt [C${}_{2}$C${}_{1}$Im][C${}_{4}$F${}_{9}$SO${}_{3}$] + 2 %wt K${}_{3}$PO${}_{4}$ (BP#2), 30 %wt [C${}_{2}$C${}_{1}$Im][C${}_{4}$F${}_{9}$SO${}_{3}$] + 20 %wt [N${}_{1112(\mathrm{OH})}$][H${}_{2}$PO${}_{4}$] (BP#7), 30 %wt [N${}_{1112(\mathrm{OH})}$][C${}_{4}$F${}_{9}$SO${}_{3}$] + 30 %wt [N${}_{1112(\mathrm{OH})}$][H${}_{2}$PO${}_{4}$] (BP#8), and 30 %wt [C${}_{4}$C${}_{1}$Im][CF${}_{3}$SO${}_{3}$] + 25 %wt sucrose (BP#3) allow a single-step simultaneous purification of IFN-α2b and BSA. IFN-α2b is a single, non-glycosylated polypeptide chain with a molecular weight of 19 kDa [7,8] and dimensions of 40 Å × 60 Å × 20 Å [89], more compact than the acidic, non-glycosylated, 66 kDa multi-domain globular BSA protein, with approximate dimensions of 140 Å × 40 Å × 40 Å [32]. The partitioning behavior of IFN-α2b and BSA can be influenced by the pH of each phase of the ABS since both IFN-α2b and BSA are charged molecules [90]. The iso-electric points of IFN-α2b and BSA are around 5.9 [44] and 4.7 [91], respectively; therefore, both proteins are negatively charged at a neutral pH, and positively charged under acidic conditions. Information on the solvent-accessible surface area (SASA) was not found in the literature for IFN-α2b; however, information regarding another type I interferon family, IFN-α2a, which only differs in one amino acid residue from IFN-α2b, has an SASA of approximately 10,000 Å${}^{2}$, and the contact area is more hydrophobic than hydrophilic [92,93]. BSA, well-known for its conformational adaptability, has an SASA of approximately 30,000 Å${}^{2}$ [94], and the surface is more hydrophilic than hydrophobic [95].

In K${}_{3}$PO${}_{4}$-based systems, both phases are at higher pH values, both proteins are negatively charged, the IFN-α2b-enriched phase is the ionic liquid-rich phase for BP#1 and BP#2 (Table 4), and BSA partitions to the ionic liquid-rich phase in the system containing mere fluoro-containing IL (BP#1) and to the K${}_{3}$PO${}_{4}$-rich phase in the FIL-based system (BP#2). In conventional inorganic salt-based ABSs at higher pH values (well above neutral pH), most proteins become negatively charged due to the high pH value, and the negatively charged proteins tend to partition to the non-inorganic salt phase and be repelled from the inorganic salt-rich phases [68]. Our results demonstrate that the functionalized FIL-based ABS, even in combination with high-charge-density salts, allows for a more amenable ABS (lower quantities of K${}_{3}$PO${}_{4}$ for demixing; see Figure S1 in the Supplementary Materials) with a selection of the enriched phase; BSA is not repelled from the inorganic salt-rich phase. For the sucrose-based system combined with [C${}_{4}$C${}_{1}$Im][CF${}_{3}$SO${}_{3}$] (BP#3), the pHs of both phases (5.00 and 5.25) are near the isoelectric points of both IFN-α2b and BSA, which should decrease the extent of or eliminate the electrostatic interactions between IFN-α2b or BSA and the ABS phase-forming components. The same is verified for IFN-α2b in the sucrose-based system combined with [C${}_{2}$C${}_{1}$Im][C${}_{4}$F${}_{9}$SO${}_{3}$] (BP#4), where the pHs of both phases (6.75 and 6.75) are near the isoelectric point of IFN-α2b. At these pH values, BSA is negatively charged. For the system with mere fluoro-containing IL ([C${}_{4}$C${}_{1}$Im][CF${}_{3}$SO${}_{3}$]; BP#3), the IFN-α2b-enriched phase is the sucrose-rich phase, and the BSA-enriched phase is the IL-rich phase. For the FIL-based system ([C${}_{2}$C${}_{1}$Im][C${}_{4}$F${}_{9}$SO${}_{3}$]; BP#4), both proteins partition to the sucrose-rich phase. IFN-α2b is positively charged in all FIL-based ABSs combined with [N${}_{1112(\mathrm{OH})}$][H${}_{2}$PO${}_{4}$] (BP#5, BP#6, BP#7, and BP#8), and for all systems, the IFN-α2b-enriched phase is the FIL-rich phase ([C${}_{2}$C${}_{1}$Im][C${}_{4}$F${}_{9}$SO${}_{3}$] or [N${}_{1112(\mathrm{OH})}$][C${}_{4}$F${}_{9}$SO${}_{3}$]). The total BSA charge depends on the [N${}_{1112(\mathrm{OH})}$][H${}_{2}$PO${}_{4}$] concentration, up to 10 wt% [N${}_{1112(\mathrm{OH})}$][H${}_{2}$PO${}_{4}$] (BP#5 and BP#6), BSA is positively charged. For higher concentrations, 20 %wt [N${}_{1112(\mathrm{OH})}$][H${}_{2}$PO${}_{4}$] (BP#7) and 30 %wt [N${}_{1112(\mathrm{OH})}$][H${}_{2}$PO${}_{4}$] (BP#8), the pHs of both phases are near the isoelectric point of BSA. Up to 10 %wt of [N${}_{1112(\mathrm{OH})}$][H${}_{2}$PO${}_{4}$] in the BSA-enriched phase is the FIL-rich phase (BP#5 and BP#6), and for higher [N${}_{1112(\mathrm{OH})}$][H${}_{2}$PO${}_{4}$] concentrations (20 %wt, BP#7; 30 %wt, BP#8) BSA partitions to the [N${}_{1112(\mathrm{OH})}$][H${}_{2}$PO${}_{4}$]-rich phase (the partition results for BP#6 are similar to BP#5). IFN-α2b is more hydrophobic than BSA, and at the pH of the FIL-based ABS combined with [N${}_{1112(\mathrm{OH})}$][H${}_{2}$PO${}_{4}$] IFN-α2b is positively charged. These two features advantageously promote the interaction with the anionic counterparts of the FILs, which also have a strong hydrophobic nature.

The partition behavior of lysozyme (Lys) in the ABS systems listed in Table 1 was assessed by the authors in a previous work [78]. Lys, a globular glycoprotein with a molecular weight of 14 kDa and approximate dimensions of 27.8 Å × 11.8 Å × 11.8 Å [96], has an iso-electric point of approximately 11.0 [96] and an SASA of circa 7000 Å${}^{2}$[94]. For example, globular proteins like BSA and Lys can show different interaction behaviors in the presence of monovalent and divalent ions as their pI values are different [97]. Similarly, the partition behaviors of BSA and Lys in the studied ABS have some differences (see the BSA partition behavior in Table 4). In [N${}_{1112(\mathrm{OH})}$][H${}_{2}$PO${}_{4}$]-based systems (BP#5, BP#6, BP#7, and BP#8), Lys is positively charged in all systems, the BSA charge depends on the [N${}_{1112(\mathrm{OH})}$][H${}_{2}$PO${}_{4}$] concentration (viz. the above discussion), and the partition behavior of both proteins is the same. Up to 10 %wt of [N${}_{1112(\mathrm{OH})}$][H${}_{2}$PO${}_{4}$] in the Lys-enriched phase is the FIL-rich phase (BP#5 and BP#6), and for higher [N${}_{1112(\mathrm{OH})}$][H${}_{2}$PO${}_{4}$] concentrations (20 %wt, BP#7; 30 %wt, BP#8), Lys partitions to the [N${}_{1112(\mathrm{OH})}$][H${}_{2}$PO${}_{4}$]-rich phase. For the systems with sucrose and K${}_{3}$PO${}_{4}$, the partition behavior of BSA and Lys is distinct. In the sucrose-based systems, Lys is positively charged in both systems, in opposition to BSA, which is near the iso-electric point in BP#3 and negatively charged in BP#4; the Lys-enriched phase is the sucrose-rich phase for the system with mere fluoro-containing IL (BP#3) and the FIL-rich phase for the system with [C${}_{2}$C${}_{1}$Im][C${}_{4}$F${}_{9}$SO${}_{3}$]. In K${}_{3}$PO${}_{4}$-based systems, both phases are at higher pH values, both BSA and Lys are negatively charged, and the Lys-enriched phase is an ionic liquid-rich phase ([C${}_{2}$C${}_{1}$Im][CF${}_{3}$SO${}_{3}$] and [C${}_{2}$C${}_{1}$Im][C${}_{4}$F${}_{9}$SO${}_{3}$]).

IFN-α2b and BSA %EE vs. the pH of the IL- or FIL-rich phase of the studied systems detailed above are depicted in Figure S8 in the Supplementary Materials. The impact of other relevant phase properties of the eight biphasic systems, comprising the ionic liquid-based bottom-phase (FIL, ([C${}_{2}$C${}_{1}$Im][C${}_{4}$F${}_{9}$SO${}_{3}$] and [N${}_{1112(\mathrm{OH})}$][C${}_{4}$F${}_{9}$SO${}_{3}$]), or mere fluoro-containing IL, [C${}_{4}$C${}_{1}$Im][CF${}_{3}$SO${}_{3}$]) and non-ionic liquid-based top-phase (K${}_{3}$PO${}_{4}$ [N${}_{1112(\mathrm{OH})}$][H${}_{2}$PO${}_{4}$] and sucrose), on the extraction efficiency (%EE, Equation (1)) of IFN-α2b and BSA, namely, the amount of water (%wt H${}_{2}$O; Figure S9 in the Supplementary Materials), the amount of mere fluoro-containing IL or FIL (%wt IL/FIL; Figure S10 in the Supplementary Materials), the amount of K${}_{3}$PO${}_{4}$, [N${}_{1112(\mathrm{OH})}$][H${}_{2}$PO${}_{4}$], or sucrose (%wt non-IL/FIL; Figure S11 in the Supplementary Materials), and the volume ratio (Figure S12 in the Supplementary Materials), were assessed. No clear trend is observed in IFN-α2b and BSA %EE with phase properties and the volume ratio. The phase properties and volume ratio are detailed in Table 1.

In previous works [37,74,75,77,98], we developed biocompatible perfluoroalkyl sulfonate-based FILs that allow complex formation between the FIL aggregates with IFN-α2b, BSA, and Lys. Further, we developed functionalized ABSs based on these perfluoroalkyl sulfonate FILs for the selective partitions of IFN-α2b and BSA, assessed herein, and the selective partition of Lys in a previous study [78]. The results point to the existence of other properties that determine protein–FIL interactions (as the other ABS phase-forming components), apart from the protein charge or solvent-accessible surface area. Overall, the results demonstrate that FIL-based ABSs are more versatile and tunable, allowing for the replacement of high-charge-density salts with more benign phase-forming components, such as sucrose or [N${}_{1112(\mathrm{OH})}$][H${}_{2}$PO${}_{4}$], and enabling the selection of the enriched phase.

#### 2.3.2. Protein Structure and Stability in ABS Phases

In addition to the characterization of IFN-α2b and BSA partition behavior in ABS, in terms of extraction efficiency (%EE) and the IFN-α2b purification factor ($P_{F\;1}$ and $P_{F\;2}$), it is of relevance that neither protein shows denaturation. The efficacy and safety of therapeutic proteins are related to the folded conformation of the protein, and different manipulations in extraction or purification steps have the potential to alter the active ingredient. DSC is used in different studies to address protein folding and stability [99], as well as CD, an analytical method often used to examine structural changes that occur when a protein interacts with another molecule [37,74,76,77,100] (and references cited therein). Herein, the impact of the ABS phase on the stability and structure of IFN-α2b and BSA was assessed and compared with the behavior of the proteins in water. CD and DSC were used to address the structure and stability of IFN-α2b and BSA, and to infer possible changes induced during the ABS purification step.

**Figure 6:**
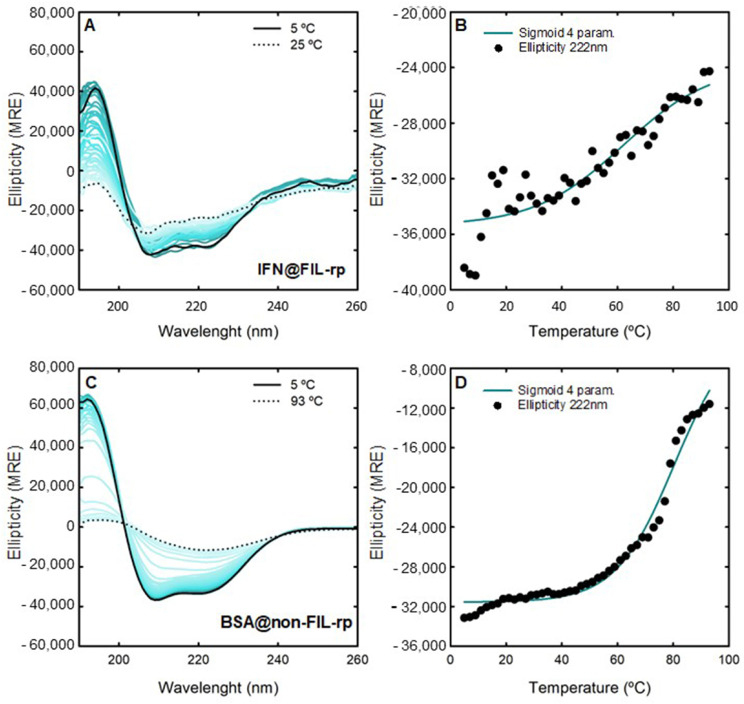
Spectra of (**A**) IFN-α2b and (**C**) BSA in FIL-rp and non-FIL-rp, respectively, of the biphasic point 30 %wt [N${}_{1112(\mathrm{OH})}$][C${}_{4}$F${}_{9}$SO${}_{3}$] + 30 %wt [N${}_{1112(\mathrm{OH})}$][H${}_{2}$PO${}_{4}$] (BP no. 8) collected as a function of temperature from 5 °C (full black) to 93 °C (dotted black). Ellipticity values for each temperature curve at 222 nm (symbols) and fitting a sigmoid with four parameters for (**B**) IFN-α2b and (**D**) BSA.

For FIL-based biphasic systems ([C${}_{2}$C${}_{1}$Im][C${}_{4}$F${}_{9}$SO${}_{3}$] and [N${}_{1112(\mathrm{OH})}$][C${}_{4}$F${}_{9}$SO${}_{3}$]), allowing single-step simultaneous purification of IFN-α2b and BSA (BP#2, BP#7, and BP#8), [C${}_{2}$C${}_{1}$Im][C${}_{4}$F${}_{9}$SO${}_{3}$]-rich phases are not technically feasible for CD; this is due to the high signal intensity of the imidazolium ring and the high concentration of [C${}_{2}$C${}_{1}$Im][C${}_{4}$F${}_{9}$SO${}_{3}$] in FIL-rich phases, ranging from 3007 mM (BP#2) to 11,456 mM (BP#7) [C${}_{2}$C${}_{1}$Im][C${}_{4}$F${}_{9}$SO${}_{3}$] in the [C${}_{2}$C${}_{1}$Im][C${}_{4}$F${}_{9}$SO${}_{3}$]-rich phases. Spectra were only measured for the [N${}_{1112(\mathrm{OH})}$][C${}_{4}$F${}_{9}$SO${}_{3}$]-based system (BP#8). The unfolding of the secondary structures of IFN-α2b and BSA in the enriched phases from their partition in the system, 30 %wt [N${}_{1112(\mathrm{OH})}$][C${}_{4}$F${}_{9}$SO${}_{3}$] + 30 %wt [N${}_{1112(\mathrm{OH})}$][H${}_{2}$PO${}_{4}$] (BP#8), in the [N${}_{1112(\mathrm{OH})}$][C${}_{4}$F${}_{9}$SO${}_{3}$]-rich phase and [N${}_{1112(\mathrm{OH})}$][H${}_{2}$PO${}_{4}$]-rich phase, respectively, was assessed using far-UV CD at 222 nm, 208 nm, and 192 nm, with an increase in temperature from 5 °C to 93 °C. Figure 6 shows the changes in the far-UV CD spectrum of IFN-α2b in the [N${}_{1112(\mathrm{OH})}$][C${}_{4}$F${}_{9}$SO${}_{3}$]-rich phase, BSA in the [N${}_{1112(\mathrm{OH})}$][H${}_{2}$PO${}_{4}$]-rich phase from 5 °C to 93 °C, and the monitored thermal unfolding curves at 222 nm. The same analysis was attained for 208 nm and 192 nm as illustrated in Figure S13 in the Supplementary Materials. The corresponding T${}_{m}$ values are depicted in Table 5, and were estimated by fitting a sigmoid with four parameters (one-way ANOVA, with a significance level of 0.05). Comparing the CD T${}_{m}$ of 0.125 mg/mL IFN-α2b in water with the CD T${}_{m}$ of 0.099-0.12 mg/mL IFN-α2b in the [N${}_{1112(\mathrm{OH})}$][C${}_{4}$F${}_{9}$SO${}_{3}$]-rich phase (partition of 0.045 mg/mL IFN-α2b in BP#8), and the CD T${}_{m}$ of 1.0 mg/mL BSA in water with the CD T${}_{m}$ of 1.2–1.6 mg/mL BSA in the [N${}_{1112(\mathrm{OH})}$][H${}_{2}$PO${}_{4}$]-rich phase (partition of 1.0 mg/mL BSA in BP#8), it can be observed that the IFN-α2b T${}_{m}$ is maintained in the FIL-rich phase (BP#8) and the BSA T${}_{m}$ is increased in the [N${}_{1112(\mathrm{OH})}$][H${}_{2}$PO${}_{4}$]-rich phase (BP#8). The concentrations of [N${}_{1112(\mathrm{OH})}$][C${}_{4}$F${}_{9}$SO${}_{3}$] and [N${}_{1112(\mathrm{OH})}$][H${}_{2}$PO${}_{4}$] in both phases, the FIL-rich phase and [N${}_{1112(\mathrm{OH})}$][H${}_{2}$PO${}_{4}$]-rich phase of the biphasic BP#8 system, are far higher than the 200 mM (highest CAC of the studied FILs, viz. Table S1 in the Supplementary Materials) analyzed in Section 2.2, 6237 mM [N${}_{1112(\mathrm{OH})}$][C${}_{4}$F${}_{9}$SO${}_{3}$] and 1990 mM [N${}_{1112(\mathrm{OH})}$][H${}_{2}$PO${}_{4}$] in the [N${}_{1112(\mathrm{OH})}$][C${}_{4}$F${}_{9}$SO${}_{3}$]-rich phase and 415 mM [N${}_{1112(\mathrm{OH})}$][C${}_{4}$F${}_{9}$SO${}_{3}$] and 4758 mM [N${}_{1112(\mathrm{OH})}$][H${}_{2}$PO${}_{4}$] in the [N${}_{1112(\mathrm{OH})}$][H${}_{2}$PO${}_{4}$]-rich phase. The CD studies of both protein-enriched phases of a FIL-based ABS show the success of the [N${}_{1112(\mathrm{OH})}$][C${}_{4}$F${}_{9}$SO${}_{3}$]-rich phase and [N${}_{1112(\mathrm{OH})}$][H${}_{2}$PO${}_{4}$]-rich phase in preserving the α-helical content of IFN-α2b and BSA against thermal stress, respectively. Further, BSA is stabilized in the [N${}_{1112(\mathrm{OH})}$][H${}_{2}$PO${}_{4}$]-rich phase at concentrations of up to 415 mM [N${}_{1112(\mathrm{OH})}$][C${}_{4}$F${}_{9}$SO${}_{3}$] and 4758 mM [N${}_{1112(\mathrm{OH})}$][H${}_{2}$PO${}_{4}$].

DSC was used to probe BSA stability in the [N${}_{1112(\mathrm{OH})}$][H${}_{2}$PO${}_{4}$]-rich phase of the BP#8 system analyzed by CD for both proteins. The effect of the [N${}_{1112(\mathrm{OH})}$][H${}_{2}$PO${}_{4}$]-phase composition, 415 mM [N${}_{1112(\mathrm{OH})}$][C${}_{4}$F${}_{9}$SO${}_{3}$] and 4758 mM [N${}_{1112(\mathrm{OH})}$][H${}_{2}$PO${}_{4}$], on BSA T${}_{m}$ and protein stabilization is addressed in Table 6 and Figure S14 in the Supplementary Materials. Due to the high concentration of FIL in the FIL-rich phase, it is not technically feasible to run a DSC of the FIL-rich phase of a FIL-based ABS. A representative normalized DSC curve is depicted in Figure S14 in the Supplementary Materials for 1.2–1.6 mg/mL BSA in the [N${}_{1112(\mathrm{OH})}$][H${}_{2}$PO${}_{4}$]-rich phase (partition of 1.0 mg/mL BSA in BP#8), which allows determining a protein T${}_{m}$ of 43.61 ± 0.01 °C. A comparison with the set of representative normalized DSC curves of 1.0 mg/mL BSA in water (T${}_{m}$ of 75.59 ± 3.68 °C, viz. Figure S2 in the Supplementary Materials) was attained, showing that BSA T${}_{m}$ decreased significantly in the [N${}_{1112(\mathrm{OH})}$][H${}_{2}$PO${}_{4}$]-rich phase of the BP#8 system. T${}_{m}$ (the temperature at which both folded and unfolded states of the protein coexist at equilibrium) values of 1.0 mg/mL BSA in water and 1.2–1.6 mg/mL BSA in the [N${}_{1112(\mathrm{OH})}$][H${}_{2}$PO${}_{4}$]-rich phase (partition of 1.0 mg/mL BSA in BP#8) are listed in Table 6. The result is in opposition to the one discussed above for the CD thermal analysis, where the BSA T${}_{m}$ is increased in the [N${}_{1112(\mathrm{OH})}$][H${}_{2}$PO${}_{4}$]-rich phase, and BSA is stabilized in the [N${}_{1112(\mathrm{OH})}$][H${}_{2}$PO${}_{4}$]-rich phase at concentrations of up to 415 mM [N${}_{1112(\mathrm{OH})}$][C${}_{4}$F${}_{9}$SO${}_{3}$] and 4758 mM [N${}_{1112(\mathrm{OH})}$][H${}_{2}$PO${}_{4}$]. The concentration of ABS phase-forming components is higher than 200 mM (above the 3rd CAC of [N${}_{1112(\mathrm{OH})}$][C${}_{4}$F${}_{9}$SO${}_{3}$], viz. Table S1 in the Supplementary Materials), as analyzed in Section 2.2. These results are indicative of the formation of complexes between BSA and the ABS phase-forming components, namely with the FIL [N${}_{1112(\mathrm{OH})}$][C${}_{4}$F${}_{9}$SO${}_{3}$] aggregates, as demonstrated in previous works [37,74,75,77,98,101]. In those works, we demonstrated that the formation of complexes between FILs aggregates with selected proteins (IFN-α2b, BSA, and Lys) allows, upon centrifugation, extracting the complexed protein from the solution [37,74,75,98]. Accordingly, we selected another [N${}_{1112(\mathrm{OH})}$][H${}_{2}$PO${}_{4}$]-based system, 30 %wt [C${}_{2}$C${}_{1}$Im][C${}_{4}$F${}_{9}$SO${}_{3}$] + 6 %wt [N${}_{1112(\mathrm{OH})}$][H${}_{2}$PO${}_{4}$] (BP#5), where the BSA-enriched phase is the FIL [C${}_{2}$C${}_{1}$Im][C${}_{4}$F${}_{9}$SO${}_{3}$]-rich phase, to analyze the BSA precipitated from the [C${}_{2}$C${}_{1}$Im][C${}_{4}$F${}_{9}$SO${}_{3}$]-rich phase, resuspended in water. A representative normalized DSC curve is depicted in Figure S14 in the Supplementary Materials for 0.25 mg/mL of resuspended BSA in water (BSA precipitated from the [C${}_{2}$C${}_{1}$Im][C${}_{4}$F${}_{9}$SO${}_{3}$]-rich phase of BP#5, and resuspended in water) and 0.25 mg/mL of BSA in water, which allowed determining protein T${}_{m}$ values of 78.79 ± 0.02 °C and 79.86 ± 0.014 °C, respectively. The T${}_{m}$ values are listed in Table 6, showing that the partitioned BSA T${}_{m}$ is maintained, and that the [C${}_{2}$C${}_{1}$Im][C${}_{4}$F${}_{9}$SO${}_{3}$]-rich phase is a stabilizing medium.

#### 2.3.3. IFN Alpha-2b and BSA Simultaneous Partition

The IFN-α2b and BSA extraction efficiency (%EE), IFN-α2b purification factor ($P_{F\;1}$, and $P_{F\;2}$) attained from the individual partition studies (Section 2.3.1), depicted in Figure 5 and Table 4, suggest that the systems 30 %wt [C${}_{2}$C${}_{1}$Im][C${}_{4}$F${}_{9}$SO${}_{3}$] + 2 %wt K${}_{3}$PO${}_{4}$ (BP#2), 30 %wt [C${}_{2}$C${}_{1}$Im][C${}_{4}$F${}_{9}$SO${}_{3}$] + 20 %wt [N${}_{1112(\mathrm{OH})}$][H${}_{2}$PO${}_{4}$] (BP#7), 30 %wt [N${}_{1112(\mathrm{OH})}$][C${}_{4}$F${}_{9}$SO${}_{3}$] + 30 %wt [N${}_{1112(\mathrm{OH})}$][H${}_{2}$PO${}_{4}$] (BP#8), and 30 %wt [C${}_{4}$C${}_{1}$Im][CF${}_{3}$SO${}_{3}$] + 25 %wt sucrose (BP#3)allows a single-step simultaneous purification of IFN-α2b and BSA. Three of the biphasic systems are FIL-based ([C${}_{2}$C${}_{1}$Im][C${}_{4}$F${}_{9}$SO${}_{3}$] and [N${}_{1112(\mathrm{OH})}$][C${}_{4}$F${}_{9}$SO${}_{3}$]), and for those systems, the IFN-enriched phase is the FIL-rich phase (BP #2, BP#7, and BP#8). The rich-aggregation behavior of the [C${}_{2}$C${}_{1}$Im][C${}_{4}$F${}_{9}$SO${}_{3}$] and [N${}_{1112(\mathrm{OH})}$][C${}_{4}$F${}_{9}$SO${}_{3}$] FILs in aqueous media [37,71,77,101] and the reported aggregation between interferon and albumin [102,103], which could modify the individual partition behaviors of both proteins, led to the selection of these three FIL-based biphasic systems for assessing the single-step simultaneous purification of IFN-α2b and BSA.

**Figure 7:**
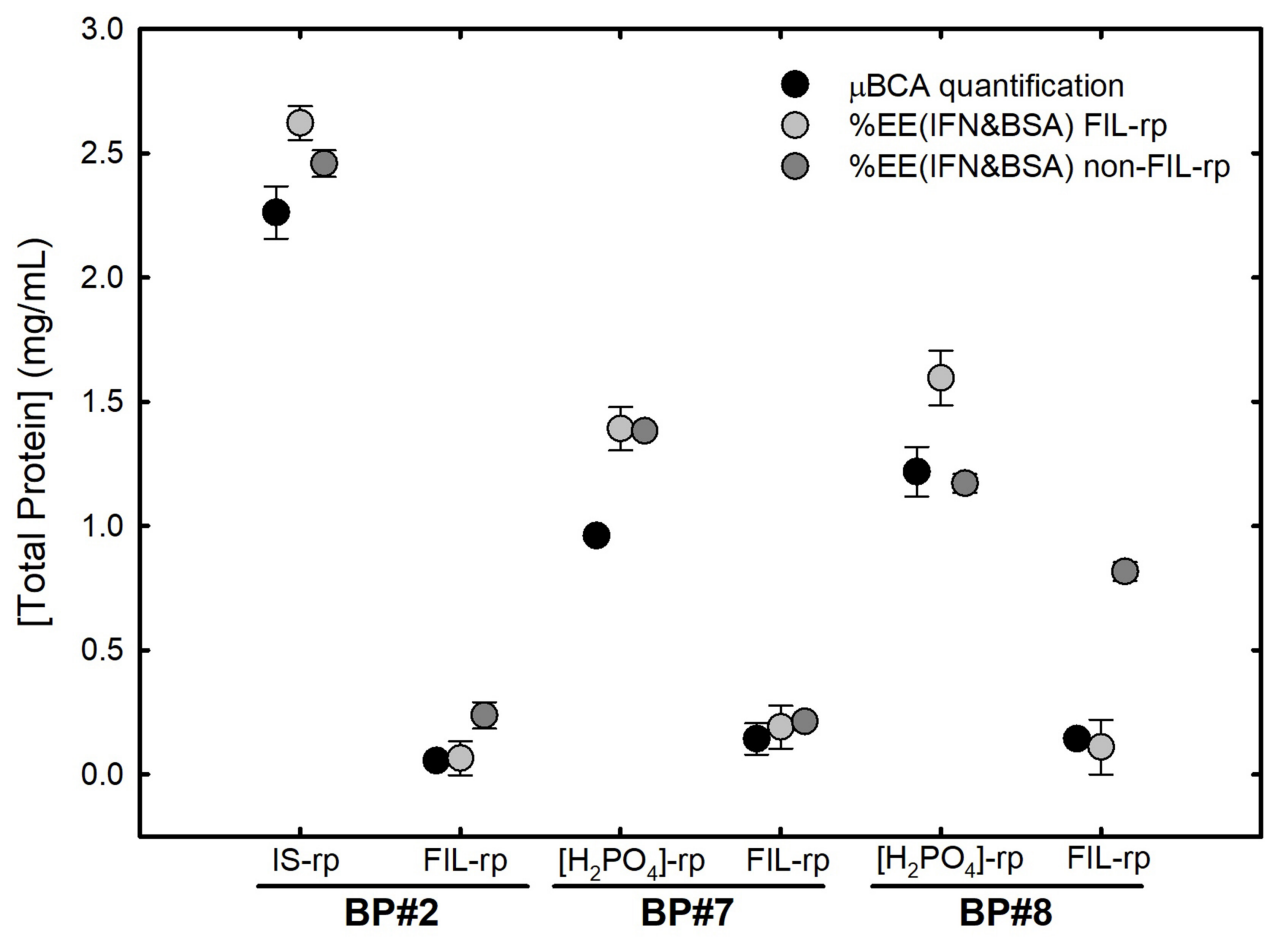
Total protein concentration (IFN-α2b + BSA), quantified through the MICRO BCA protein assay, in both phases of the biphasic points, i.e., BP#2 (30 %wt [C${}_{2}$C${}_{1}$Im][C${}_{4}$F${}_{9}$SO${}_{3}$] + 2 %wt K${}_{3}$PO${}_{4}$), BP#7 (30 %wt [C${}_{2}$C${}_{1}$Im][C${}_{4}$F${}_{9}$SO${}_{3}$] + 20 %wt [N${}_{1112(\mathrm{OH})}$][H${}_{2}$PO${}_{4}$]), and BP#8 (30 %wt [N${}_{1112(\mathrm{OH})}$][C${}_{4}$F${}_{9}$SO${}_{3}$]+ 30 %wt [N${}_{1112(\mathrm{OH})}$][H${}_{2}$PO${}_{4}$]), in the simultaneous partitions of IFN-α2b and BSA. These values are compared to the calculated total protein concentrations from individual IFN-α2b and BSA partitions using both %EE values from the FIL-rich phase and non-FIL-rich phase (Table 4).

For the individual partition studies, the precise quantification of the total protein in both phases of each biphasic system was determined through the MICRO BCA protein assay, and compared to the sum of IFN-α2b and BSA from the individual partition, as summarized in Table 7. The results are shown in Figure 7, demonstrating that the individual partition behavior of IFN-α2b and BSA is maintained in the simultaneous partition of both proteins, and that the suggested single-step simultaneous purification of IFN-α2b and BSA is verified (one-way ANOVA, with a significance level of 0.05). For the [N${}_{1112(\mathrm{OH})}$][H${}_{2}$PO${}_{4}$]-rich phase of BP#7 and the [N${}_{1112(\mathrm{OH})}$][C${}_{4}$F${}_{9}$SO${}_{3}$]-rich phase of BP#8, the total protein concentrations are statistically different; however, their phase counterparts ([C${}_{2}$C${}_{1}$Im][C${}_{4}$F${}_{9}$SO${}_{3}$] and [N${}_{1112(\mathrm{OH})}$][H${}_{2}$PO${}_{4}$] respectively) are statistically not different, confirming the single-step simultaneous purification of IFN-α2b and BSA.

**Figure 8:**
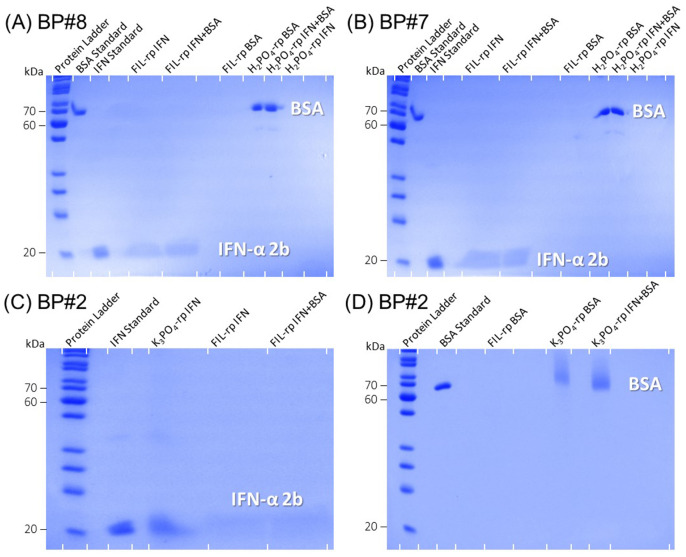
SDS-PAGE analysis of the standard protein marker (protein ladder), IFN-α2b and BSA standards, samples from the FIL-rich phase and non-FIL-rich phase ([N${}_{1112(\mathrm{OH})}$][H${}_{2}$PO${}_{4}$]-rich phase and K${}_{3}$PO${}_{4}$-rich phase) from individual partitions (lane label, IFN or BSA), and FIL-rich phase and non-FIL-rich phase samples from simultaneous partitions (lane label, IFN + BSA) stained with Coomassie blue. (**A**) BP#8, 30 %wt [N${}_{1112(\mathrm{OH})}$][C${}_{4}$F${}_{9}$SO${}_{3}$] + 30 %wt [N${}_{1112(\mathrm{OH})}$][H${}_{2}$PO${}_{4}$]. (**B**) BP#7, 30 %wt [C${}_{2}$C${}_{1}$Im][C${}_{4}$F${}_{9}$SO${}_{3}$] + 20 %wt [N${}_{1112(\mathrm{OH})}$][H${}_{2}$PO${}_{4}$]. (**C**,**D**) BP#2, 30 %wt [C${}_{2}$C${}_{1}$Im][C${}_{4}$F${}_{9}$SO${}_{3}$] + 2 %wt K${}_{3}$PO${}_{4}$. All SDS-PAGE profiles are identified at the top of each gel image. A detailed identification of all lanes is summarized in Table S4 in the Supplementary Materials.

Additionally, both phases of these three FIL-based ABSs (BP#2, BP#7, and BP#8) in the simultaneous partitions of IFN-α2b and BSA were analyzed using the SDS-PAGE method to assess the purity of IFN-α2b and BSA. Figure 8 shows the SDS-PAGE analysis (the monochromatic version is displayed in Figure S15 in the Supplementary Materials), displaying the SDS-PAGE profile of the standard protein marker (protein ladder, lane 1), IFN-α2b and BSA standards, FIL-rich phase samples, non-FIL-rich phase ([N${}_{1112(\mathrm{OH})}$][H${}_{2}$PO${}_{4}$]-rich phase and K${}_{3}$PO${}_{4}$-rich phase) samples for individual partitions (lane label, IFN, or BSA), and FIL-rich phase and non-FIL-rich phase samples for simultaneous partitions (lane label, IFN + BSA). A detailed identification of all lanes in Figure 8 is summarized in Table S4 of the Supplementary Materials. An equivalent sample of each ABS phase with approximately 0.5 μg per lane of the enriched protein was analyzed for all studied biphasic systems. Concerning the FIL-rich phases of the three FIL-based biphasic systems for the simultaneous partitions of IFN-α2b and BSA, no BSA was detected with the SDS-PAGE profiles of these phases (lane label IFN + BSA). In the non-FIL-rich phase counterparts, the K${}_{3}$PO${}_{4}$-rich phase (BP#2) and [N${}_{1112(\mathrm{OH})}$][H${}_{2}$PO${}_{4}$]-rich phase (BP#7 and BP#8), no IFN-α2b was detected with the SDS-PAGE profiles of the phases (lane label, IFN + BSA). The SDS-PAGE analysis shows high purity for IFN-α2b and BSA. The FIL-rich phase of the ABS BP#2, BP#7, and BP#8 exhibited only one band around 20 kDa (protein ladder), indicating that IFN-α2b (19 kDa) was successfully purified to the FIL-rich phase. The non-FIL-rich phase counterparts exhibited only one band between 70 kDa and 60 kDa (protein ladder), indicating that BSA (66 kDa) was simultaneously purified to the opposite phase of the optimized FIL-based ABS. The SDS-PAGE analysis confirmed the successful single-step simultaneous purification of IFN-α2b and BSA. The SDS-PAGE analysis was also carried out for individual partitions (lane label, IFN, and BSA), to provide more insight into the purification performance of the investigated FIL-based ABS. The SDS-PAGE profiles of the FIL-rich phases ([C${}_{2}$C${}_{1}$Im][C${}_{4}$F${}_{9}$SO${}_{3}$] and [N${}_{1112(\mathrm{OH})}$][C${}_{4}$F${}_{9}$SO${}_{3}$]) and [N${}_{1112(\mathrm{OH})}$][H${}_{2}$PO${}_{4}$]-rich phase of the biphasic systems, BP#8 and BP#7 (Figure 8A,B), are similar to the ones for simultaneous partitions (lane label, IFN + BSA). The same is verified for the biphasic system, BP#2 (Figure 8C,D), except for the K${}_{3}$PO${}_{4}$-rich phase for IFN-α2b individual partition (Figure 8C), where the SDS-PAGE profile exhibited a band at around 20 kDa, indicating that IFN-α2b is also present in the K${}_{3}$PO${}_{4}$-rich phase. The SDS-PAGE profile of the K${}_{3}$PO${}_{4}$-rich phase for the simultaneous partitions (Figure 8D) exhibited only a band between 70 kDa and 60 kDa, indicating that only BSA is present in the K${}_{3}$PO${}_{4}$-rich phase. These SDS-PAGE profiles are in agreement with the experimental sample procedure of the SDS-PAGE analysis discussed above and with the determined detection limit of approximately 15 ng for the implemented SDS-PAGE procedure. The lane for the K${}_{3}$PO${}_{4}$-rich phase of the simultaneous partition has approximately 0.5 μg of BSA (the BSA-enriched phase is the K${}_{3}$PO${}_{4}$-rich phase) and approximately 7 ng of IFN-α2b, and the lane for the K${}_{3}$PO${}_{4}$-rich phase of the individual partition has approximately 0.5 μg of IFN-α2b.

Further, the partition patterns of IFN-α2b and BSA between the two phases for all the biphasic systems detailed in Table 4 were evaluated by SDS-PAGE (individual partition). The SDS-PAGE profiles are depicted in Figures S16 and S17 in the Supplementary Materials for IFN-α2b and BSA, respectively, corroborating the partition behavior (%EE) and IFN-α2b purification factor ($P_{F\;1}$ and $P_{F\;2}$) for all the studied biphasic systems quantified through the MICRO BCA protein assay (Table 4) for both proteins, and quantified through the Bradford and BCA protein assays (Table S3 in the Supplementary Materials) for BSA. A detailed identification of all lanes of Figures S16 and S17 are summarized in Tables S5 and S6, respectively, in the Supplementary Materials.

### 2.4. Protein Interaction with ABS Phase-Forming Components

Microscale thermophoresis is an innovative method for analyzing the movement of fluorescent molecules out of microscopic temperature gradients in significantly reduced volumes, enabling the precise measurement of binding events independent of the size and physical characteristics of the target molecules. It is widely used to investigate the binding of biomolecules (proteins, enzymes, or DNA) to small molecules (ligands, substrates, liposomes, etc). Protein–ligand binding induces variations in the size, charge, and solvation energy of the protein, which can be effectively detected through thermophoresis. Even in cases of reduced structural modifications, MST proves highly sensitive in detecting binding events by capturing the induced changes in the solvation entropy of the molecules. The alterations induced by the binding process in the thermophoresis of the fluorescent molecules can be used to determine the equilibrium dissociation constant (K${}_{d}$) [104,105]. The strength of protein–ligand binding can be described by affinity, which is inversely proportional to the K${}_{d}$. A higher K${}_{d}$ value indicates weaker binding and lower affinity between the biomolecule and the ligand [106]. MST allows for rapid and quantitative characterization of interactions based on the thermophoretic behavior of biomolecules and sensitivity to non-covalent binding [107]. The MST assay was already used to infer the interactions between Lys [78] and IFN-α2b [77] with ionic liquids, and to determine the binding affinities of interferons [108,109] and other proteins [105,110] with distinct ligands.

Herein, a commercial FITC labeling kit (Section 3) was used to fluorescently tag both IFN-α2b and BSA. Typically, in the labeling process, one amine per protein is labeled, statistically distributing the position of the dye. Consequently, the risk of the label impairing the binding is minimal, given the typical number of lysine residues in proteins [105,110]. The five ABS phase-forming components, FILs ([C${}_{2}$C${}_{1}$Im][C${}_{4}$F${}_{9}$SO${}_{3}$] and [N${}_{1112(\mathrm{OH})}$][C${}_{4}$F${}_{9}$SO${}_{3}$]), mere fluoro-containing IL ([C${}_{4}$C${}_{1}$Im][CF${}_{3}$SO${}_{3}$]), [N${}_{1112(\mathrm{OH})}$][H${}_{2}$PO${}_{4}$] and sucrose were used as ligands to study the binding with fluorescently labeled IFN-α2b (FITC-IFN-α2b) and BSA (FITC-BSA). The MST assay was performed as detailed in Section 3. The solutions were prepared with a concentration of IFN-α2b and BSA fixed at 0.41 μM in water. For IFN-α2b a concentration of 2.7 μM was also prepared to compare it with previous results on MST-binding between FILs and IFN-α2b fluorescently labeled with a different tag [77]. In each run of MST, a maximum concentration of a ligand is selected and consecutively diluted in sixteen capillaries. From the CAC values of both FILs (Table S1 in the Supplementary Materials), five different maximum concentrations for [C${}_{2}$C${}_{1}$Im][C${}_{4}$F${}_{9}$SO${}_{3}$] and four different maximum concentrations for[N${}_{1112(\mathrm{OH})}$][C${}_{4}$F${}_{9}$SO${}_{3}$] were selected to cover the range below the formation of aggregates (<1st CAC) and to study the different CACs (four CACs for [C${}_{2}$C${}_{1}$Im][C${}_{4}$F${}_{9}$SO${}_{3}$] and three CACs for [N${}_{1112(\mathrm{OH})}$][C${}_{4}$F${}_{9}$SO${}_{3}$]) [71]. The experimental conditions of the MST assay only allow the study of the aggregation phenomenon individually for the first CAC. For the MST runs with the concentration of the first capillary above the second CAC, the determined K${}_{d}$ values for the runs that yield binding are influenced by several aggregates, making it impossible to attribute the K${}_{d}$ value to a specific CAC. However, K${}_{d}$ comprises the influence of the distinct aggregates of the FIL on binding affinity with both proteins, allowing a qualitative analysis of the impact of the different aggregates on binding affinity. For the mere fluoro-containing IL ([C${}_{4}$C${}_{1}$Im][CF${}_{3}$SO${}_{3}$]), [N${}_{1112(\mathrm{OH})}$][H${}_{2}$PO${}_{4}$] and sucrose, which are ABS phase-forming components without self-aggregation behavior, two concentrations were analyzed: 12.5 mM (with the monomer range of the FILs) and 400 mM (twice the 3rd CAC of [N${}_{1112(\mathrm{OH})}$][C${}_{4}$F${}_{9}$SO${}_{3}$], the higher concentration value for the analyzed FIL CACs). For IFN-α2b the MST runs were only carried out with [C${}_{2}$C${}_{1}$Im][C${}_{4}$F${}_{9}$SO${}_{3}$] for the two IFN-α2b concentrations discussed above, 0.41 μM and 2.7 μM, due to the availability limitations of IFN-α2b. The binding affinities between IFN-α2b (2.7 μM labeled with a different fluorescent tag) and both FILs, [C${}_{2}$C${}_{1}$Im][C${}_{4}$F${}_{9}$SO${}_{3}$] and [N${}_{1112(\mathrm{OH})}$][C${}_{4}$F${}_{9}$SO${}_{3}$], were determined in a previous work [77].

**Figure 9:**
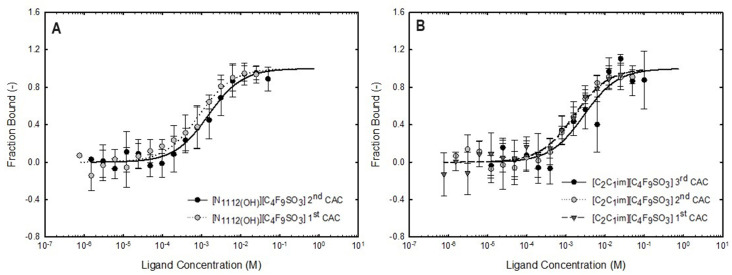
MST-binding curves of 0.41 μM FITC-BSA with (**A**) [N${}_{1112(\mathrm{OH})}$][C${}_{4}$F${}_{9}$SO${}_{3}$] and (**B**) [C${}_{2}$C${}_{1}$Im][C${}_{4}$F${}_{9}$SO${}_{3}$] at different starting concentrations. For (**A**) [N${}_{1112(\mathrm{OH})}$][C${}_{4}$F${}_{9}$SO${}_{3}$] the concentration is in the range of [185.65, 35.17] mM (K${}_{d}$ = 1.49 ± 0.30 mM), and [35.17, 16.02] mM (K${}_{d}$ = 0.86 ± 0.19 mM). For (**B**) [C${}_{2}$C${}_{1}$Im][C${}_{4}$F${}_{9}$SO${}_{3}$] the concentration was in the range of [106.09, 76.54] mM (K${}_{d}$ = 2.86 ± 1.46 mM), [76.54, 34.48] mM (K${}_{d}$ = 1.73 ± 0.42 mM), and [34.48, 14.4] mM (K${}_{d}$ = 1.61 ± 0.39 mM). The fraction bound is plotted as a function of the ligand concentration, and the curves are fitted using the kd method of the NanoTemper Analysis software. All the samples were measured in water at 25 °C. Error bars represent the standard deviations of 3 measurements. The determined K${}_{d}$ values are summarized in Table 8.

**Figure 10:**
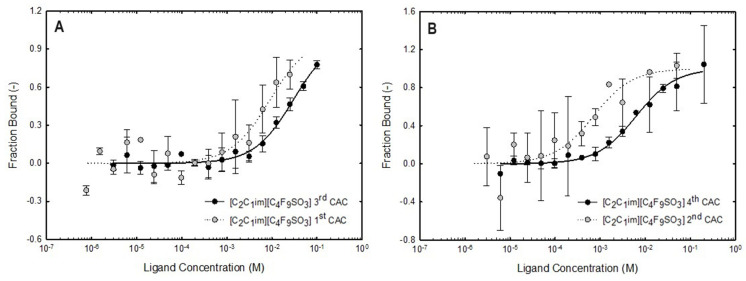
MST-binding curves of (**A**) 2.7 μM and (**B**) 0.41 μM FITC-IFN-α2b with [C${}_{2}$C${}_{1}$Im][C${}_{4}$F${}_{9}$SO${}_{3}$] at different starting concentrations (distinct FIL aggregates). FIL concentration in the range of (**A**) [106.09, 76.54] mM (K${}_{d}$ = 29.86 ± 6.26 mM) and [34.48, 14.4] mM (K${}_{d}$ = 9.10 ± 6.38 mM), and in the range of (**B**) ≥ 106.09 mM (K${}_{d}$ = 6.28 ± 1.09 mM), and [76.54, 34.48] mM (K${}_{d}$ = 0.71 ± 0.37 mM). The fraction bound is plotted as a function of the ligand concentration, and the curves are fitted using the kd method of the NanoTemper Analysis software. All the samples were measured in water at 25 °C. Error bars represent the standard deviations of 3 measurements. The determined K${}_{d}$ values are summarized in Table 8.

For the concentration range below the first CAC for both FILs, where only FIL monomers are present in the aqueous solution, no binding was detected with both proteins. The same result was attained for the ABS phase-forming components without self-aggregation behavior, [C${}_{2}$C${}_{1}$Im][C${}_{4}$F${}_{9}$SO${}_{3}$], [N${}_{1112(\mathrm{OH})}$][H${}_{2}$PO${}_{4}$] and sucrose, for the two concentrations analyzed (12.5 mM and 400 mM). These results are detailed in Table 8. Similar results were observed for Lys, a globular protein like BSA, labeled with the same fluorescent tag (FITC-Lys), at the same concentration (0.41 μM), and IFN-α2b at different concentrations (2.7 μM), labeled with a different fluorescent tag (Alexa Fluor 555). The dose–response curves of BSA-binding with [C${}_{2}$C${}_{1}$Im][C${}_{4}$F${}_{9}$SO${}_{3}$] and [N${}_{1112(\mathrm{OH})}$][C${}_{4}$F${}_{9}$SO${}_{3}$] that were fitted for K${}_{d}$ determination are depicted in Figure 9, plotting the fraction bound vs. ligand concentration. The values of K${}_{d}$ for both ligands ([C${}_{2}$C${}_{1}$Im][C${}_{4}$F${}_{9}$SO${}_{3}$] and [N${}_{1112(\mathrm{OH})}$][C${}_{4}$F${}_{9}$SO${}_{3}$]) are listed in Table 8. Analyzing the results of both FILs, the K${}_{d}$ increases with the increasing order of the CAC, indicating a weaker binding and lower affinity between BSA and FILs. The aggregates formed at lower concentrations of FIL have a higher affinity for BSA. Similar results were obtained for IFN-α2b as represented in the dose–response curves with [C${}_{2}$C${}_{1}$Im][C${}_{4}$F${}_{9}$SO${}_{3}$] in Figure 10, and the K${}_{d}$ values are listed in Table 8. The K${}_{d}$ increases with the increasing order of the CAC, indicating a weaker binding and lower affinity between IFN-α2b and [C${}_{2}$C${}_{1}$Im][C${}_{4}$F${}_{9}$SO${}_{3}$]. The aggregates formed at lower concentrations of FIL have a higher affinity for IFN-α2b. Two IFN-α2b concentrations were analyzed, 0.41 μM to compare the binding of FITC-IFN-α2b and FITC-BSA (both proteins labeled with the same fluorescent tag) with [C${}_{2}$C${}_{1}$Im][C${}_{4}$F${}_{9}$SO${}_{3}$] FIL (ABS phase-forming component presenting self-aggregation behavior), and 2.7 μM to compare the binding of IFN-α2b labeled with different fluorescent tags (FITC and Alexa Fluor 555 [77]) with [C${}_{2}$C${}_{1}$Im][C${}_{4}$F${}_{9}$SO${}_{3}$]. For FITC-IFN-α2b the trend of the K${}_{d}$ values is the same for both protein concentrations (the CAC range is interspersed between the two protein concentrations due to availability limitations of IFN-α2b). Also, the trend of the K${}_{d}$ values is the same for 2.7 μM IFN-α2b labeled with FITC and Alexa Fluor 555. However, individual K${}_{d}$ values for each CAC range depend on the protein concentration and fluorescent tag used. The trend observed for both IFN-α2b and BSA, where an increased K${}_{d}$ corresponds to assays that comprise higher concentrations of FIL (i.e., the higher order of the CAC), indicates that the aggregates formed at lower concentrations of FIL have a higher affinity for IFN-α2b and BSA, as verified in Figure 9 and Figure 10 by the deviation of the sigmoidal dose–response on the left [77,110].

The BSA binding with [C${}_{2}$C${}_{1}$Im][C${}_{4}$F${}_{9}$SO${}_{3}$] and [N${}_{1112(\mathrm{OH})}$][C${}_{4}$F${}_{9}$SO${}_{3}$] shows that the [N${}_{1112(\mathrm{OH})}$]${}^{+}$ cation has more binding affinity with BSA than the [C${}_{2}$C${}_{1}$Im]${}^{+}$ cation, demonstrated by the lower K${}_{d}$ values and the left shift of the sigmoidal in the first CAC range. The same order of the cations on IFN-α2b binding affinity with both FILs is verified [77]. Otherwise, for Lys the higher affinity is attained with the [C${}_{2}$C${}_{1}$Im]${}^{+}$ cation [78]. Further, the Lys binding with [C${}_{2}$C${}_{1}$Im][C${}_{4}$F${}_{9}$SO${}_{3}$] and [N${}_{1112(\mathrm{OH})}$][C${}_{4}$F${}_{9}$SO${}_{3}$] are distinct. With [N${}_{1112(\mathrm{OH})}$][C${}_{4}$F${}_{9}$SO${}_{3}$] K${}_{d}$ increases with the increasing order of the CAC, indicating a weaker binding and lower affinity between Lys and [N${}_{1112(\mathrm{OH})}$][C${}_{4}$F${}_{9}$SO${}_{3}$], similar to BSA and IFN-α2b. The aggregates formed at lower concentrations of [N${}_{1112(\mathrm{OH})}$][C${}_{4}$F${}_{9}$SO${}_{3}$] have a higher affinity for IFN-α2b, BSA, and Lys. With [C${}_{2}$C${}_{1}$Im][C${}_{4}$F${}_{9}$SO${}_{3}$], K${}_{d}$ decreases with the increasing order of the CAC, indicating a stronger binding and higher affinity between Lys and FILs. The aggregates formed at lower concentrations of FIL have a lower affinity for Lys. The binding between BSA and [C${}_{2}$C${}_{1}$Im][C${}_{4}$F${}_{9}$SO${}_{3}$] was previously studied by us, using isothermal titration calorimetry (ITC) [37], which is a fluorescence-labeling-free technique that rules out the potential interference of protein labeling, supporting the results attained herein with MST. The ITC results indicate that BSA interacts with [C${}_{2}$C${}_{1}$Im][C${}_{4}$F${}_{9}$SO${}_{3}$], even the FIL monomer, and it is encapsulated by FIL aggregates while its stability is improved. The MST assays carried out for IFN-α2b and BSA with all the ABS phase-forming components, FILs ([C${}_{2}$C${}_{1}$Im][C${}_{4}$F${}_{9}$SO${}_{3}$] and [N${}_{1112(\mathrm{OH})}$][C${}_{4}$F${}_{9}$SO${}_{3}$]), mere fluoro-containing IL ([C${}_{4}$C${}_{1}$Im][CF${}_{3}$SO${}_{3}$]), [N${}_{1112(\mathrm{OH})}$][H${}_{2}$PO${}_{4}$] and sucrose, used as ligands, have provided valuable information. The strong binding between IFN-α2b and BSA with the aggregates of both studied FILs and the different affinities of the distinct aggregates support the results previously discussed regarding the functionalized FIL-based ABS and the selective partitions of IFN-α2b and BSA.

## 3. Materials and Methods

### 3.1. Reagents

Human interferon-alpha 2b (IFN-α2b), ≥98 %wt SRP4595-100UG, a recombinant expressed in *E. coli*, and BSA lyophilized powder, suitable for cell culture, were from Millipore Sigma-Aldrich. Choline ((2-hydroxyethyl)trimethylammonium) dihydrogen phosphate, [N${}_{1112(\mathrm{OH})}$][H${}_{2}$PO${}_{4}$] sucrose (C${}_{12}$H${}_{22}$O${}_{11}$), ≥99.5 %wt, and potassium phosphate tribasic (K${}_{3}$PO${}_{4}$) reagent grade, ≥98 %wt salt, were purchased from Sigma-Aldrich. The Pierce${}^{\mathrm{TM}}$ FITC antibody labeling kit was purchased from Thermo Scientific. ILs 1-ethyl-3-methylimidazolium trifluoromethanesulfonate, [C${}_{2}$C${}_{1}$Im][CF${}_{3}$SO${}_{3}$] and 1-butyl-3-methylimidazolium trifluoromethanesulfonate, [C${}_{4}$C${}_{1}$Im][CF${}_{3}$SO${}_{3}$] both with > 99 %wt purity and FILs, 1-ethyl-3-methylimidazolium perfluorobutane sulfonate, [C${}_{2}$C${}_{1}$Im][C${}_{4}$F${}_{9}$SO${}_{3}$] > 99 %wt and (2-hydroxyethyl)trimethylammonium perfluorobutane sulfonate, [N${}_{1112(\mathrm{OH})}$][C${}_{4}$F${}_{9}$SO${}_{3}$] > 97 %wt were all supplied from IoLiTec GmbH. To reduce the water contents and volatile chemical impurities, all ILs were further dried at 40 ${}^{\circ }$C and 4 Pa vacuum pressure under constant stirring for at least 48 h preceding their use. After this procedure, a water content below 100 ppm was found in all ILs using a Metrohm 831 Karl Fischer coulometer. The purity of the used ILs was checked by ${}^{1}$H nuclear magnetic resonance (NMR). FIL purity was checked by ${}^{1}$H and ${}^{19}$F NMR. Double-distilled water (passed through a reverse osmosis system and further treated with Milli-Q plus 185 water purification equipment) was used in all experiments. The chemical structures of all ABS phase-forming components, FILs ([C${}_{2}$C${}_{1}$Im][C${}_{4}$F${}_{9}$SO${}_{3}$] and [N${}_{1112(\mathrm{OH})}$][C${}_{4}$F${}_{9}$SO${}_{3}$]), mere fluoro-containing ILs ([C${}_{2}$C${}_{1}$Im][CF${}_{3}$SO${}_{3}$] and [C${}_{4}$C${}_{1}$Im][CF${}_{3}$SO${}_{3}$]), globular protein stabilizers (sucrose and [N${}_{1112(\mathrm{OH})}$][H${}_{2}$PO${}_{4}$]), and high-charge-density salt (K${}_{3}$PO${}_{4}$), are listed in Table S7 of the Supplementary Materials.

### 3.2. Phase Characterization: Volume, pH, Water Content, ABS %wt Solute, Density, NMR ID

The studied biphasic points and ABS phase properties are listed in Table 1. Ternary phase diagrams for ABS composed of [C${}_{2}$C${}_{1}$Im][C${}_{4}$F${}_{9}$SO${}_{3}$] + [N${}_{1112(\mathrm{OH})}$][H${}_{2}$PO${}_{4}$] + water, [N${}_{1112(\mathrm{OH})}$][C${}_{4}$F${}_{9}$SO${}_{3}$] + [N${}_{1112(\mathrm{OH})}$][H${}_{2}$PO${}_{4}$] + water, [C${}_{2}$C${}_{1}$Im][C${}_{4}$F${}_{9}$SO${}_{3}$] + Sucrose + water, [C${}_{4}$C${}_{1}$Im][CF${}_{3}$SO${}_{3}$] + Sucrose + water, [C${}_{2}$C${}_{1}$Im][C${}_{4}$F${}_{9}$SO${}_{3}$] + K${}_{3}$PO${}_{4}$ + water, and [C${}_{2}$C${}_{1}$Im][CF${}_{3}$SO${}_{3}$] + K${}_{3}$PO${}_{4}$ + water, at 25 °C and atmospheric pressure are illustrated in Figure S1 in the Supplementary Materials. Demixing is not verified for [C${}_{2}$C${}_{1}$Im][CF${}_{3}$SO${}_{3}$] with sucrose. The determination of ternary phase diagrams and the phase properties are detailed in previous work [78]. For BSA partition assays, all ternary mixtures were prepared with 1 mg/mL of protein concentration for a total mass of 2 g. For IFN partition assays, ternary mixtures were prepared with 0.045 mg/mL of interferon concentration for a total mass of 1 g. Each mixture was vigorously stirred and centrifuged at 25 °C with 10,000 rpm for 3 min to ensure complete phase separation and partitioning between the coexisting phases at equilibrium in the stationary state. Both phases were gently separated and characterized. The pH was measured using a Mettler Toledo pH meter. Karl Fischer coulometric titration was used to determine the water content in each phase. Density was measured on an Anton Paar 75 SVM 3000, with an uncertainty of ±0.0005 g/cm${}^{3}$. To identify each phase’s main component, 1H and 19F NMR spectra were acquired for each BP system phase using a Bruker Avance 400 at 400 MHz with deuterated water solvent.

### 3.3. Protein Quantification

Two different bicinchoninic acid (BCA) methods (Pierce${}^{\mathrm{TM}}$ BCA and MICRO BCA${}^{\mathrm{TM}}$ Protein Assay KIT from Thermo Scientific) were performed for protein concentration detection on FIL-rich phases and FIL/IL-rich phases, respectively. Microplates were sealed with SecureSeal Thermal Adhesive Sealing Film and mixed on a plate shaker for 30 s prior to incubation at 37 ${}^{\circ }$C for 30 min in BCA, or 2h in MICRO BCA assays. Absorbance at 562 nm was measured on a Thermo Scientific Multiskan Go. A ready-to-use Coomassie Plus${}^{\mathrm{TM}}$ Protein Assay Reagent from Thermo Scientific was used for total protein concentration through the Bradford Assay. The microplate was mixed for 30 s and then incubated at room temperature for 10 min. Absorbance at 595 nm was measured on a Thermo Scientific Multiskan Go. The extraction efficiency was determined by the percentage ratio between the mass of protein in the IL/FIL-aqueous-rich phase (m Lys IL/FIL-rp) and the total mass of protein in the mixture. At least three individual experiments were carried out in order to determine %EE, which was calculated using the Equation (1),

(1) $$\%EE=\frac{m_{\mathrm{protein}\;\mathrm{FIL}-\mathrm{rp}}}{m_{\mathrm{protein}\;\mathrm{FIL}-\mathrm{rp}}+m_{\mathrm{protein}\;\mathrm{non}-\mathrm{FIL}-\mathrm{rp}}}.$$

To better monitor IFN-α2b and BSA purity attained by ABS partition, the purification factor of IFN-α2b was evaluated according to Equation (2) and Equation (3), respectively,

(2) $$P_{F\;(\mathrm{IL}/\mathrm{FIL}-\mathrm{rp})}\;\left(P_{F\;1}\right)=\frac{\frac{{\left[\mathrm{IFN}-\alpha 2b\right]}_{\mathrm{IL}/\mathrm{FIL}-\mathrm{rp}}}{{\left[\mathrm{total}\;\mathrm{protein}\right]}_{\mathrm{IL}/\mathrm{FIL}-\mathrm{rp}}}}{\frac{{\left[\mathrm{IFN}-\alpha 2b\right]}_{\mathrm{total}\;\mathrm{BP}}}{{\left[\mathrm{total}\;\mathrm{protein}\right]}_{\mathrm{total}\;\mathrm{BP}}}},$$

(3) $$P_{F\;(\mathrm{non}-\mathrm{IL}/\mathrm{non}-\mathrm{FIL}-\mathrm{rp})}\;\left(P_{F\;2}\right)=\frac{\frac{{\left[\mathrm{IFN}-\alpha 2b\right]}_{\mathrm{non}-\mathrm{IL}/\mathrm{non}-\mathrm{FIL}-\mathrm{rp}}}{{\left[\mathrm{total}\;\mathrm{protein}\right]}_{\mathrm{non}-\mathrm{IL}/\mathrm{non}-\mathrm{FIL}-\mathrm{rp}}}}{\frac{{\left[\mathrm{IFN}-\alpha 2b\right]}_{\mathrm{total}\;\mathrm{BP}}}{{\left[\mathrm{total}\;\mathrm{protein}\right]}_{\mathrm{total}\;\mathrm{BP}}}}.$$

### 3.4. SDS-PAGE

Sodium dodecyl sulfate-polyacrylamide gel (SDS-PAGE) was carried out in a Thermo Fisher SureCast${}^{\mathrm{TM}}$ Gel Handcast System to evaluate protein samples from all BP partition systems using a mixture of 12% acrylamide resolving gel with 4% acrylamide stacking gel for about 40 min using the Tris–Glycine SDS running buffer at constant 125 V and 3 A. IFN-α2b and BSAmixed protein samples were prepared by adding an equivalent volume of the Tris–Glycine SDS sample buffer, followed by a 5-min incubation at 85 °C. Loaded protein samples were prepared for an optimum protein mass of 0.5 μg in each gel lane whenever possible. The Thermo Scientific PageRuler Unstained Protein Ladder was directly loaded to each running gel as size standards ranging from 10 to 200 kDa. After electrophoresis, the gel was rinsed with water to remove SDS and stained for at least 3 h with a gentle shaking at 50 rpm with SimplyBlue SafeStain. A sufficient staining volume was used to fully immerse the gel in the dye. It was then de-colored in 10% NaCl and double-distilled water solution to remove unspecific staining and visualize protein bands. Each gel was finally dehydrated overnight using the DryEase™ Mini-Gel Drying System, to facilitate storage and manipulation during photographic recording.

### 3.5. DSC

Thermograms were acquired using a TA${}^{\mathrm{TM}}$ Nano DSC from TA instruments. All scans were performed in a 300 µL capillary cell through a temperature ramp from 20 to 90 °C at a heating rate of 1 °C/min and 3 atm pressure. All samples were degassed for 7 min at 20 °C, except for FIL-containing samples. After polynomial fit baselines were subtracted from the raw scans, raw thermogram curve data were fitted with the two-state scaled model using NanoAnalyze TA instruments software, version 3.12, to obtain protein thermal transition temperature (melting temperature; $T_{m}$) in each IL/FIL varying concentration sample. ΔH and ΔS were also acquired during Nano DSC scans and further used to calculate ΔG (ΔG=ΔH−TΔS). BSA partitioned in biphasic point phases was also addressed, as was BSA-precipitated from FIL-rich phases, which were centrifuged at 4 ${}^{\circ }$C for 1 h at 10,000 rpm whenever aggregation between protein and FIL was suggested. Protein interacting with the FIL, detected in the pellet, was resuspended in water, and recovered BSA was analyzed.

### 3.6. CD

Circular dichroism (CD) spectra were collected in a 10 mm quartz cuvette and a Chirascan spectropolarimeter (Applied Photophysics, UK) at room temperature. All CD spectra were recorded in the far-UV range of 190 to 260 nm at scan rates of 50 nm/min, with 4 accumulations, and a response time of 3 s; CD spectra were collected. For each CD experiment, two distinct duplicates were acquired and combined. BSA was fixed at 1 mg/mL, solvent concentrations ranging from 0.1 to 200 mM were used, and the spectra were translated to MRE after being expressed in terms of molar ellipticity. Spectral deconvolution was carried out with DichroWeb using the CONTIN-LL deconvolution algorithm with the SMP180 reference set [82].

### 3.7. MST

Microscale thermophoresis (MST) measurements of the dye-labeled protein were used to conduct binding investigations. The Thermo Scientific Pierce${}^{\mathrm{TM}}$ FITC Labeling Kit was used to label both the IFN-α2b and BSA according to the manufacturer’s recommended protocol and followed by UV/VIS spectrophotometry at 494 and 280 nm to attain the degree of labeling. MST tests were conducted in a monolith NT.115 (blue/red) in accordance with the NanoTemper Technologies methodology. Medium MST power and 20% LED power (nano-blue) were set as the instrument’s default settings. For every MST measurement, a PBS buffer was used. The concentration of FITC-BSA was fixed at 0.4 μM and FITC-IFN-α2b was tested under two different concentrations (of 0.4 and 2.7 μM). The ligand was used to create a set of 16 1:1 dilutions, with the starting ligand concentrations ranging from 10 to 400 mM. The typical parameters for MST traces were used for recording: 5 s MST power on, 30 s MST power on, and 5 s MST power off. The data from three independently conducted pipetted experiments were examined using NanoTemper Technologies’ MO.Affinity Analysis software, version 2.3.

## 4. Conclusions

In the present work, IFN-α2b and BSA (serum albumin protein) were successfully purified simultaneously, in a single step, using functionalized IL-based ABS comprising FILs ([C${}_{2}$C${}_{1}$Im][C${}_{4}$F${}_{9}$SO${}_{3}$] and [N${}_{1112(\mathrm{OH})}$][C${}_{4}$F${}_{9}$SO${}_{3}$]) vs. mere fluoro-containing IL ([C${}_{4}$C${}_{1}$Im][CF${}_{3}$SO${}_{3}$]), in combination with sucrose or [N${}_{1112(\mathrm{OH})}$][H${}_{2}$PO${}_{4}$] (a globular protein stabilizer), or even a high-charge-density salt, K${}_{3}$PO${}_{4}$. The different ABS phase parameters, such as pH, water content (%wt), composition (%wt), and volume ratio, were investigated. The phase pH was found to have a significant effect on IFN-α2b and BSA partitions. IFN-α2b and BSA were purified through selective partition into opposite phases in single-step IL-based ABS extraction. It was demonstrated that the functionalization of the ABS allows for selecting the enriched phase. The four optimized biphasic systems include three FIL-based ABS, 30 %wt [C${}_{2}$C${}_{1}$Im][C${}_{4}$F${}_{9}$SO${}_{3}$] + 20 %wt [N${}_{1112(\mathrm{OH})}$][H${}_{2}$PO${}_{4}$], 30 %wt [N${}_{1112(\mathrm{OH})}$][C${}_{4}$F${}_{9}$SO${}_{3}$] + 30 %wt [N${}_{1112(\mathrm{OH})}$][H${}_{2}$PO${}_{4}$], and 30 %wt [C${}_{2}$C${}_{1}$Im][C${}_{4}$F${}_{9}$SO${}_{3}$] + 2 %wt K${}_{3}$PO${}_{4}$, where the IFN-α2b-enriched phase is the FIL-rich phase (bottom phase) and the BSA-enriched phase encompasses the [N${}_{1112(\mathrm{OH})}$][H${}_{2}$PO${}_{4}$]-rich phase and K${}_{3}$PO${}_{4}$-rich phase (top phase). For the system comprising the mere fluoro-containing IL and sucrose, 30 %wt [C${}_{4}$C${}_{1}$Im][CF${}_{3}$SO${}_{3}$] + 25 %wt sucrose, the IFN-α2b-enriched phase is the sucrose-rich phase (top phase) and the BSA-enriched phase is the [C${}_{4}$C${}_{1}$Im][CF${}_{3}$SO${}_{3}$]-rich phase (bottom phase). The experimental results show that simultaneous single-step purification was achieved with a high yield (extraction efficiency up to 100%) for both proteins and a purification factor of IFN-α2b which is high in the enriched IFN-α2b phase (up to 23.22) and low in the BSA-enriched phase (down to 0.00). SDS-PAGE analysis confirmed the purity of both recovered proteins. The stability and structure of IFN-α2b and BSA were preserved, or even improved (FIL-rich phase) during the purification step, as evaluated by circular dichroism spectroscopy and differential scanning calorimetry. Binding studies of IFN-α2b and BSA with the ABS phase-forming components were assessed by microscale thermophoresis, indicating a strong interaction between FIL aggregates and both proteins. Interactions were not verified with the other ABS phase-forming components, including mere fluoro-containing IL and known globular protein stabilizers (sucrose and [N${}_{1112(\mathrm{OH})}$][H${}_{2}$PO${}_{4}$]). In view of their biocompatibility, customizable properties, and selectivity, FIL-based ABSs are suggested as an improved purification step for relevant biopharmaceutical proteins. These compelling results indicate that the developed functionalized ionic liquid-based ABS offer a potential alternative to more traditional PEG/salt ABSs, alcohol/salt ABSs, or even conventional chromatography steps (ion exchange, size exclusion, immobilized metal-ion affinity, or reverse-phase HPLC) in downstream processes that could facilitate the development of biologics.

## Figures and Tables

**Table 1:** Ternary mixture properties of the selected ABS (biphasic points, BP), including the volume ratio, pH, and composition of both the ionic liquid-rich phases (the FIL-rich phase and mere fluoro-containing IL-rich phase) and non-ionic liquid-rich phases (the K${}_{3}$PO${}_{4}$-rich phase, sucrose-rich phase, and [N${}_{1112(\mathrm{OH})}$][H${}_{2}$PO${}_{4}$]-rich phase), at 25 °C.

			Ionic Liquid-Rich Phase (Bottom Phase)					Non-Ionic Liquid-Rich Phase (Top Phase)			
**BP#**	**ABS Composition (%wt)**	**Volume Ratio ^†^**	**pH**	**%wt H20**	**%wt IL/FIL**	**%wt Salt**		**pH**	**%wt H20**	**%wt IL/FIL**	**%wt Salt**
BP#1	30% [C${}_{2}$C${}_{1}$Im][CF${}_{3}$SO${}_{3}$] +10% K${}_{3}$PO${}_{4}$	0.60	13.44	40.8785	56.5614	2.0652		13.18	73.4861	8.7068	16.8167
BP#2	30% [C${}_{2}$C${}_{1}$Im][C${}_{4}$F${}_{9}$SO${}_{3}$] +2% K${}_{3}$PO${}_{4}$	0.94	12.78	43.6779	54.0350	0.3349		12.70	88.5482	4.2344	6.7525
BP#3	30% [C${}_{4}$C${}_{1}$Im][CF${}_{3}$SO${}_{3}$] +25% Sucrose	0.39	5.00	31.1747	57.6533	10.3701		5.25	47.1635	17.7208	32.0208
BP#4	30% [C${}_{2}$C${}_{1}$Im][C${}_{4}$F${}_{9}$SO${}_{3}$] +25% Sucrose	0.33	6.75	27.6055	66.8372	6.2338		6.75	53.5971	15.5558	31.8248
BP#5	30% [C${}_{2}$C${}_{1}$Im][C${}_{4}$F${}_{9}$SO${}_{3}$] +6% [N${}_{1112(\mathrm{OH})}$][H${}_{2}$PO${}_{4}$]	0.89	3.37	41.3607	55.5876	1.5481		3.41	84.2108	4.0618	12.1251
BP#6	30% [C${}_{2}$C${}_{1}$Im][C${}_{4}$F${}_{9}$SO${}_{3}$] +10% [N${}_{1112(\mathrm{OH})}$][H${}_{2}$PO${}_{4}$]	0.59	3.65	30.1971	69.0974	1.0569		3.56	83.6562	2.3820	15.7034
BP#7	30% [C${}_{2}$C${}_{1}$Im][C${}_{4}$F${}_{9}$SO${}_{3}$] +20% [N${}_{1112(\mathrm{OH})}$][H${}_{2}$PO${}_{4}$]	0.42	3.99	17.7743	83.7801	0.6989		4.03	67.3516	5.1015	28.3455
BP#8	30% [N${}_{1112(\mathrm{OH})}$][C${}_{4}$F${}_{9}$SO${}_{3}$] +30% [N${}_{1112(\mathrm{OH})}$][H${}_{2}$PO${}_{4}$]	0.60	4.15	25.3343	63.9061	10.1689		4.16	45.8046	7.6946	43.9659

^†^$\frac{\mathrm{volume}\mathrm{IL}-\mathrm{rp}}{\mathrm{volume}\mathrm{non}-\mathrm{IL}-\mathrm{rp}}$.

**Table 2:** DSC melting temperature ($T_{m}$), enthalpy change (ΔH), entropy change (ΔS), and Gibbs energy change (ΔG) for 1.0 mg/mL BSA in water and aqueous solutions of an increasing concentration of ABS phase-forming components.

Concentration (mM)		T${}_{m}$ (${}^{\circ }$C)	ΔH${}_{\mathrm{cal}}$ (KJ/mol)^†^	ΔH${}_{\mathrm{vH}}$ (KJ/mol)^†^	ΔH${}_{\mathrm{cal}}$/ΔH${}_{\mathrm{vH}}$	ΔS (KJ/(mol.K))	ΔG (KJ/mol)
Water		75.59	476.4	470.3	0.997	1.361	70.60
[C${}_{2}$C${}_{1}$Im][C${}_{4}$F${}_{9}$SO${}_{3}$]	0.1	75.55	503.0	489.4	1.028	1.445	72.30
	1	75.62	592.2	563.8	1.050	1.700	85.40
	5	80.59	797.9	825.0	0.967	2.255	125.6
	10	80.94	768.1	724.1	1.061	2.168	121.7
	25	82.57	837.9	861.6	0.972	2.354	136.1
	55	84.28	1001.0	905.7	1.105	2.798	166.9
	120	86.15	830.9	790.9	1.051	2.310	142.2
[C${}_{4}$C${}_{1}$Im][CF${}_{3}$SO${}_{3}$]	0.1	74.54	513.1	542.1	0.946	1.476	72.90
	1	75.31	422.1	427.8	0.987	1.210	61.40
	5	81.43	717.0	670.6	1.069	2.021	114.4
	10	79.02	517.5	505.2	1.024	1.468	79.80
	25	72.02	336.6	339.3	0.992	0.973	46.60
	55	72.44	375.8	391.4	0.960	1.086	51.90
	120	76.32	477.1	492.3	0.969	1.363	70.70
[N${}_{1112(\mathrm{OH})}$][C${}_{4}$F${}_{9}$SO${}_{3}$]	0.1	79.77	603.1	567.0	1.064	1.708	93.90
	1	80.57	578.1	627.7	0.921	1.629	92.40
	5	79.79	708.7	801.4	0.884	2.007	110.5
	10	82.78	828.1	716.6	1.156	2.325	135.0
	25	84.18	761.3	747.2	1.019	2.128	126.8
	55	86.11	772.9	831.5	0.929	2.149	132.1
	200	87.23	702.4	685.6	1.024	1.956	119.3
[N${}_{1112(\mathrm{OH})}$][H${}_{2}$PO${}_{4}$]	0.1	75.36	442.9	466.8	0.949	1.272	63.60
	1	81.59	470.4	766.8	0.613	1.325	75.40
	5	80.80	678.6	627.7	1.081	1.915	107.6
	10	73.90	513.7	514.8	0.998	1.479	72.90
Sucrose	0.1	79.97	523.2	540.7	0.968	1.480	82.00
	1	81.63	700.6	714.5	0.980	1.974	112.0
	5	80.68	550.0	540.3	1.018	1.552	87.30
	10	80.77	665.1	635.6	1.046	1.878	105.3

^†^ calorimetric enthalpy, ΔHcal; van’t Hoff enthalpy, ΔHvH.

**Table 3:** Estimation of 0.125 mg/mL IFN-α2b and 1.0 mg/mL BSA secondary structure contents from CD spectra deconvolution using the CONTIN-LL algorithm [82], at 25 ${}^{\circ }$C.

	Conc. (mM)	Regular Alpha Helix	Distorted Alpha Helix	Regular Beta Sheet	Distorted Beta Sheet	Turns	Unordered	Total
				**IFN-α2b**				
Water	0	0.289	0.145	0.049	0.071	0.209	0.238	1.001
[C${}_{2}$C${}_{1}$Im][C${}_{4}$F${}_{9}$SO${}_{3}$]	200	0.292	0.153	0.05	0.069	0.204	0.232	1.000
[N${}_{1112(\mathrm{OH})}$][C${}_{4}$F${}_{9}$SO${}_{3}$]	200	0.303	0.148	0.071	0.064	0.188	0.226	1.000
				**BSA**				
Water	0	0.400	0.206	0.000	0.025	0.136	0.233	1.000
[C${}_{2}$C${}_{1}$Im][C${}_{4}$F${}_{9}$SO${}_{3}$]	0.1	0.407	0.206	0.000	0.029	0.142	0.216	1.000
	1	0.401	0.210	0.000	0.025	0.137	0.226	0.999
	5	0.406	0.207	0.007	0.027	0.138	0.215	1.000
	25	0.384	0.197	0.023	0.035	0.149	0.212	1.000
	55	0.402	0.210	0.000	0.026	0.140	0.223	1.001
	90	0.382	0.207	0.004	0.026	0.142	0.239	1.000
	120	0.405	0.206	0.014	0.032	0.147	0.196	1.000
	200	0.316	0.183	0.014	0.025	0.108	0.354	1.000
[C${}_{4}$C${}_{1}$Im][CF${}_{3}$SO${}_{3}$]	0.1	0.403	0.207	0.008	0.028	0.141	0.212	0.999
	1	0.419	0.215	0.000	0.023	0.135	0.208	1.000
	5	0.403	0.209	0.000	0.025	0.136	0.227	1.000
	25	0.297	0.180	0.020	0.031	0.128	0.344	1.000
	55	0.390	0.217	0.000	0.021	0.131	0.240	0.999
	90	0.382	0.202	0.007	0.026	0.136	0.247	1.000
	120	0.378	0.212	0.000	0.019	0.126	0.266	1.001
	200	0.411	0.223	0.000	0.024	0.136	0.206	1.000
[N${}_{1112(\mathrm{OH})}$][C${}_{4}$F${}_{9}$SO${}_{3}$]	0.1	0.418	0.214	0.000	0.025	0.138	0.205	1.000
	1	0.428	0.225	0.000	0.024	0.138	0.185	1.000
	5	0.423	0.222	0.000	0.025	0.139	0.192	1.001
	25	0.406	0.215	0.000	0.025	0.140	0.214	1.000
	55	0.392	0.208	0.000	0.027	0.144	0.228	0.999
	90	0.376	0.199	0.022	0.036	0.152	0.215	1.000
	120	0.400	0.205	0.012	0.032	0.147	0.203	0.999
	200	0.406	0.207	0.017	0.032	0.147	0.191	1.000
[N${}_{1112(\mathrm{OH})}$][H${}_{2}$PO${}_{4}$]	0.1	0.397	0.204	0.000	0.031	0.147	0.221	1.000
	1	0.393	0.210	0.000	0.023	0.132	0.242	1.000
	5	0.377	0.203	0.004	0.023	0.128	0.265	1.000
	25	0.36	0.195	0.015	0.029	0.136	0.266	1.001
	55	0.242	0.150	0.080	0.055	0.153	0.320	1.000
	90	0.294	0.171	0.057	0.044	0.145	0.289	1.000
	120	0.396	0.207	0.018	0.029	0.141	0.208	0.999
	200	0.386	0.202	0.021	0.032	0.146	0.212	0.999
Sucrose	0.1	0.391	0.205	0.000	0.026	0.136	0.242	1.000
	1	0.428	0.216	0.000	0.023	0.135	0.198	1.000
	5	0.427	0.225	0.000	0.022	0.136	0.19	1.000
	25	0.333	0.196	0.004	0.024	0.131	0.312	1.000
	55	0.412	0.211	0.000	0.026	0.138	0.214	1.001
	90	0.4	0.211	0.000	0.025	0.137	0.227	1.000
	120	0.415	0.212	0.000	0.025	0.135	0.213	1.000
	200	0.436	0.216	0.000	0.024	0.136	0.189	1.001

**Table 4:** IFN-α2b and BSA extraction efficiency (%EE; Equation (1)) and IFN-α2b purification factor (IL/FIL-rp, $P_{F\;1}$; non-IL/non-FIL-rp, $P_{F\;2}$) determined for the studied biphasic systems. Protein concentrations in both ABS phases were quantified by the MICRO BCA protein assay. The %EE values are the results of at least 3 partition experiments.

BP#	ABS Composition (%wt)	%EE IFN	$P_{F\;1}{}^{†}$	%EE IFN	$P_{F\;1}{}^{†}$	%EE BSA	$P_{F\;2}{}^{‡}$	%EE BSA	$P_{F\;2}{}^{‡}$
		**(from IL/FIL-rp)**		**(from H2PO4/IS/HC-rp)**		**(from IL/FIL-rp)**		**(from H2PO4/IS/HC-rp)**	
BP#1	30% [C${}_{2}$C${}_{1}$Im][CF${}_{3}$SO${}_{3}$] +10% K${}_{3}$PO${}_{4}$	91.77 ± 13.21	0.96	100.00 ${}^{§}$	1.00	95.86 ± 5.54	1.91	100.00 ${}^{§}$	0.00
BP#2	30% [C${}_{2}$C${}_{1}$Im][C${}_{4}$F${}_{9}$SO${}_{3}$] +2% K${}_{3}$PO${}_{4}$	70.07 ± 6.79	23.22	100.00 ${}^{§}$	10.69	0.00 ${}^{§}$	0.31	5.28 ± 5.33	0.00
BP#3	30% [C${}_{4}$C${}_{1}$Im][CF${}_{3}$SO${}_{3}$] +25% Sucrose	0.00 ${}^{§}$	0.00	10.00 ± 0.60	0.10	84.57 ± 1.27	5.24	100.00 ${}^{§}$	23.22
BP#4	30% [C${}_{2}$C${}_{1}$Im][C${}_{4}$F${}_{9}$SO${}_{3}$] +25% Sucrose	0.00 ${}^{§}$	0.00	10.43 ± 0.61	3.02	0.00 ${}^{§}$	1.00	3.14 ± 4.45	0.93
BP#5	30% [C${}_{2}$C${}_{1}$Im][C${}_{4}$F${}_{9}$SO${}_{3}$] +6% [N${}_{1112(\mathrm{OH})}$][H${}_{2}$PO${}_{4}$]	99.56 ± 14.09	1.24	100.00 ${}^{§}$	1.00	79.45 ± 10.43	0.02	100.00 ${}^{§}$	0.00
BP#7	30% [C${}_{2}$C${}_{1}$Im][C${}_{4}$F${}_{9}$SO${}_{3}$] +20% [N${}_{1112(\mathrm{OH})}$][H${}_{2}$PO${}_{4}$]	80.45 ± 8.70	15.26	100.00 ${}^{§}$	16.98	1.89 ± 0.24	0.21	1.65 ± 0.08	0.00
BP#8	30% [N${}_{1112(\mathrm{OH})}$][C${}_{4}$F${}_{9}$SO${}_{3}$] +30% [N${}_{1112(\mathrm{OH})}$][H${}_{2}$PO${}_{4}$]	82.58 ± 11.01	23.22	100.00 ${}^{§}$	3.43	0.00 ${}^{§}$	0.18	25.94 ± 3.79	0.00

^†^ IL/FIL-rp purification factor of IFN-α2b (Equation (2)); ^‡^ non-IL/non-FIL-rp purification factor of IFN-α2b (Equation (3)); ^§^ no standard deviation due to protein quantification under the detection limit (0.002 mg/mL).

**Table 5:** T${}_{m}$ (°C) of IFN-α2b in the [N${}_{1112(\mathrm{OH})}$][C${}_{4}$F${}_{9}$SO${}_{3}$]-rich phase and BSA in the [N${}_{1112(\mathrm{OH})}$][H${}_{2}$PO${}_{4}$]-rich phase from individual partition in BP#8 ABS, 30 %wt [N${}_{1112(\mathrm{OH})}$][C${}_{4}$F${}_{9}$SO${}_{3}$] + 30 %wt [N${}_{1112(\mathrm{OH})}$][H${}_{2}$PO${}_{4}$] (partition for the analyzed phase; Table 4), determined using CD spectra thermal analysis (Figure 6 and Figure S13 in the Supplementary Materials); 0.125 mg/mL of IFN-α2b and 1.0 mg/mL of BSA in water; 0.099 mg/mL (from the [C${}_{2}$C${}_{1}$Im][C${}_{4}$F${}_{9}$SO${}_{3}$]-rich phase) or 0.12 mg/mL (from the [N${}_{1112(\mathrm{OH})}$][H${}_{2}$PO${}_{4}$]-rich phase) of IFN-α2b in the [N${}_{1112(\mathrm{OH})}$][C${}_{4}$F${}_{9}$SO${}_{3}$]-rich phase; 1.6 mg/mL (from the [C${}_{2}$C${}_{1}$Im][C${}_{4}$F${}_{9}$SO${}_{3}$]-rich phase) or 1.2 mg/mL (from the [N${}_{1112(\mathrm{OH})}$][H${}_{2}$PO${}_{4}$]-rich phase) of BSA in the [N${}_{1112(\mathrm{OH})}$][H${}_{2}$PO${}_{4}$]-rich phase. Protein concentrations in both ABS phases were quantified by the MICRO BCA protein assay.

		Tm (°C)		
		**Ellipticity** **222 nm**	**Ellipticity** **208 nm**	**Ellipticity** **192 nm**
IFN	Water	67.548	66.156	63.311
	[N${}_{1112(\mathrm{OH})}$][C${}_{4}$F${}_{9}$SO${}_{3}$]-rp	62.080	71.217	42.274
BSA	Water	76.277	74.616	74.028
	[N${}_{1112(\mathrm{OH})}$][H${}_{2}$PO${}_{4}$]-rp	80.396	76.122	75.765

**Table 6:** DSC melting temperature $T_{m}$ (°C) for 0.25 mg/mL and 1.0 mg/mL BSA in water, 0.25 mg/mL BSA resuspended in water (recover from the [C${}_{2}$C${}_{1}$Im][C${}_{4}$F${}_{9}$SO${}_{3}$]-rich phase of BP#5 in the partition of 1.0 mg/mL BSA, 30 %wt [C${}_{2}$C${}_{1}$Im][C${}_{4}$F${}_{9}$SO${}_{3}$] + 6% wt[N${}_{1112(\mathrm{OH})}$][H${}_{2}$PO${}_{4}$], and resuspended in water), and 1.2–1.6 mg/mL BSA in the [N${}_{1112(\mathrm{OH})}$][H${}_{2}$PO${}_{4}$]-rich phase (partition of 1.0 mg/mL BSA in BP#8, 30 %wt [N${}_{1112(\mathrm{OH})}$][C${}_{4}$F${}_{9}$SO${}_{3}$] +30 %wt [N${}_{1112(\mathrm{OH})}$][H${}_{2}$PO${}_{4}$]). All DSC curves are depicted in Figure S14 in the Supplementary Materials.

BSA 2.5 mg/mL	
Water	77.35 ± 0.025
[N${}_{1112(\mathrm{OH})}$][H${}_{2}$PO${}_{4}$]-rp (BP#8)	43.61 ± 0.011
**BSA 0.25 mg/mL**	
Water	79.86 ± 0.014
resuspended in Water	78.73 ± 0.019
(BSA recover from [C${}_{2}$C${}_{1}$Im][C${}_{4}$F${}_{9}$SO${}_{3}$]-rich phase BP#5)	

**Table 7:** Total protein concentration and mass (IFN-α2b + BSA), quantified through the MICRO BCA protein assay, in both phases of the biphasic points, BP#2 (30 %wt [C${}_{2}$C${}_{1}$Im][C${}_{4}$F${}_{9}$SO${}_{3}$] + 2 %wt K${}_{3}$PO${}_{4}$), BP#7 (30 %wt [C${}_{2}$C${}_{1}$Im][C${}_{4}$F${}_{9}$SO${}_{3}$] + 20 %wt [N${}_{1112(\mathrm{OH})}$][H${}_{2}$PO${}_{4}$]), and BP#8 (30 %wt [N${}_{1112(\mathrm{OH})}$][C${}_{4}$F${}_{9}$SO${}_{3}$]+ 30 %wt [N${}_{1112(\mathrm{OH})}$][H${}_{2}$PO${}_{4}$]), in the simultaneous partitions of IFN-α2b and BSA. These values are compared to the calculated total protein mass and concentration from individual IFN-α2b and BSA partitions using both %EE values from the FIL-rich phase and non-FIL-rich phase (Table 4).

		μBCA			FIL-rp			non-FIL-rp	
		**[Total Protein]**	**m Total Protein**		**mIFN + mBSa**	**[IFN + BSA]**		**mIFN + mBSA**	**[IFN + BSA]**
		**(mg/mL)**	**(mg)**		**(mg)**	**(mg/mL)**		**(mg)**	**(mg/mL)**
BP#2	K${}_{3}$PO${}_{4}$-rp	2.26 ± 0.11	1.02		1.18	2.62 ± 0.07		1.11	2.46 ± 0.05
	[C${}_{2}$C${}_{1}$Im][C${}_{4}$F${}_{9}$SO${}_{3}$]-rp	0.05 ± 0.00	0.02		0.03	0.06 ± 0.07		0.10	0.24 ± 0.05
BP#7	[N${}_{1112(\mathrm{OH})}$][H${}_{2}$PO${}_{4}$]-rp	0.96 ± 0.00	0.58		0.84	1.39 ± 0.09		0.83	1.38 ± 0.00
	[C${}_{2}$C${}_{1}$Im][C${}_{4}$F${}_{9}$SO${}_{3}$]-rp	0.14 ± 0.06	0.04		0.05	0.19 ± 0.09		0.05	0.21 ± 0.00
BP#8	[N${}_{1112(\mathrm{OH})}$][H${}_{2}$PO${}_{4}$]-rp	1.22 ± 0.10	0.61		0.80	1.60 ± 0.11		0.59	1.17 ± 0.04
	[N${}_{1112(\mathrm{OH})}$][C${}_{4}$F${}_{9}$SO${}_{3}$]-rp	0.14 ± 0.00	0.04		0.03	0.11 ± 0.11		0.24	0.82 ± 0.04

**Table 8:** Dissociation constant K${}_{d}$ for FITC-IFN-α2b and FITC-BSA with different ligands, FILs ([C${}_{2}$C${}_{1}$Im][C${}_{4}$F${}_{9}$SO${}_{3}$] and [N${}_{1112(\mathrm{OH})}$][C${}_{4}$F${}_{9}$SO${}_{3}$]), mere fluoro-containing IL ([C${}_{4}$C${}_{1}$Im][CF${}_{3}$SO${}_{3}$]), and the known globular protein stabilizers ([N${}_{1112(\mathrm{OH})}$][H${}_{2}$PO${}_{4}$] and sucrose), determined by the analysis of the fitting of the MST dose–curve responses (Figure 10 and Figure 9). The concentration range of the first capillary (maximum concentration) as well as the corresponding aggregates (or monomers) of the ligand, are depicted.

Ligand	Max. Conc. (mM) (First Capillary)	K${}_{d}$ (mM)
**2.7 μM IFN-α2b**		
[C${}_{2}$C${}_{1}$Im][C${}_{4}$F${}_{9}$SO${}_{3}$]	]106.09, 76.54] (3rd CAC)	29.86 ± 6.27
	]34.48, 14.40] (1st CAC)	9.10 ± 6.38
**0.41 μM IFN-α2b**		
[C${}_{2}$C${}_{1}$Im][C${}_{4}$F${}_{9}$SO${}_{3}$]	≥106.09 (4th CAC)	6.28 ± 1.09
	]76.54, 34.48] (2nd CAC)	0.71 ± 0.37
	]14.40, 0[ (monomer)	no binding detected
**0.41 μM BSA**		
[C${}_{2}$C${}_{1}$Im][C${}_{4}$F${}_{9}$SO${}_{3}$]	≥106.09 (4th CAC)	no binding detected
	]106.09, 76.54] (3rd CAC)	2.86 ± 1.46
	]76.54, 34.48] (2nd CAC)	1.73 ± 0.42
	]34.48, 14.4] (1st CAC)	1.61 ± 0.39
	]14.40, 0[ (monomer)	no binding detected
**0.41 μM BSA**		
[N${}_{1112(\mathrm{OH})}$][C${}_{4}$F${}_{9}$SO${}_{3}$]	≥185.65 (3rd CAC)	no binding detected
	]185.65, 35.17] (2nd CAC)	1.49 ± 0.30
	]35.17, 16.02] (1st CAC)	0.86 ± 0.19
	]16.02, 0[ (monomer)	no binding detected
**0.41 μM BSA**		
[C${}_{4}$C${}_{1}$Im][CF${}_{3}$SO${}_{3}$]	400 (monomer)	no binding detected
	12.5 (monomer)	
**0.41 μM BSA**		
[N${}_{1112(\mathrm{OH})}$][H${}_{2}$PO${}_{4}$]	400 (monomer)	no binding detected
	12.5 (monomer)	
**0.41 μM BSA**		
Sucrose	400 (monomer)	no binding detected
	12.5 (monomer)	

## Data Availability

Data will be made available upon request.

## Supplementary Materials

The following supporting information can be downloaded at https://www.mdpi.com/article/10.3390/ijms25052751/s1.
