# Supplementary material for: Simultaneous Purification of Human Interferon Alpha-2b and Serum Albumin Using Bioprivileged Fluorinated Ionic Liquid-Based Aqueous Biphasic Systems
Source: Int J Mol Sci. 2024 Feb 27;25(5):2751. doi: 10.3390/ijms25052751 (PMC10931833; doi:10.3390/ijms25052751)
Supplement: Supplementary file 1 [file ijms-25-02751-s001.zip › ijms-2880926-supplementary.pdf]

Supplementary Materials: Simultaneous Purification of Human Interferon Alpha-2b and Serum Albumin using Bioprivileged Fluorinated Ionic Liquid-based Aqueous Biphasic Systems

Sara F. Carvalho [1], Ana B. Pereiro [1] and João M. M. Araújo [1,*]

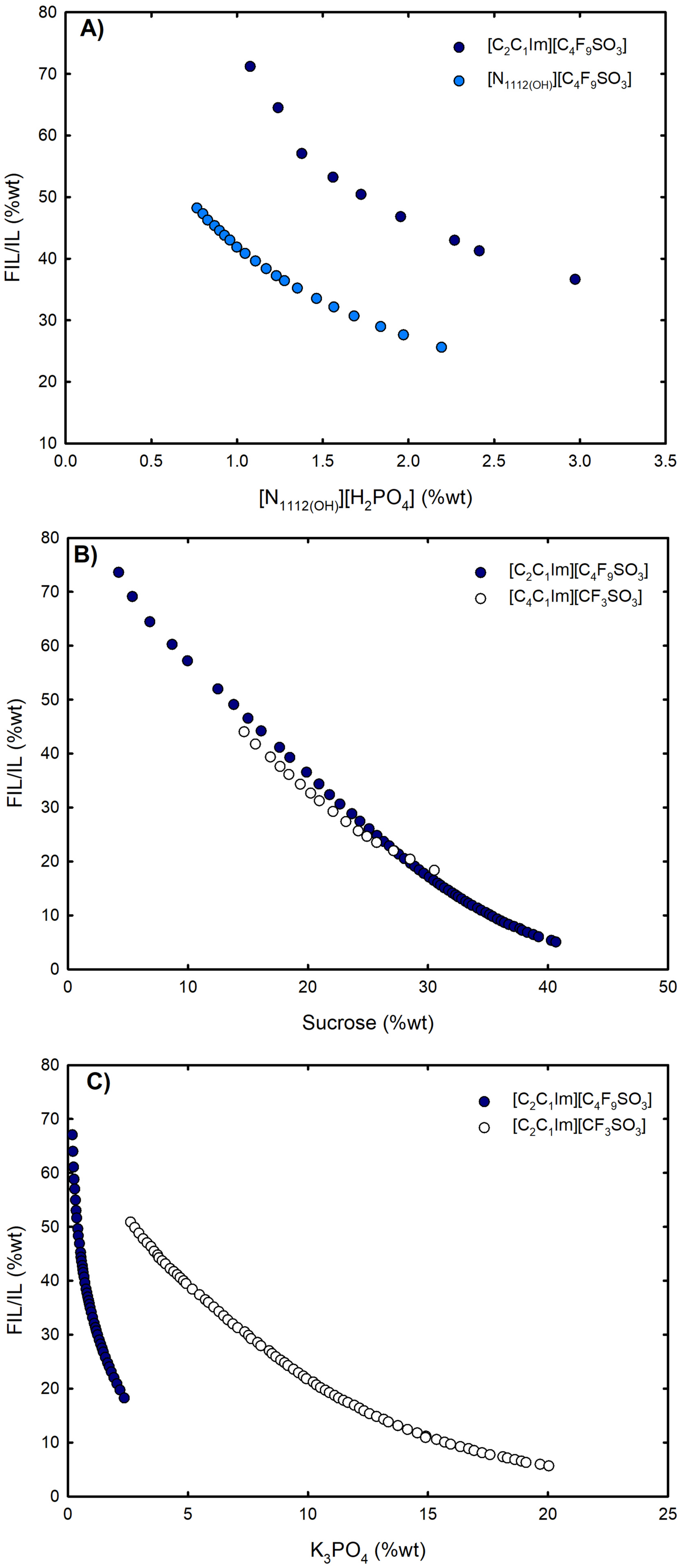

Figure S1. Ternary phase diagrams for ABS composed of A) $[C_2C_1Im][C_4F_9SO_3]$ or $[N_{1112(OH)}][C_4F_9SO_3]$ + $[N_{1112(OH)}][H_2PO_4]$ + water, B) $[C_2C_1Im][C_4F_9SO_3]$ or $[C_4C_1Im][CF_3SO_3]$ + Sucrose + water, and C) $[C_2C_1Im][C_4F_9SO_3]$ or $[C_2C_1Im][CF_3SO_3]$ + K_3PO_4 + water, at 25 °C and atmospheric pressure [1]. Demixing is not verified for $[C_2C_1Im][CF_3SO_3]$ with sucrose [1]. The studied BPs are detailed in Table 1.

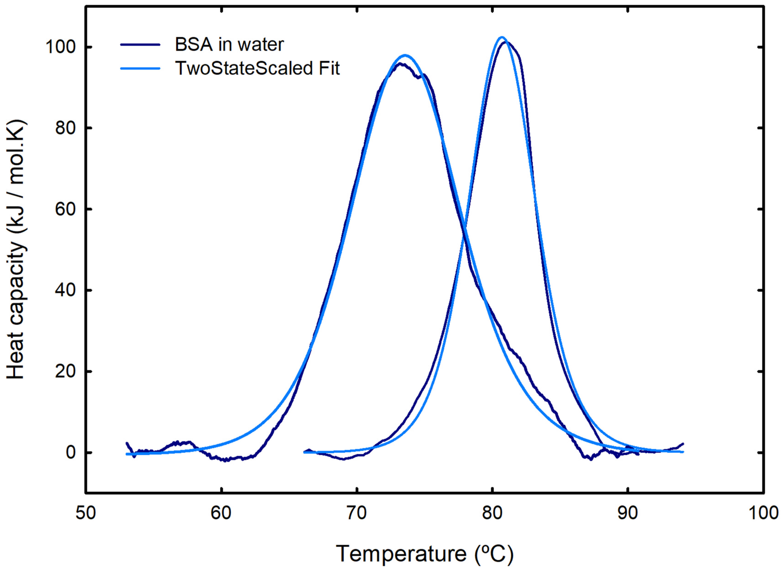

Figure S2. DSC curve and Two State Scaled Model fit of 1 mg/mL BSA in water. The scan rate was 1.0 °C/min with Heat capacity (Cp) as a function of Temperature, exo-up. Two experiments are plotted, to illustrate the variance verified due to BSA dimer (BSA was used as received, without further purification, size exclusion chromatography was not performed in order to isolate BSA monomer and ensure its monodispersity as done in a previous work of the authors [2]). The obtained BSA T_m of the different attained experiments is 75.59±3.68 °C (Table 2).

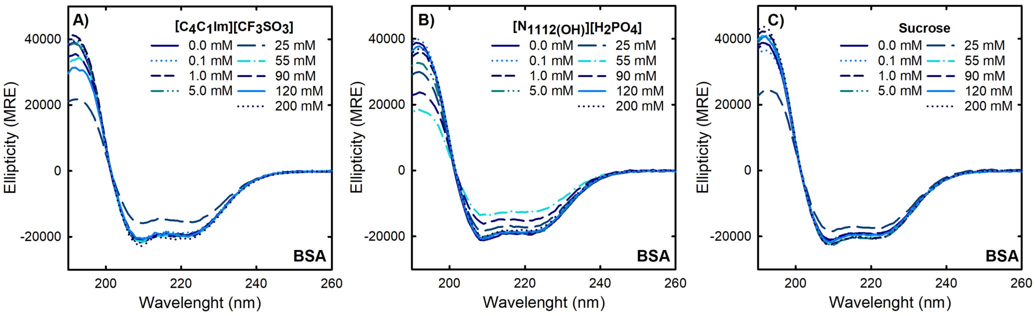

Figure S3. CD spectra of BSA 1.0 mg/ml in water, A) 0.1 mM - 200 mM $[C_4C_1Im][CF_3SO_3]$ B) 0.1 mM - 200 mM $[N_{1112(OH)}][H_2PO_4]$ and C) 0.1 mM - 200 mM sucrose. All spectra were acquired at 25 °C.

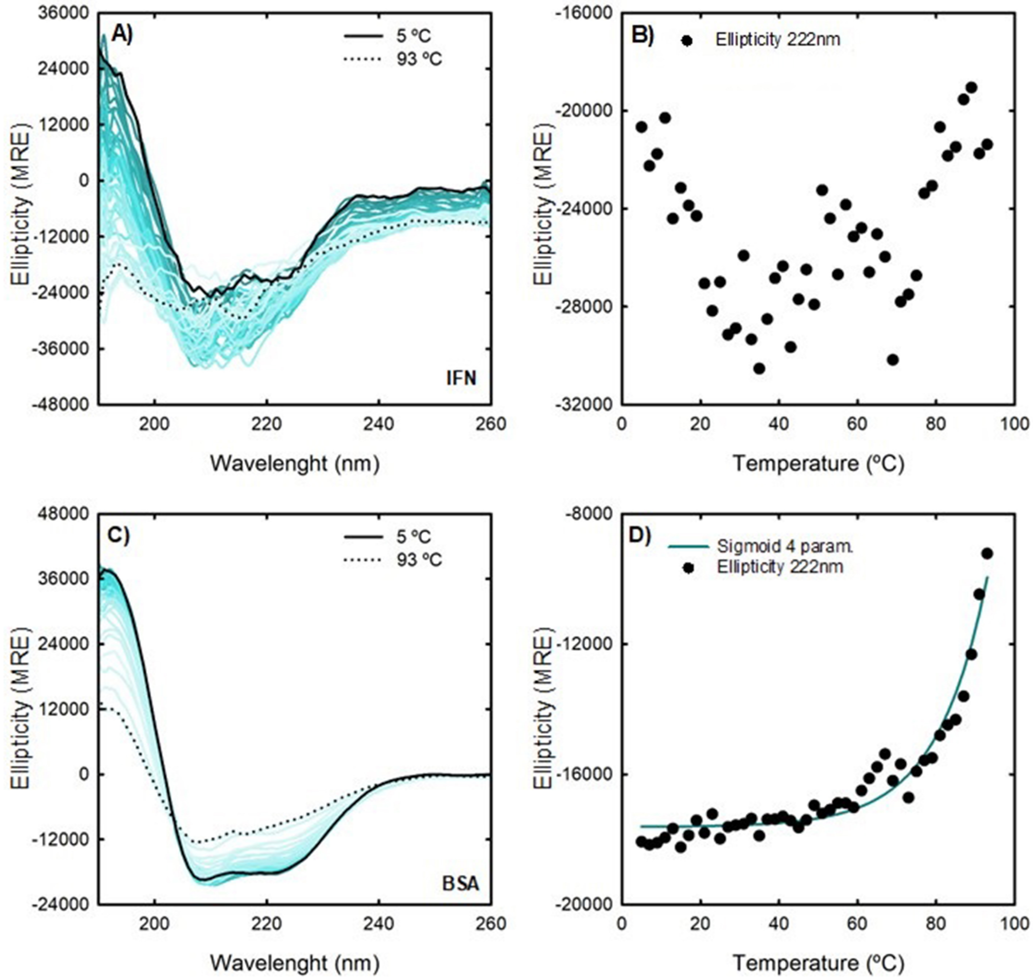

Figure S4. Spectra of A) IFN-α2b and C) BSA in 200 mM [C_2C_1Im][$C_4F_9SO_3$] collected as a function of temperature from 5 °C (full black) to 93 °C (dotted black). Ellipticity values for each temperature curve at 222nm (symbols) and fitting with sigmoid 4 parameters for B) IFN-α2b (no fitting) and D) BSA.

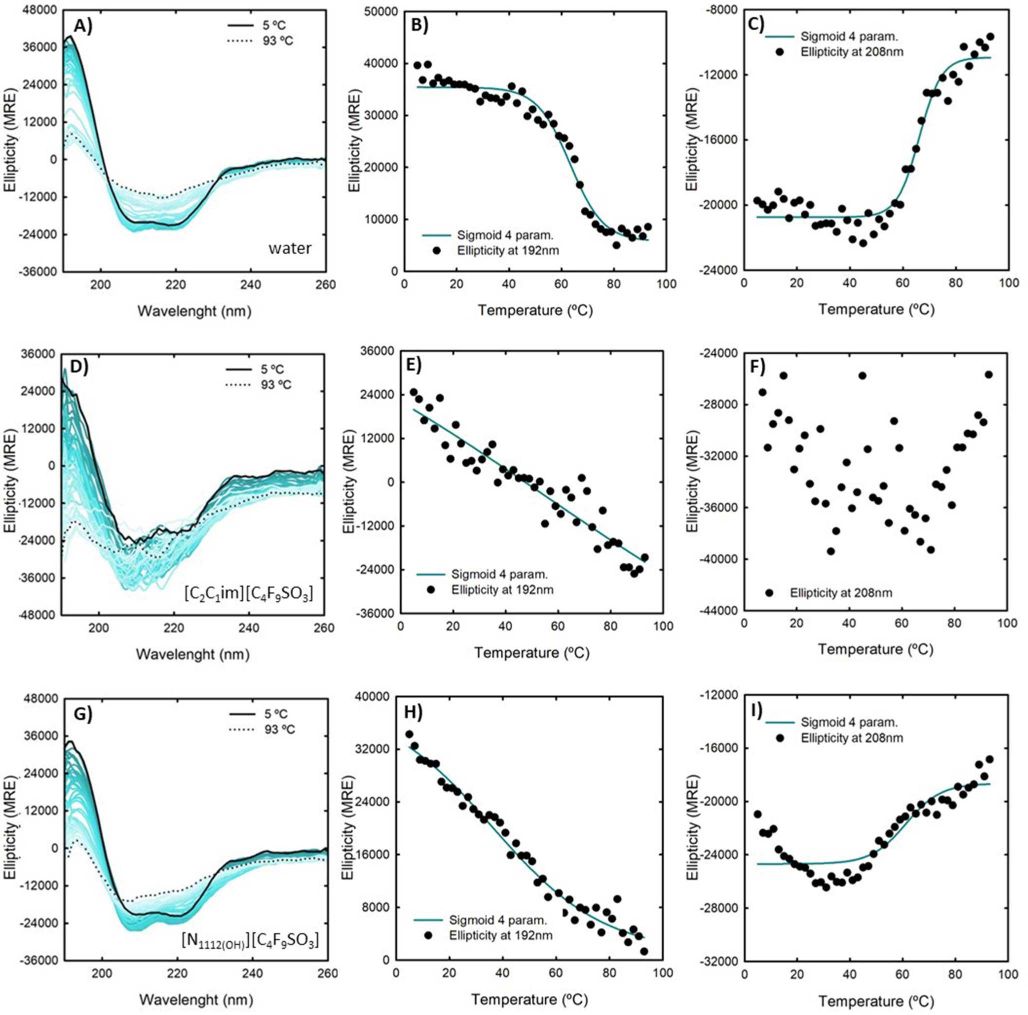

Figure S5. Spectra of IFN-α2b in A) water, and in 200 mM D) $[C_2C_1Im][C_4F_9SO_3]$ and G) $[N_{1112(OH)}][C_4F_9SO_3]$ collected as a function of temperature from 5 °C (full black) to 93 °C (dotted black). Ellipticity values for each temperature curve at 192nm and 208nm (symbols) and fitting with sigmoid 4 parameters for B) and C) water, E) and F) $[C_2C_1Im][C_4F_9SO_3]$ and H) and I) $[N_{1112(OH)}][C_4F_9SO_3]$ respectively.

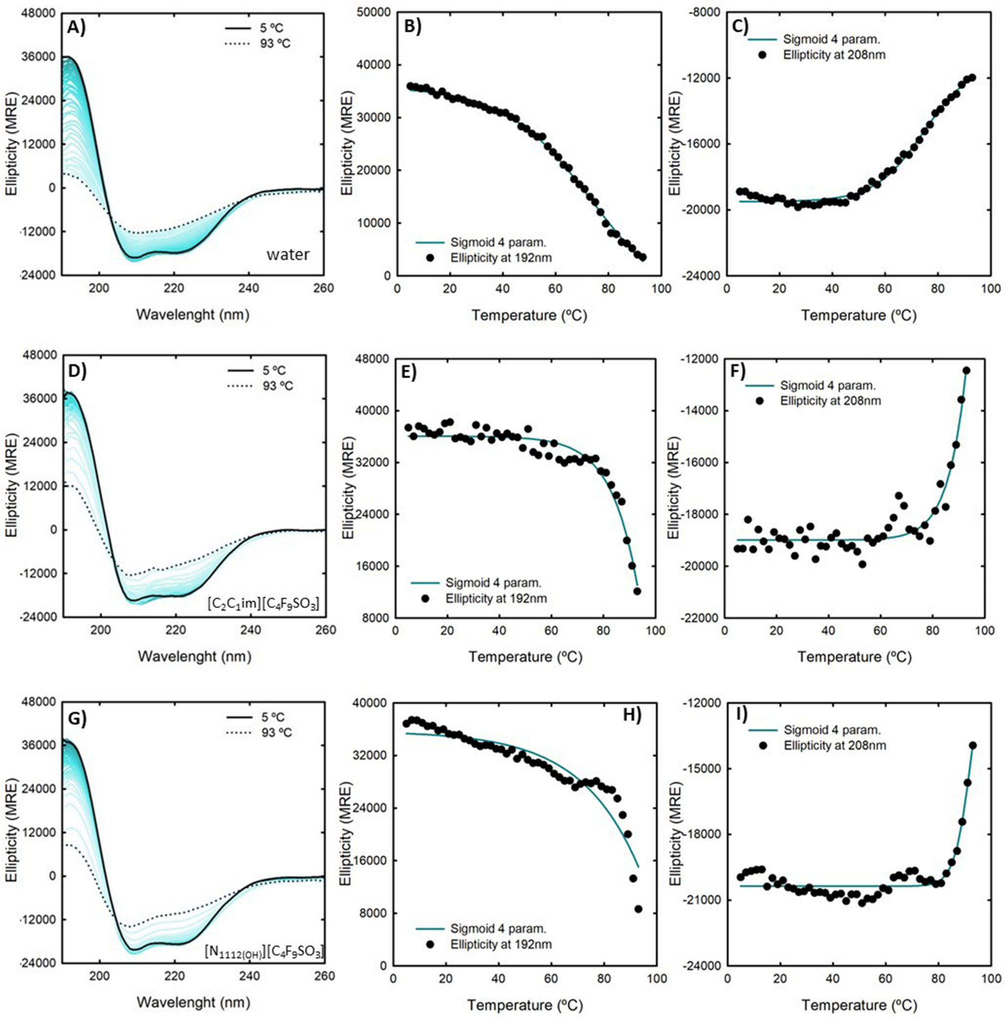

Figure S6. Spectra of BSA in A) water, and in 200 mM D) $[C_2C_1Im][C_4F_9SO_3]$ and G) $[N_{1112(OH)}][C_4F_9SO_3]$ collected as a function of temperature from 5 °C (full black) to 93 °C (dotted black). Ellipticity values for each temperature curve at 192nm and 208nm (symbols) and fitting with sigmoid 4 parameters for B) and C) water, E) and F) $[C_2C_1Im][C_4F_9SO_3]$ and H) and I) $[N_{1112(OH)}][C_4F_9SO_3]$ respectively.

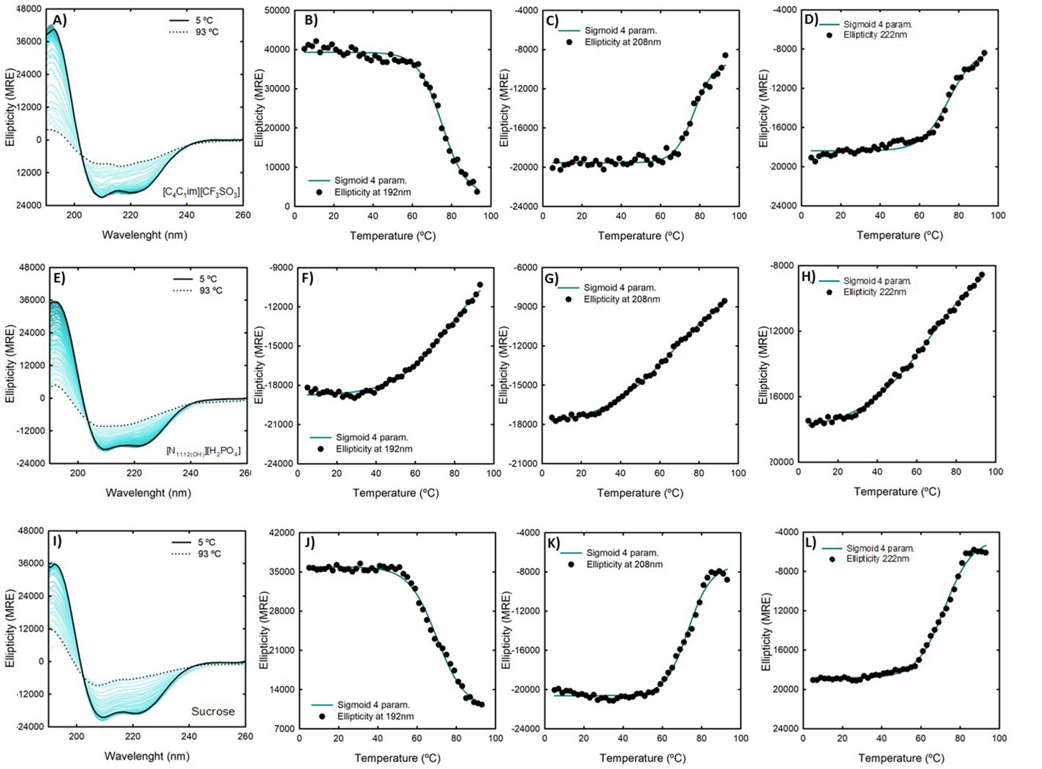

Figure S7. Spectra of BSA in 200 mM of A) $[C_4C_1Im][CF_3SO_3]$, E) $[N_{1112(OH)}][H_2PO_4]$ and I) sucrose collected as a function of temperature from 5 °C (black) to 93 °C (dotted black). Ellipticity values for each temperature curve at 192nm, 208nm and 222nm (symbols) and fitting with sigmoid 4 parameters for B), C) and D) $[C_4C_1Im][CF_3SO_3]$ F), G) and H) $[N_{1112(OH)}][H_2PO_4]$ and J), K) and L) sucrose, respectively.

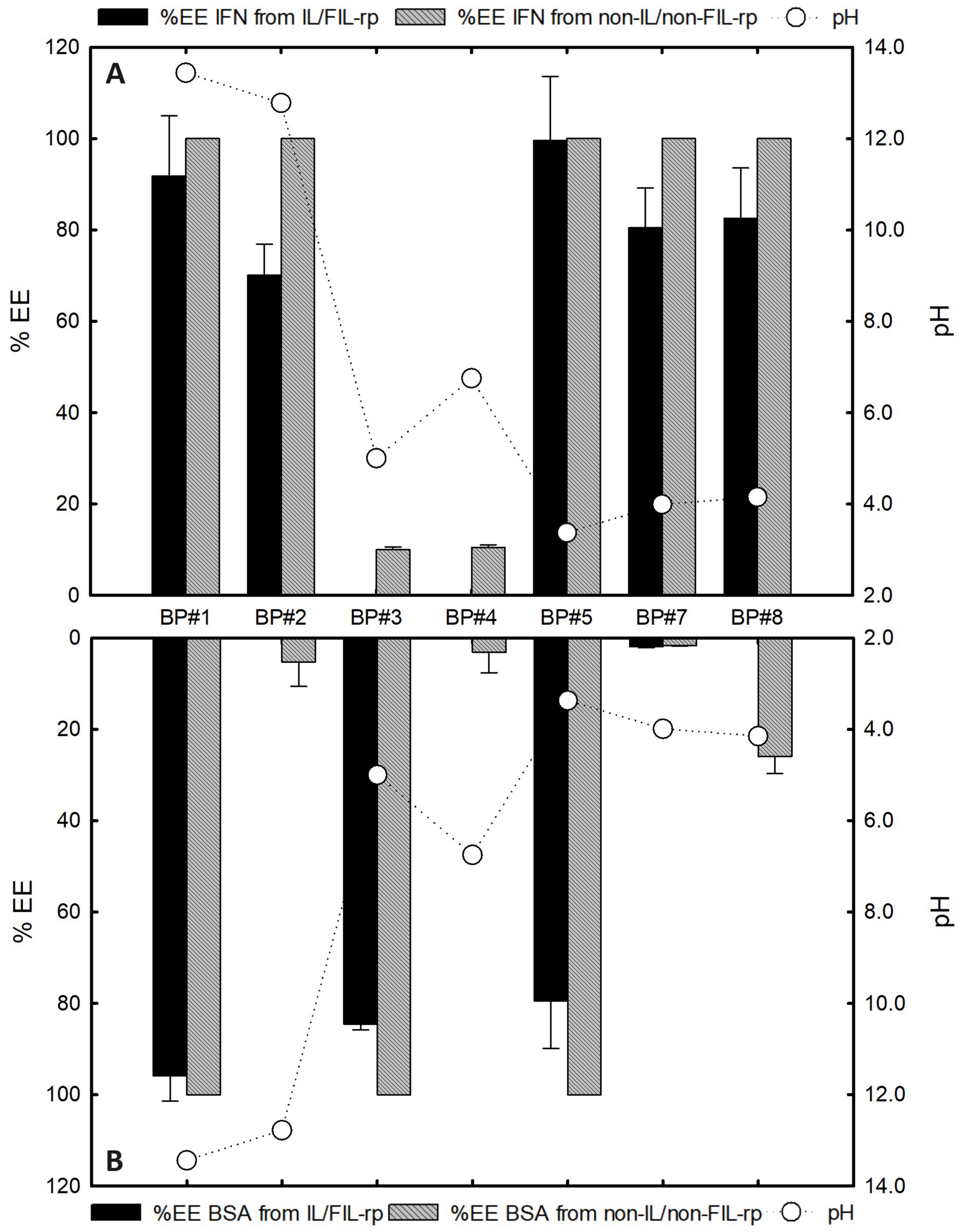

Figure S8. A) IFN-α2b and B) BSA extraction efficiencies (%EE) and pH of ionic liquid (fluorinated IL (FIL) or mere fluoro-containing IL; %wt) in each phase of the studied biphasic points, BP#1-BP#8. All pH and %EE values are summarized in Tables 1 and 4, respectively.

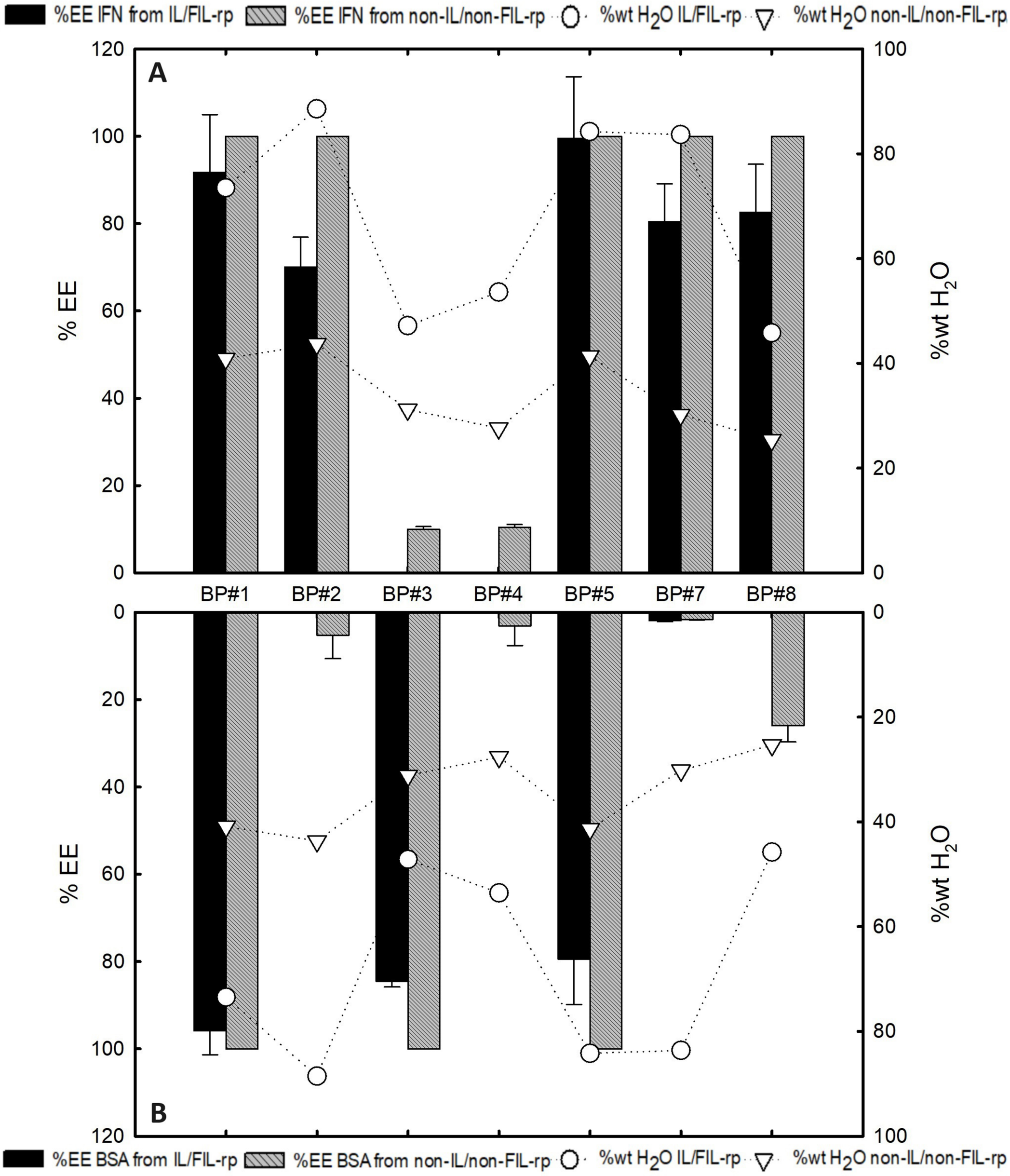

Figure S9. A) IFN-α2b and B) BSA extraction efficiencies (%EE; Equation (1)) and amount of water (%wt) in each phase of the studied biphasic points, BP#1-BP#8. All %wt water and %EE values are summarized in Tables 1 and 4, respectively.

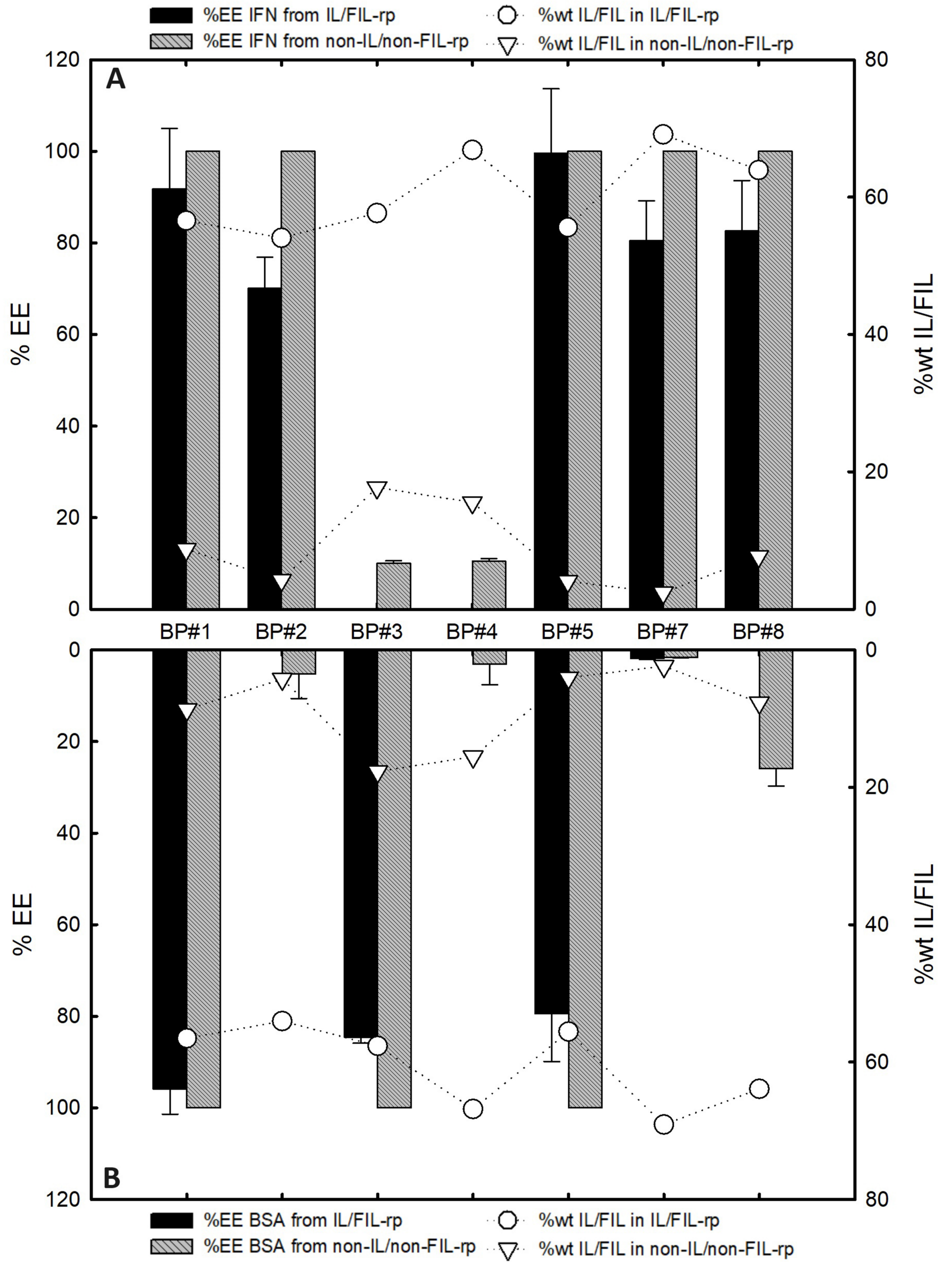

Figure S10. A) IFN-α2b and B) BSA extraction efficiencies (%EE) and amount of ionic liquid (fluorinated IL (FIL) or mere fluoro-containing IL; %wt) in each phase of the studied biphasic points, BP#1-BP#8. All %wt ionic liquid and %EE values are summarized in Tables 1 and 4, respectively.

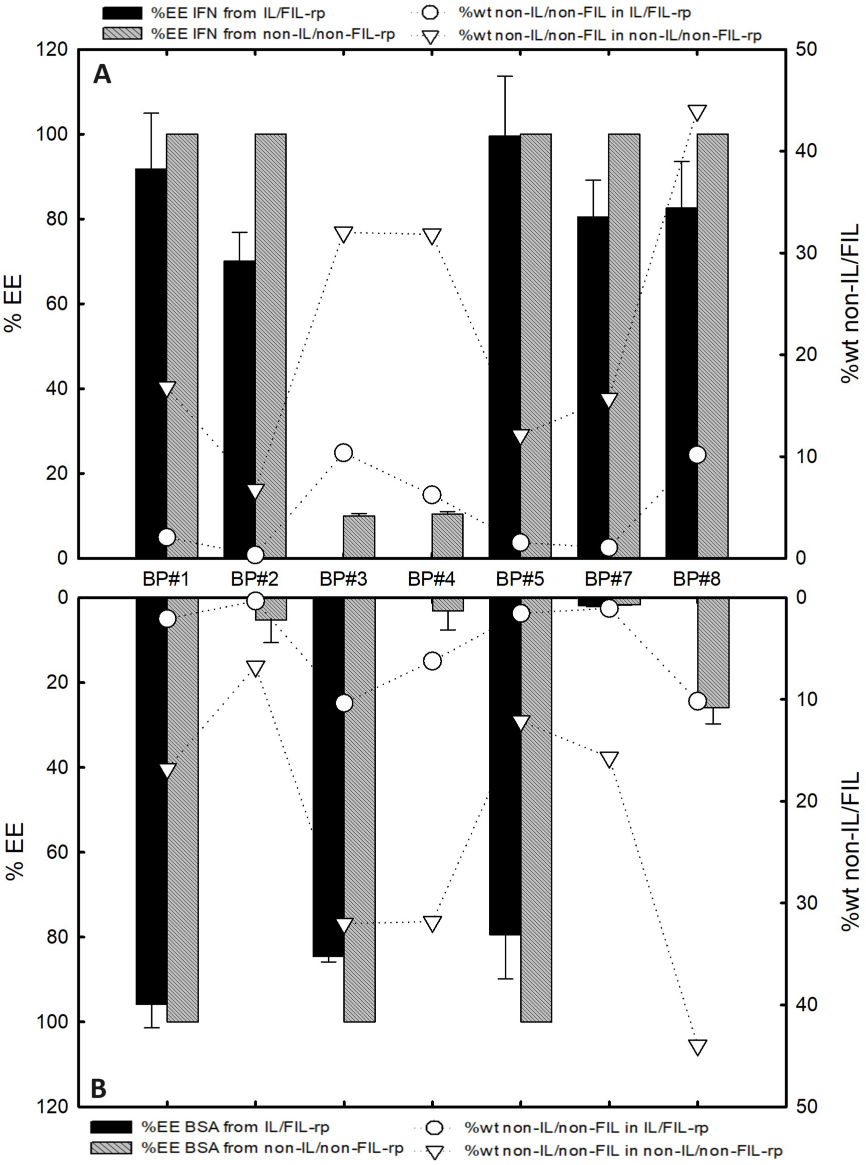

Figure S11. A) IFN-α2b and B) BSA extraction efficiencies (%EE) and amount of non-ionic liquid (non-fluorinated IL (non-FIL) or non-mere fluoro-containing (non-IL); %wt) in each phase of the studied biphasic points, BP#1-BP#8. All %wt non-ionic liquid and %EE values are summarized in Tables 1 and 4, respectively.

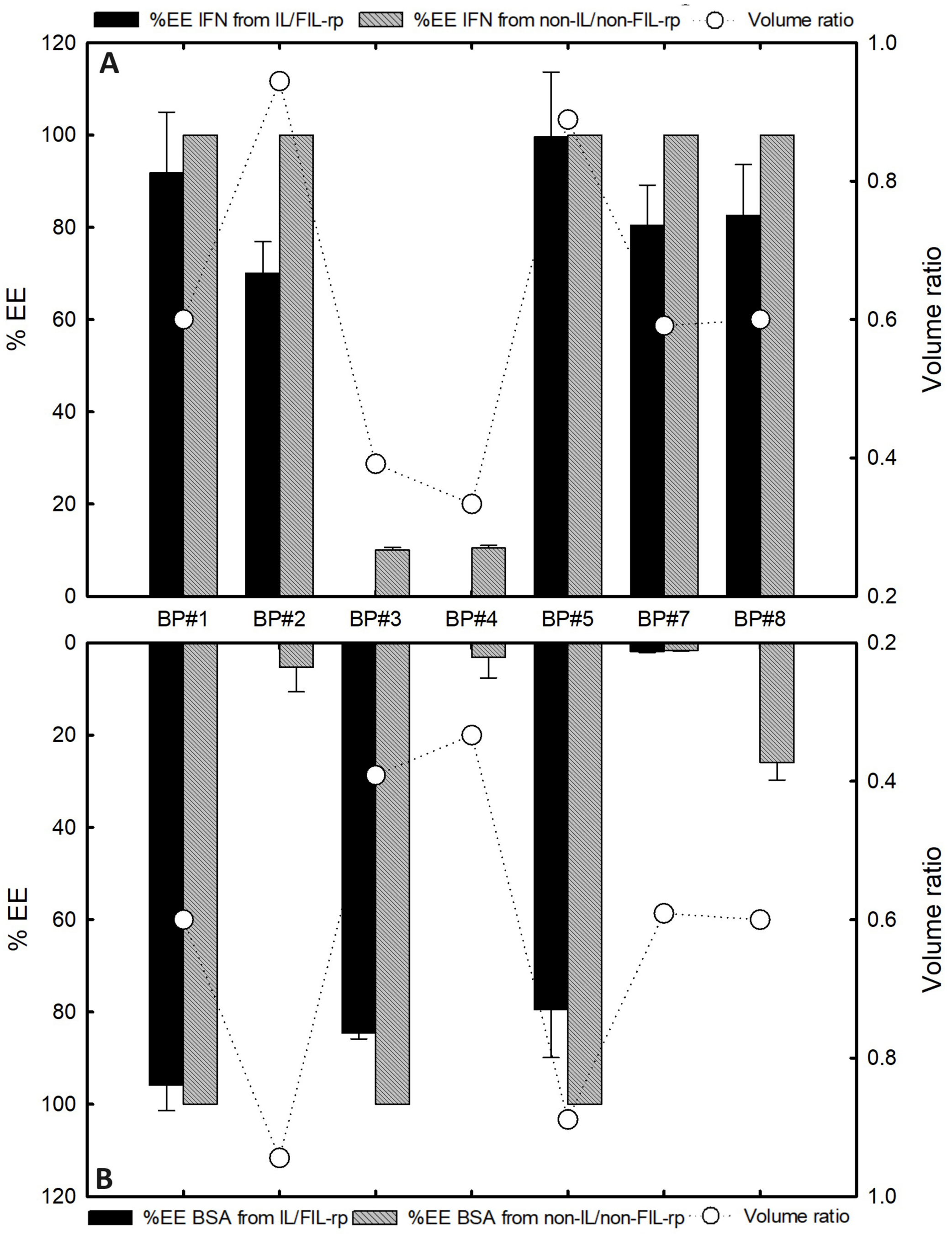

Figure S12. A) IFN-α2b and B) BSA extraction efficiencies (%EE) and volume ratio (volume IL/FIL-rp/volume non-IL/FIL-rp) of the studied biphasic points, BP#1-BP#8. All volume ratio and %EE values are summarized in Tables 1 and 4, respectively.

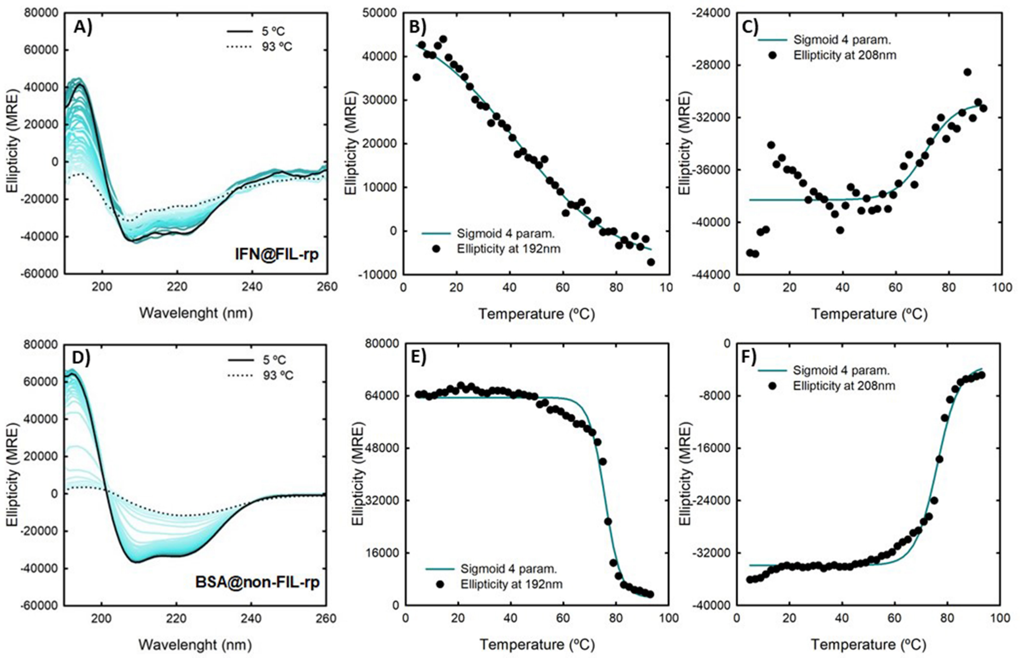

Figure S13. Spectra of A) IFN-α2b and D) BSA in FIL-rp and non-FIL-rp, respectively, of the biphasic point 30 %wt $[N_{1112(OH)}][C_4F_9SO_3]$ + 30 %wt $[N_{1112(OH)}][H_2PO_4]$ (BP#8) collected as a function of temperature from 5 °C (full black) to 93 °C (dotted black). Ellipticity values for each temperature curve at 192 nm and 208 nm (symbols) and fitting with sigmoid 4 parameters for B) and C) IFN, and E) and F) BSA, respectively.

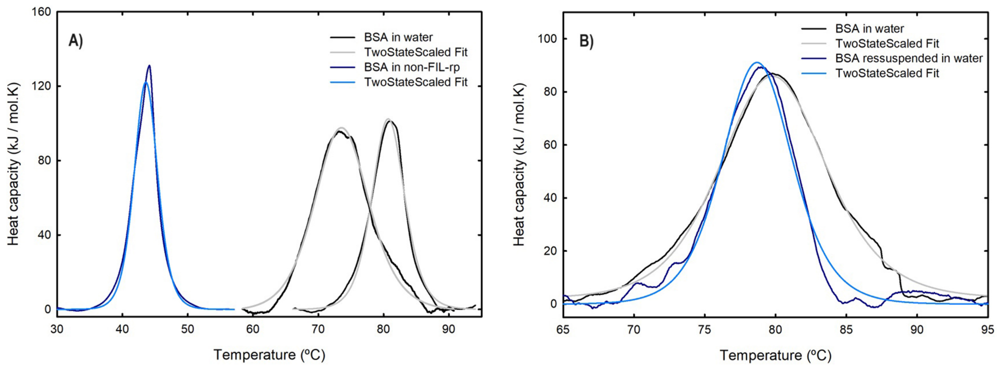

Figure S14. DSC curve and Two State Scaled Model fit of A) 1 mg/mL BSA in water (reference) and 1.2-1.6 mg/ml BSA in $[N_{1112(OH)}][H_2PO_4]$-rich phase (partition of 1.0 mg/ml BSA in BP#8, 30 %wt $[N_{1112(OH)}][C_4F_9SO_3]$ +30 %wt $[N_{1112(OH)}][H_2PO_4]$) and B) 0.25 mg/mL BSA in water (reference) and 0.25 mg/ml BSA resuspended in water (recovered from $[C_2C_1Im][C_4F_9SO_3]$-rich phase of BP#5 in the partition of 1.0 mg/ml BSA, 30 %wt $[C_2C_1Im][C_4F_9SO_3]$ + 6 % wt$[N_{1112(OH)}][H_2PO_4]$, and re-suspended in water). The scan rate was 1.0 °C/min with Heat capacity (Cp) as a function of Temperature, exo-up. All BSA T_m are summarized in Table 6.

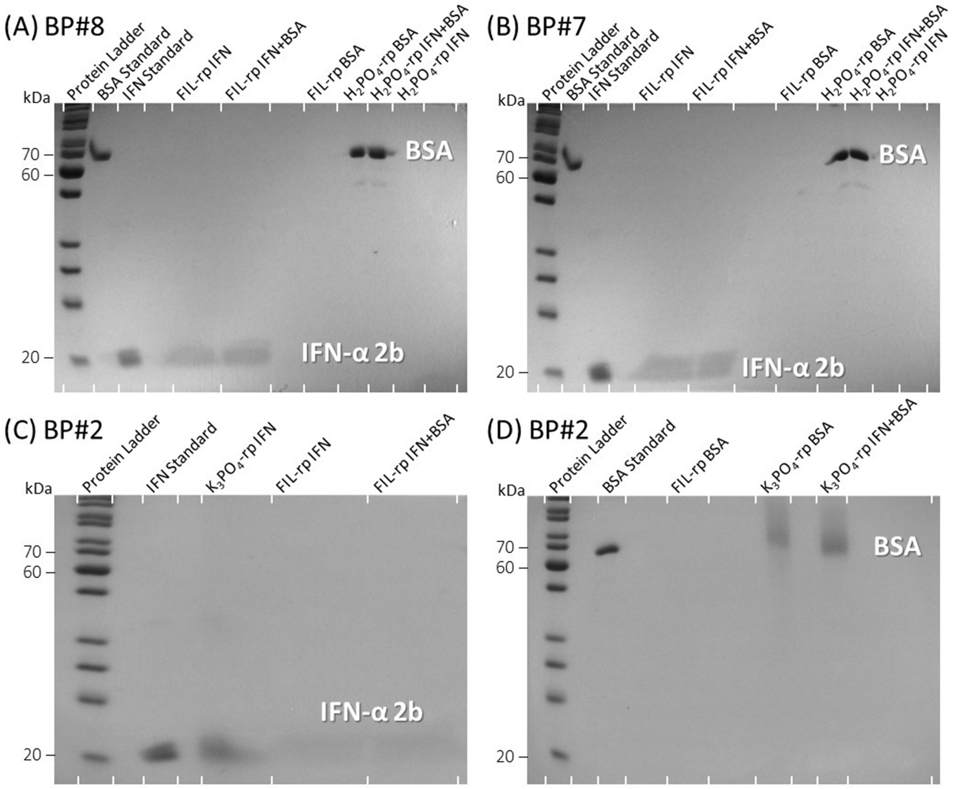

Figure S15. Monochromatic version of SDS-PAGE analysis of standard protein marker (protein ladder), IFN-α2b and BSA standard, FIL-rich phase and non-FIL-rich phase ([$N_{1112(OH)}$][H_2PO_4]-rich phase and K_3PO_4-rich phase) sample from individual partition (lane label, IFN or BSA), and FIL-rich phase and non-FIL-rich phase sample from simultaneous partition (lane label, IFN+BSA) stained with Coomassie blue. A) BP#8, 30 %wt [$N_{1112(OH)}$][$C_4F_9SO_3$] + 30 %wt [$N_{1112(OH)}$][H_2PO_4]. B) BP#7, 30 %wt [C_2C_1Im][$C_4F_9SO_3$] + 20 %wt [$N_{1112(OH)}$][H_2PO_4]. C) and D) BP#2, 30 %wt [C_2C_1Im][$C_4F_9SO_3$] + 2 %wt K_3PO_4. All SDS-PAGE profiles are identified at the top of each gel image. A detailed identification of all lanes is summarized in Table S3 of Supplementary Materials.

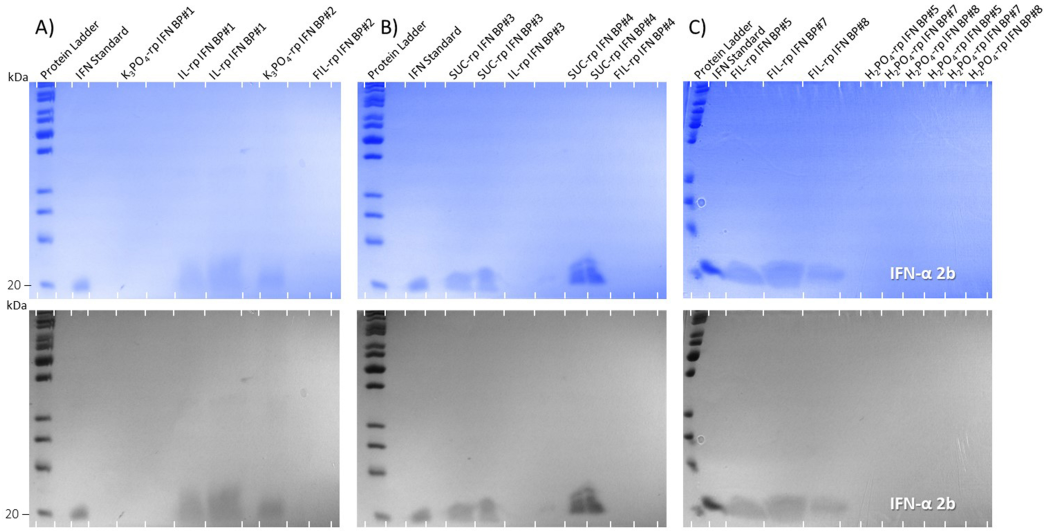

Figure S16. SDS-PAGE analysis of standard protein marker (protein ladder), IFN-α2b standard, and ionic liquid-rich phase ([C_2C_1Im][CF_3SO_3]-rich phase, [C_4C_1Im][CF_3SO_3]-rich phase, [C_2C_1Im][$C_4F_9SO_3$]-rich phase, and [$N_{1112(OH)}$][$C_4F_9SO_3$]-rich phase) and non-ionic liquid-rich phase (K_3PO_4-rich phase, sucrose-rich phase, and [$N_{1112(OH)}$][H_2PO_4]-rich phase) sample from individual partition of IFN-α2b (lane label, IFN) stained with Coomassie blue. The monochromatic version is also depicted in the bottom row. A) BP#1, 30 %wt [C_2C_1Im][CF_3SO_3] + 10 %wt K_3PO_4, and BP#2, BP#2, 30 %wt [C_2C_1Im][$C_4F_9SO_3$] + 2 %wt K_3PO_4. B) BP#3, 30 %wt [C_4C_1Im][CF_3SO_3] + 25 %wt sucrose, and BP#4, 30 %wt [C_2C_1Im][$C_4F_9SO_3$] + 25 %wt sucrose. C) BP#5, 30 %wt [C_2C_1Im][$C_4F_9SO_3$] + 6 %wt [$N_{1112(OH)}$][H_2PO_4], BP#7, 30 %wt [C_2C_1Im][$C_4F_9SO_3$] + 20 %wt [$N_{1112(OH)}$][H_2PO_4], and BP#8, 30 %wt [$N_{1112(OH)}$][$C_4F_9SO_3$] + 30 %wt [$N_{1112(OH)}$][H_2PO_4]. All SDS-PAGE profiles are identified at the top of each gel image. A detailed identification of all lanes is summarized in Table S4 of Supplementary Materials.

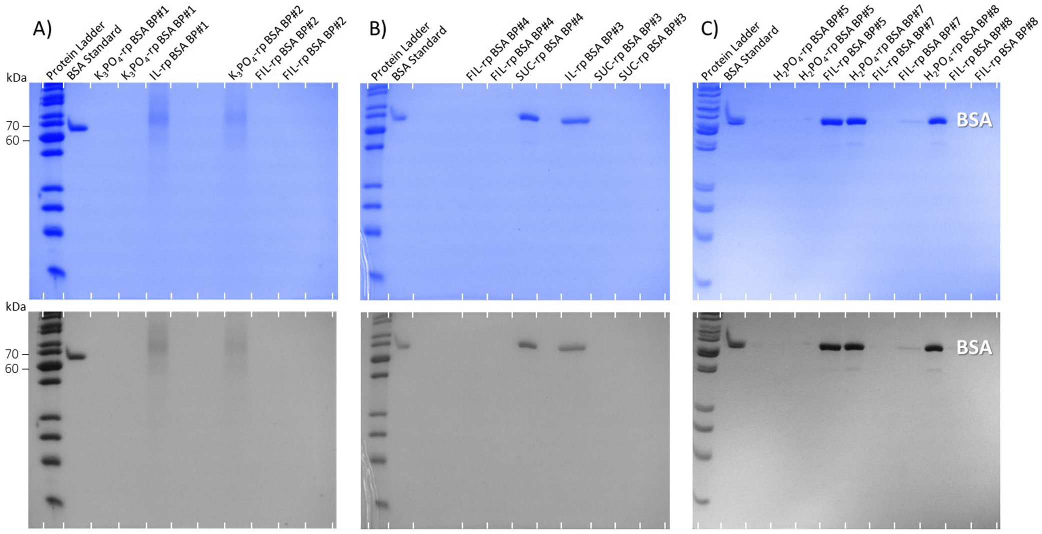

Figure S17. SDS-PAGE analysis of standard protein marker (protein ladder), BSA standard, and ionic liquid-rich phase ([C_2C_1Im][CF_3SO_3]-rich phase, [C_4C_1Im][CF_3SO_3]-rich phase, [C_2C_1Im][$C_4F_9SO_3$]-rich phase, and [$N_{1112(OH)}$][$C_4F_9SO_3$]-rich phase) and non-ionic liquid-rich phase (K_3PO_4-rich phase, sucrose-rich phase, and [$N_{1112(OH)}$][H_2PO_4]-rich phase) sample from individual partition of BSA (lane label, BSA) stained with Coomassie blue. The monochromatic version is also depicted in the bottom row. A) BP#1, 30 %wt [C_2C_1Im][CF_3SO_3] + 10 %wt K_3PO_4, and BP#2, 30 %wt [C_2C_1Im][$C_4F_9SO_3$] + 2 %wt K_3PO_4. B) BP#3, 30 %wt [C_4C_1Im][CF_3SO_3] + 25 %wt sucrose, and BP#4, 30 %wt [C_2C_1Im][$C_4F_9SO_3$] + 25 %wt sucrose. C) BP#5, 30 %wt [C_2C_1Im][$C_4F_9SO_3$] + 6 %wt [$N_{1112(OH)}$][H_2PO_4], BP#7, 30 %wt [C_2C_1Im][$C_4F_9SO_3$] + 20 %wt [$N_{1112(OH)}$][H_2PO_4], and BP#8, 30 %wt [$N_{1112(OH)}$][$C_4F_9SO_3$] + 30 %wt [$N_{1112(OH)}$][H_2PO_4]. All SDS-PAGE profiles are identified at the top of each gel image. A detailed identification of all lanes is summarized in Table S5 of Supplementary Materials.

Table S1. Critical Aggregation Concentration, CAC, of $[C_2C_1Im][C_4F_9SO_3]$and $[N_{1112(OH)}][C_4F_9SO_3]$in aqueous solution at 25 °C [3].

	$[C_2C_1Im][C_4F_9SO_3]$(mM)†	$[N_{1112(OH)}][C_4F_9SO_3]$(mM)‡
1st CAC	14.40	16.02
2nd CAC	34.48	35.17
3rd CAC	76.54	185.65
4th CAC	106.09	n.d.

†Determined by ITC; ‡Determined by Conductometry.

Table S2. T_m (°C) of 0.125 mg/ml IFN-α2b and 1.0 mg/ml BSA, determined using CD spectra thermal analysis, in water and 200 mM ABS phase-forming component aqueous solution (Figure 3 and Figure S7 of Supplementary Materials).

		Tm (°C)		
		Ellipticity 222nm	Ellipticity 208nm	Ellipticity 192nm
IFN	Water	67.548	66.156	63.311
	$[N_{1112(OH)}][C_4F_9SO_3]$	56.542	61.234	35.992
	$[C_2C_1Im][C_4F_9SO_3]$	n.d.	n.d.	53.633
BSA	Water	76.277	74.616	74.028
	$[N_{1112(OH)}][C_4F_9SO_3]$	176.497	92.409	282.372
	$[C_2C_1Im][C_4F_9SO_3]$	253.191	169.565	179.050
	$[C_4C_1Im][CF_3SO_3]$	74.833	77.089	75.050
	$[N_{1112(OH)}][H_2PO_4]$	66.702	76.945	65.691
	Sucrose	71.977	73.063	70.098

Table S3. BSA extraction efficiency (%EE; Equation (1)) for the ABS systems BP#2 (30 %wt $[C_2C_1Im][C_4F_9SO_3]$ + 2 %wt K_3PO_4), BP#3 (30 %wt $[C_2C_1Im][C_4F_9SO_3]$ + 25 %wt sucrose) and BP#8 (30 %wt $[N_{1112(OH)}][C_4F_9SO_3]$ + 30 %wt $[N_{1112(OH)}][H_2PO_4]$). Protein concentration in both ABS phases quantified by BCA and bradford coomassie protein assay. The %EE are the result of at least 3 partition experiments.

		**BCA**		**bradford coomassie**	
BP#	ABS system composition (%wt)	%EE (IL-rp)	%EE(non-IL-rp)	%EE (IL-rp)	%EE(non-IL-rp)
BP#2	30% $[C_2C_1Im][C_4F_9SO_3]$ + 2% K_3PO_4	0.00 ± 5.23	5.26 ± 0.00	0.00†	0.00†
BP#4	30% $[C_2C_1Im][C_4F_9SO_3]$ + 25% Sucrose	0.00†	0.41 ± 0.41	3.66 ± 0.09	0.00†
BP#8	30% $[N_{1112(OH)}][C_4F_9SO_3]$ + 30% $[N_{1112(OH)}][H_2PO_4]$	0.00†	13.69 ± 7.15	n.d.	n.d.

†no standard deviation due to protein quantification under detection limit (0.02 mg/mL, BCA; 0.001 mg/mL, bradford coomassie).

Table S4. Discriminated identification of the samples loaded in each of the lanes corresponding to the gels displayed in Figure 8 and Figure S15 of Supplementary Materials (monochromatic version).

SDS-PAGE	lane identification
Figure 8 A<br>BP#8	lane 1, protein ladder; lane 2, BSA standard solution; lane 3, IFN standard solution;<br>lane 4, empty lane; lane 5, $[N_{1112(OH)}][C_4F_9SO_3]$-rp individual IFN partition;<br>lane 6, $[N_{1112(OH)}][C_4F_9SO_3]$-rp IFN + BSA partition; lane 7, empty lane;<br>lane 8, $[N_{1112(OH)}][C_4F_9SO_3]$-rp individual BSA partition;<br>lane 9, $[N_{1112(OH)}][H_2PO_4]$-rp individual BSA partition;<br>lane 10, $[N_{1112(OH)}][H_2PO_4]$-rp IFN+BSA partition;<br>lane 11, $[N_{1112(OH)}][H_2PO_4]$-rp individual IFN partition; lane 12, empty lane.
Figure 8 B<br>BP#7	lane 1, protein ladder; lane 2, BSA standard solution; lane 3, IFN standard solution;<br>lane 4, empty lane; lane 5, $[C_2C_1Im][C_4F_9SO_3]$-rp individual IFN partition;<br>lane 6, $[C_2C_1Im][C_4F_9SO_3]$-rp IFN+BSA partition; lane 7, empty lane;<br>lane 8, $[C_2C_1Im][C_4F_9SO_3]$-rp individual BSA partition;<br>lane 9, $[N_{1112(OH)}][H_2PO_4]$-rp individual BSA partition;<br>lane 10, $[N_{1112(OH)}][H_2PO_4]$-rp IFN+BSA partition;<br>lane 11, $[N_{1112(OH)}][H_2PO_4]$-rp individual IFN partition; lane 12, empty lane.
Figure 8 C<br>BP#2 IFN-rich phase	lane 1, protein ladder; lane 2, empty lane; lane 3, IFN standard solution;<br>lane 4, K_3PO_4-rp individual IFN partition;<br>lane 5, $[C_2C_1Im][C_4F_9SO_3]$-rp individual IFN partition; lane 6, empty lane;<br>lane 7, $[C_2C_1Im][C_4F_9SO_3]$-rp IFN+BSA partition; lane 8, empty lane.
Figure 8 D<br>BP#2 BSA-rich phase	lane 1, protein ladder; lane 2, empty lane; lane 3, BSA standard solution;<br>lane 4, empty lane; lane 5, $[C_2C_1Im][C_4F_9SO_3]$-rp individual BSA partition;<br>lane 6, empty lane; lane 7, K_3PO_4-rp individual BSA partition; lane 8, empty lane;<br>lane 9, K_3PO_4-rp IFN+BSA partition; lane 10, empty lane.

Table S5. Discriminated identification of the samples loaded in each of the lanes corresponding to the gels displayed in Figure S16 of Supplementary Materials.

SDS-PAGE	lane identification
Figure S16 A<br>BP#1 and BP#2	lane 1, protein ladder; lane 2, empty lane; lane 3, IFN standard solution;<br>lane 4, empty lane; lane 5, K_3PO_4-rp BP#1‡; lane 6, empty lane;<br>lane 7, $[C_2C_1Im][CF_3SO_3]$-rp BP#1; lane 8, $[C_2C_1Im][CF_3SO_3]$-rp BP#1‡;<br>lane 9, empty lane; lane 10, K_3PO_4-rp BP#2‡;<br>lane 11, empty lane; lane 12, $[C_2C_1Im][C_4F_9SO_3]$-rp BP#2‡.
Figure S16 B<br>BP#3 and BP#4	lane 1, protein ladder; lane 2, empty lane; lane 3, IFN standard solution;<br>lane 4, empty lane; lane 5, Sucrose-rp BP#3; lane 6, Sucrose-rp BP#3‡;<br>lane 7, $[C_4C_1Im][CF_3SO_3]$-rp BP#3‡; lane 8, empty lane;<br>lane 9, Sucrose-rp BP#4; lane 10, Sucrose-rp BP#4‡;<br>lane 11, $[C_2C_1Im][C_4F_9SO_3]$-rp BP#4‡; lane 12, empty lane.
Figure S16 C<br>BP#5, BP#7 and BP#8	lane 1, protein ladder; lane 2, IFN standard solution;<br>lane 3, $[C_2C_1Im][C_4F_9SO_3]$-rp BP#5; lane 4, $[C_2C_1Im][C_4F_9SO_3]$-rp BP#7;<br>lane 5, $[N_{1112(OH)}][C_4F_9SO_3]$-rp BP#8; lane 6, empty lane;<br>lane 7, $[N_{1112(OH)}][H_2PO_4]$-rp BP#5†; lane 8, $[N_{1112(OH)}][H_2PO_4]$-rp BP#7†;<br>lane 9, $[N_{1112(OH)}][H_2PO_4]$-rp BP#8†; lane 10, $[N_{1112(OH)}][H_2PO_4]$-rp BP#5‡;<br>lane 11, $[N_{1112(OH)}][H_2PO_4]$-rp BP#7‡; lane 12, $[N_{1112(OH)}][H_2PO_4]$-rp BP#8‡.

†amount of protein lower than 0.5 μg, due to the used dilution factor equal to the counterpart ABS phase (0.5 μg of protein). counterpart ABS phase in adjacent lane, left or right.
‡amount of protein lower than 0.5 μg, or lower than 15 ng (determined detection limit), thus sampling without dilution. correspondent counterparts maintaining sampling without dilution (> 0.5 μg).

Table S6. Discriminated identification of the samples loaded in each of the lanes corresponding to the gels displayed in Figure S17 of Supplementary Materials.

SDS-PAGE	lane identification
Figure S17 A<br>BP#1 and BP#2	lane 1, protein ladder; lane 2, BSA standard solution; lane 3, K_3PO_4-rp BP#1;<br>lane 4, K_3PO_4-rp BP#1†; lane 5, $[C_2C_1Im][CF_3SO_3]$-rp BP#1; lane 6, empty lane;<br>lane 7, empty lane; lane 8, K_3PO_4-rp BP#2; lane 9, $[C_2C_1Im][C_4F_9SO_3]$-rp BP#2†;<br>lane 10, $[C_2C_1Im][C_4F_9SO_3]$-rp BP#2; lane 11, empty lane; and 12, empty lane.
Figure S17 B<br>BP#3 and BP#4	lane 1, protein ladder; lane 2, BSA standard solution; lane 3, empty lane;<br>lane 4, $[C_2C_1Im][C_4F_9SO_3]$-rp BP#4; lane 5, $[C_2C_1Im][C_4F_9SO_3]$-rp BP#4†;<br>lane 6, Sucrose-rp BP#4; lane 7, empty lane; lane 8, $[C_4C_1Im][CF_3SO_3]$-rp BP#3;<br>lane 9, Sucrose-rp BP#3; lane 10, Sucrose-rp BP#4†;<br>lane 11, empty lane; lane 12, empty lane.
Figure S17 C<br>BP#5, BP#7 and BP#8	lane 1, protein ladder; lane 2, BSA standard solution; lane 3, empty lane;<br>lane 4, $[N_{1112(OH)}][H_2PO_4]$-rp BP#5; lane 5, $[N_{1112(OH)}][H_2PO_4]$-rp BP#5†;<br>lane 6, $[C_2C_1Im][C_4F_9SO_3]$-rp BP#5; lane7, $[N_{1112(OH)}][H_2PO_4]$-rp BP#7;<br>lane 8, $[C_2C_1Im][C_4F_9SO_3]$-rp BP#7†; lane 9, $[C_2C_1Im][C_4F_9SO_3]$-rp BP#7;<br>lane 10, $[N_{1112(OH)}][H_2PO_4]$-rp BP#8; lane 11, $[N_{1112(OH)}][C_4F_9SO_3]$-rp BP#8†;<br>lane 12, $[N_{1112(OH)}][C_4F_9SO_3]$-rp BP#8.

†amount of protein lower than 0.5 μg, due to the used dilution factor equal to the counterpart ABS phase (0.5 μg of protein). counterpart ABS phase in adjacent lane, left or right.

Table S7. Name, acronym, and chemical structure of all ABS phase-forming components.

Name	Acronym	Chemical Structure
	**fluorinated ionic liquids**	
1-ethyl-3-methylimidazolium perfluorobutanesulfonate	$[C_2C_1Im][C_4F_9SO_3]$	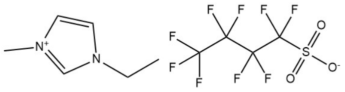
choline ((2-hydroxyethyl)trimethylammonium) perfluorobutanesulfonate	$[N_{1112(OH)}][C_4F_9SO_3]$	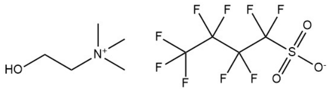
	**mere fluoro-containing ionic liquids**	
1-ethyl-3-methylimidazolium trifluoromethanesulfonate	$[C_2C_1Im][CF_3SO_3]$	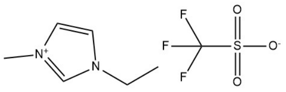
1-butyl-3-methylimidazolium trifluoromethanesulfonate	$[C_4C_1Im][CF_3SO_3]$	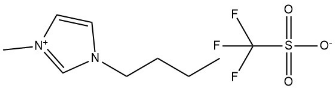
	**globular protein stabilizers**	
choline ((2-hydroxyethyl)trimethylammonium) dihydrogen phosphate	$[N_{1112(OH)}][H_2PO_4]$	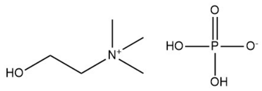
sucrose	Sucrose	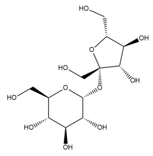
	**high-charge density salts**	
Potassium phosphate tribasic	K_3PO_4	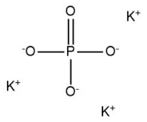

References

1. Carvalho, S.F.; Custódio, M.; Pereiro, A.B.; Araújo, J.M. Towards Enhanced Tunability of Aqueous Biphasic Systems: Furthering the Grasp of Fluorinated Ionic Liquids in the Purification of Proteins. *J. Mol. Liq.* **2023**, *Submitted*.
2. Alves, M.M.; Araújo, J.M.; Martins, I.C.; Pereiro, A.B.; Archer, M. Insights into the interaction of Bovine Serum Albumin with Surface-Active Ionic Liquids in aqueous solution. *Journal of Molecular Liquids* **2021**, *322*. https://doi.org/10.1016/j.molliq.2020.114537.
3. Pereiro, A.B.; Araújo, J.M.; Teixeira, F.S.; Marrucho, I.M.; Piñeiro, M.M.; Rebelo, L.P.N. Aggregation behavior and total miscibility of fluorinated ionic liquids in water. *Langmuir* **2015**, *31*, 1283–1295. https://doi.org/10.1021/la503961h.

Disclaimer/Publisher's Note: The statements, opinions and data contained in all publications are solely those of the individual author(s) and contributor(s) and not of MDPI and/or the editor(s). MDPI and/or the editor(s) disclaim responsibility for any injury to people or property resulting from any ideas, methods, instructions or products referred to in the content.
